# Supplementary material for: Estimating Re and overdispersion in secondary cases from the size of identical sequence clusters of SARS-CoV-2
Source: PLoS Comput Biol. 2025 Apr 15;21(4):e1012960. doi: 10.1371/journal.pcbi.1012960 (PMC12040226; doi:10.1371/journal.pcbi.1012960)
Supplement: S1 Text — (PDF) [file pcbi.1012960.s001.pdf]

Supplementary material S1 Text to:
Estimating R_e and overdispersion in secondary cases from the size of identical sequence clusters of SARS-CoV-2

Emma B. Hodcroft[1,2,3◑¤], Martin S. Wohlfender[1,2,4◑], Richard A. Neher[3,5], Julien Riou[1,2,6], Christian L. Althaus[1,2]

1 Institute of Social and Preventive Medicine, University of Bern, Bern, Switzerland
2 Multidisciplinary Center for Infectious Diseases, University of Bern, Bern, Switzerland
3 Swiss Institute of Bioinformatics, Lausanne, Switzerland
4 Graduate School for Cellular and Biomedical Sciences, University of Bern, Bern, Switzerland
5 Biozentrum, University of Basel, Basel, Switzerland
6 Unisanté, University Center for Primary Care and Public Health, Department of Epidemiology and Health Systems, University of Lausanne, Lausanne, Switzerland

◑These authors contributed equally to this work.
¤Current Address: Swiss Tropical and Public Health Institute, Allschwil, Switzerland
* emma.hodcroft@unibas.ch

Contents

A Data **3**
A.1 Switzerland 3
A.2 Denmark 5
A.3 Germany 7

B Probability Theory **9**
B.1 Transmission within identical sequence clusters 9
B.2 Sum of independent negative binomial random variables 12

C Model of cluster size distribution **18**
C.1 Description of model 18
C.2 Additional theoretical background 20
C.3 Possible extensions of the model 21

D Prior distributions and fixed parameters **22**

E Simulations **24**
E.1 Coefficient of variation 25
E.2 Root mean square error 27
E.3 Coverage 29

F Additional results **30**
F.1 Switzerland 31
F.2 Denmark 32
F.3 Germany 33

G Sensitivity analysis **34**
G.1 Prior distribution for mutation probability and constant testing probability 35
G.2 Constant mutation probability and prior distribution for testing probability 39
G.3 Constant mutation probability and constant testing probability 44
G.4 Prior distribution for yearly mutation rate and prior distribution for testing probability . 48
G.5 Prior distribution for yearly mutation rate and constant testing probability 53

H Comparison with literature **57**

I Posterior predictive check **61**
I.1 Switzerland 61
I.2 Denmark 62
I.3 Germany 63

J Goodness of fit **64**
J.1 Switzerland 64
J.2 Denmark 65
J.3 Germany 66

K Bibliography **67**

A Data

We used sequence data collected during the SARS-CoV-2 epidemic in Switzerland, Denmark and Germany to estimate the effective reproduction number R_e and the dispersion parameter k on a monthly basis during 2021. We downloaded all available sequences of the respective country and period from GISAID [1] and grouped them into clusters of identical sequences. A cluster is assigned to a month if there is a sequence in the cluster that has been sampled in the month. The same cluster can therefore be assigned to several months. This is why the clusters assigned to a month contain strictly more sequences than sequences were sampled during the respective month. Furthermore, we retrieved daily numbers of newly confirmed cases from public sources of Switzerland [2], Denmark [3] and Germany [4] and summarized them to monthly values. The number of confirmed cases, the number of sampled sequences, the number of assigned identical sequence clusters, the number of sequences contained in the assigned identical sequence clusters, the sequencing coverage and the size of the largest assigned identical sequence cluster by country and month are presented in the following tables.

A.1 Switzerland

Month	Nocc	Nos	Nosic	Noc	Nos/Nocc	Solc
January	68,867	7,590	9,915	5,665	0.1102	114
February	32,110	4,213	6,367	2,712	0.1312	92
March	46,145	5,377	7,721	3,417	0.1165	92
April	58,135	7,485	10,226	4,957	0.1288	65
May	33,434	5,692	7,748	3,663	0.1702	65
June	7,381	1,586	3,335	1,178	0.2149	363
July	16,117	5,450	7,335	3,050	0.3382	363
August	64,669	12,456	15,772	8,389	0.1926	363
September	57,672	9,037	13,688	6,437	0.1567	363
October	35,422	9,030	13,967	5,848	0.2549	363
November	152,427	14,817	19,134	9,437	0.0972	204
December	324,389	10,700	16,277	7,989	0.0330	488
Total	896,768	93,433	131,485	62,742	0.1042	488

Table A. Overview of data from Switzerland.
Nocc: Number of confirmed cases Nos: Number of sampled sequences Nosic: Number of sequences in clusters Noc: Number of clusters Solc: Size of largest clusters.

	Cluster size									
**Month**	**1**	**2**	**3**	**4**	**5**	**6-10**	**11-50**	**51-100**	**101-488**	**All**
January	4,482	574	223	104	63	125	90	3	1	5,665
February	1,872	345	146	82	53	116	96	2	0	2,712
March	2,421	428	160	98	61	138	108	3	0	3,417
April	3,537	639	262	140	87	168	121	3	0	4,957
May	2,602	451	192	107	67	146	96	2	0	3,663
June	833	144	63	34	21	48	29	4	2	1,178
July	2,231	405	138	77	35	81	70	10	3	3,050
August	6,493	995	334	179	95	166	110	13	4	8,389
September	4,755	819	318	155	93	170	110	13	4	6,437
October	4,179	707	309	146	93	242	157	11	4	5,848
November	7,044	1,083	429	233	140	302	193	11	2	9,437
December	6,274	801	301	156	97	190	153	12	5	7,989
Total	46,723	7,391	2,875	1,511	905	1,892	1,333	87	25	62,742

Table B1. Identical sequence clusters from Switzerland.

	Cluster size									
**Month**	**1**	**2**	**3**	**4**	**5**	**6-10**	**11-50**	**51-100**	**101-488**	**All**
January	0.7912	0.1013	0.0394	0.0184	0.0111	0.0221	0.0159	0.0005	0.0002	1
February	0.6903	0.1272	0.0538	0.0302	0.0195	0.0428	0.0354	0.0007	0.0000	1
March	0.7085	0.1253	0.0468	0.0287	0.0179	0.0404	0.0316	0.0009	0.0000	1
April	0.7135	0.1289	0.0529	0.0282	0.0176	0.0339	0.0244	0.0006	0.0000	1
May	0.7103	0.1231	0.0524	0.0292	0.0183	0.0399	0.0262	0.0005	0.0000	1
June	0.7071	0.1222	0.0535	0.0289	0.0178	0.0407	0.0246	0.0034	0.0017	1
July	0.7315	0.1328	0.0452	0.0252	0.0115	0.0266	0.0230	0.0033	0.0010	1
August	0.7740	0.1186	0.0398	0.0213	0.0113	0.0198	0.0131	0.0015	0.0005	1
September	0.7387	0.1272	0.0494	0.0241	0.0144	0.0264	0.0171	0.0020	0.0006	1
October	0.7146	0.1209	0.0528	0.0250	0.0159	0.0414	0.0268	0.0019	0.0007	1
November	0.7464	0.1148	0.0455	0.0247	0.0148	0.0320	0.0205	0.0012	0.0002	1
December	0.7853	0.1003	0.0377	0.0195	0.0121	0.0238	0.0192	0.0015	0.0006	1
Total	0.7447	0.1178	0.0458	0.0241	0.0144	0.0302	0.0212	0.0014	0.0004	1

Table B2. Identical sequence clusters from Switzerland (proportion of clusters).

A.2 Denmark

Month	Nocc	Nos	Nosic	Noc	Nos/Nocc	Solc
January	32,069	14,784	29,882	5,273	0.4610	417
February	12,856	9,265	25,068	2,787	0.7207	926
March	19,131	16,159	27,755	4,196	0.8447	926
April	20,550	14,715	34,189	4,485	0.7161	926
May	29,713	23,142	35,922	6,077	0.7789	926
June	11,614	5,065	19,175	1,881	0.4361	760
July	23,788	19,769	28,362	7,459	0.8310	760
August	29,032	23,859	35,669	10,625	0.8218	518
September	12,528	10,767	23,179	5,102	0.8594	1,278
October	30,881	24,213	46,539	8,160	0.7841	1,278
November	106,733	49,645	77,490	18,872	0.4651	1,278
December	324,105	37,854	72,492	16,908	0.1168	1,278
Total	653,000	249,237	455,722	91,825	0.3817	1,278

Table C. Overview of data from Denmark.
Nocc: Number of confirmed cases Nos: Number of sampled sequences Nosic: Number of sequences in clusters Noc: Number of clusters Solc: Size of largest clusters.

	Cluster size									
**Month**	**1**	**2**	**3**	**4**	**5**	**6-10**	**11-50**	**51-100**	**101-1278**	**All**
January	3,238	663	312	174	131	299	359	48	49	5,273
February	1,595	371	157	94	81	168	231	37	53	2,787
March	2,499	555	305	138	104	242	264	41	48	4,196
April	2,629	633	275	152	116	254	304	65	57	4,485
May	3,902	789	319	186	126	296	342	66	51	6,077
June	1,148	237	115	60	36	90	121	31	43	1,881
July	5,140	965	388	233	122	309	229	39	34	7,459
August	6,945	1,534	624	332	220	513	379	47	31	10,625
September	3,256	643	298	194	121	259	270	36	25	5,102
October	5,126	1,087	482	256	161	428	447	101	72	8,160
November	12,843	2,230	935	504	338	928	868	143	83	18,872
December	11,490	2,021	874	480	289	800	744	115	95	16,908
Total	59,811	11,728	5,084	2,803	1,845	4,586	4,558	769	641	91,825

Table D1. Identical sequence clusters from Denmark.

	Cluster size									
**Month**	**1**	**2**	**3**	**4**	**5**	**6-10**	**11-50**	**51-100**	**101-1278**	**All**
January	0.6141	0.1257	0.0592	0.0330	0.0248	0.0567	0.0681	0.0091	0.0093	1
February	0.5723	0.1331	0.0563	0.0337	0.0291	0.0603	0.0829	0.0133	0.0190	1
March	0.5956	0.1323	0.0727	0.0329	0.0248	0.0577	0.0629	0.0098	0.0114	1
April	0.5862	0.1411	0.0613	0.0339	0.0259	0.0566	0.0678	0.0145	0.0127	1
May	0.6421	0.1298	0.0525	0.0306	0.0207	0.0487	0.0563	0.0109	0.0084	1
June	0.6103	0.1260	0.0611	0.0319	0.0191	0.0478	0.0643	0.0165	0.0229	1
July	0.6891	0.1294	0.0520	0.0312	0.0164	0.0414	0.0307	0.0052	0.0046	1
August	0.6536	0.1444	0.0587	0.0312	0.0207	0.0483	0.0357	0.0044	0.0029	1
September	0.6382	0.1260	0.0584	0.0380	0.0237	0.0508	0.0529	0.0071	0.0049	1
October	0.6282	0.1332	0.0591	0.0314	0.0197	0.0525	0.0548	0.0124	0.0088	1
November	0.6805	0.1182	0.0495	0.0267	0.0179	0.0492	0.0460	0.0076	0.0044	1
December	0.6796	0.1195	0.0517	0.0284	0.0171	0.0473	0.0440	0.0068	0.0056	1
Total	0.6514	0.1277	0.0554	0.0305	0.0201	0.0499	0.0496	0.0084	0.0070	1

Table D2. Identical sequence clusters from Denmark (proportion of clusters).

A.3 Germany

Month	Nocc	Nos	Nosic	Noc	Nos/Nocc	Solc
January	482,531	6,860	10,872	5,024	0.0142	176
February	224,866	18,287	28,013	11,870	0.0813	183
March	373,570	34,372	49,996	20,202	0.0920	226
April	567,343	41,564	59,711	25,764	0.0733	226
May	294,982	26,786	42,130	18,234	0.0908	226
June	48,307	8,155	13,741	5,668	0.1688	283
July	43,089	9,001	12,960	5,687	0.2089	283
August	178,357	29,427	39,608	20,224	0.1650	283
September	284,190	40,701	54,270	27,844	0.1432	283
October	377,348	31,376	46,176	21,753	0.0831	283
November	1,255,833	47,177	62,969	34,850	0.0376	283
December	1,299,987	51,047	71,458	38,833	0.0393	800
Total	5,430,403	344,753	491,904	235,953	0.0635	800

Table E. Overview of data from Germany.
Nocc: Number of confirmed cases Nos: Number of sampled sequences Nosic: Number of sequences in clusters Noc: Number of clusters Solc: Size of largest clusters.

	Cluster size									
**Month**	**1**	**2**	**3**	**4**	**5**	**6-10**	**11-50**	**51-100**	**101-800**	**All**
January	3,779	554	215	129	88	142	102	12	3	5,024
February	8,568	1,418	579	312	223	405	331	27	7	11,870
March	14,022	2,557	1,077	604	400	879	610	42	11	20,202
April	17,982	3,301	1,420	795	503	1,050	665	37	11	25,764
May	12,747	2,405	1,015	519	352	698	464	24	10	18,234
June	3,925	784	302	164	120	225	128	16	4	5,668
July	4,060	772	274	144	94	196	134	8	5	5,687
August	15,006	2,527	987	464	315	551	348	17	9	20,224
September	20,418	3,617	1,353	716	459	781	468	23	9	27,844
October	15,817	2,667	1,029	571	391	770	475	25	8	21,753
November	26,384	4,405	1,465	783	469	846	466	24	8	34,850
December	30,802	4,163	1,432	740	398	735	503	38	22	38,833
Total	173,510	29,170	11,148	5,941	3,812	7,278	4,694	293	107	235,953

Table F1. Identical sequence clusters from Germany.

	Cluster size									
**Month**	**1**	**2**	**3**	**4**	**5**	**6-10**	**11-50**	**51-100**	**101-800**	**All**
January	0.7522	0.1103	0.0428	0.0257	0.0175	0.0283	0.0203	0.0024	0.0006	1
February	0.7218	0.1195	0.0488	0.0263	0.0188	0.0341	0.0279	0.0023	0.0006	1
March	0.6941	0.1266	0.0533	0.0299	0.0198	0.0435	0.0302	0.0021	0.0005	1
April	0.6980	0.1281	0.0551	0.0309	0.0195	0.0408	0.0258	0.0014	0.0004	1
May	0.6991	0.1319	0.0557	0.0285	0.0193	0.0383	0.0254	0.0013	0.0005	1
June	0.6925	0.1383	0.0533	0.0289	0.0212	0.0397	0.0226	0.0028	0.0007	1
July	0.7139	0.1357	0.0482	0.0253	0.0165	0.0345	0.0236	0.0014	0.0009	1
August	0.7420	0.1250	0.0488	0.0229	0.0156	0.0272	0.0172	0.0008	0.0004	1
September	0.7333	0.1299	0.0486	0.0257	0.0165	0.0280	0.0168	0.0008	0.0003	1
October	0.7271	0.1226	0.0473	0.0262	0.0180	0.0354	0.0218	0.0011	0.0004	1
November	0.7571	0.1264	0.0420	0.0225	0.0135	0.0243	0.0134	0.0007	0.0002	1
December	0.7932	0.1072	0.0369	0.0191	0.0102	0.0189	0.0130	0.0010	0.0006	1
Total	0.7354	0.1236	0.0472	0.0252	0.0162	0.0308	0.0199	0.0012	0.0005	1

Table F2. Identical sequence clusters from Germany (proportion of clusters).

B Probability Theory

B.1 Transmission within identical sequence clusters

In this chapter, we present a detailed mathematical derivation of a statement upon which our model of the size distribution of identical sequence clusters is built: If the number of secondary cases created by an infected individual follows a negative binomial distribution with mean R_e and dispersion parameter k, then the number of secondary cases that belong to the same identical sequence cluster as their source case also follows a negative binomial distribution with mean $R_g = (1-\mu)R_e$, where μ is the probability of a mutation occurring in an infected individual, and dispersion parameter k.

First, we present this result in a mathematical context. Its application within our model of the size distribution of identical sequence clusters will be explained in the next chapter. We start by proofing two auxiliary results that we will use within the proof of our main statement.

Lemma 1. *For all $R, k \in \mathbb{R}_{>0}$, for all $\mu \in [0,1]$ and for all $j \in \mathbb{Z}_{\geq 0}$:*

$$\sum_{l=0}^{\infty} \frac{\Gamma(k+j+l)}{l!\Gamma(k+j)} \left(\frac{\mu R}{k+R}\right)^l = \left(\frac{k+R}{k+(1-\mu)R}\right)^{k+j}.$$

Proof. We define

$$f_j(x) = x^{-(k+j)}.$$

By Taylor's theorem:

$$\begin{aligned}
\left(\frac{k+R}{k+(1-\mu)R}\right)^{k+j} &= f_j\left(\frac{k+(1-\mu)R}{k+R}\right) \\
&= f_j(1) + \sum_{l=1}^{\infty} \frac{f_j^{(l)}(1)}{l!} \left(\frac{k+(1-\mu)R}{k+R} - 1\right)^l \\
&= 1 + \sum_{l=1}^{\infty} \frac{\prod_{m=0}^{l-1} -(k+j+m)}{l!} \left(-\frac{\mu R}{k+R}\right)^l \\
&= 1 + \sum_{l=1}^{\infty} \frac{\prod_{m=0}^{l-1} (k+j+m)}{l!} \left(\frac{\mu R}{k+R}\right)^l \\
&= \sum_{l=0}^{\infty} \frac{\Gamma(k+j+l)}{l!\Gamma(k+j)} \left(\frac{\mu R}{k+R}\right)^l
\end{aligned}$$

□

Lemma 2. *For all $R, k \in \mathbb{R}_{>0}$, for all $\mu \in [0,1]$ and for all $j \in \mathbb{Z}_{\geq 0}$:*

$$\sum_{i=j}^{\infty} \frac{\Gamma(k+i)}{(i-j)!\Gamma(k+j)} (\mu R)^{i-j} (k+(1-\mu)R)^{k+j} (k+R)^{-(k+i)} = 1$$

Proof. It holds:

$$\begin{aligned}
&\sum_{i=j}^{\infty} \frac{\Gamma(k+i)}{(i-j)!\Gamma(k+j)} (\mu R)^{i-j} (k+(1-\mu)R)^{k+j} (k+R)^{-(k+i)} \\
&= \left(\frac{k+(1-\mu)R}{k+R}\right)^{k} \sum_{i=j}^{\infty} \frac{\Gamma(k+i)}{(i-j)!\Gamma(k+j)} (\mu R)^{i-j} (k+(1-\mu)R)^{j} (k+R)^{-i} \\
&= \left(\frac{k+(1-\mu)R}{k+R}\right)^{k} \sum_{l=0}^{\infty} \frac{\Gamma(k+j+l)}{l!\Gamma(k+j)} (\mu R)^{l} (k+(1-\mu)R)^{j} (k+R)^{-(j+l)} \\
&= \left(\frac{k+(1-\mu)R}{k+R}\right)^{k} \sum_{l=0}^{\infty} \frac{\Gamma(k+j+l)}{l!\Gamma(k+j)} \left(\frac{k+(1-\mu)R}{k+R}\right)^{j} \left(\frac{\mu R}{k+R}\right)^{l} \\
&= \left(\frac{k+(1-\mu)R}{k+R}\right)^{k+j} \sum_{l=0}^{\infty} \frac{\Gamma(k+j+l)}{l!\Gamma(k+j)} \left(\frac{\mu R}{k+R}\right)^{l}
\end{aligned}$$

We conclude by applying Lemma 1. □

Next, we turn to the proof of the main statement of this chapter.

Theorem 1. *Let $R, k \in \mathbb{R}_{>0}$ and let $\mu \in [0,1]$. Furthermore, let X be an integer-valued random variable following a negative binomial distribution with mean R and dispersion parameter k, i.e.,*

$$\mathbb{P}\left(X=i\right) = \frac{\Gamma\left(k+i\right)}{i!\Gamma\left(k\right)}\left(\frac{k}{k+R}\right)^{k}\left(\frac{R}{k+R}\right)^{i} \quad \textit{for all}\, i \in \mathbb{Z}_{\geq 0}.$$

The discrete random variable Y with

$$\mathbb{P}\left(Y=j\right) = \sum_{i=j}^{\infty}\binom{i}{j}\left(1-\mu\right)^{j}\mu^{i-j}\,\mathbb{P}\left(X=i\right) \quad \textit{for all}\, j \in \mathbb{Z}_{\geq 0}$$

follows a negative binomial distribution with mean $\left(1-\mu\right)R$ and dispersion parameter k.

Proof. For all $j \in \mathbb{Z}_{\geq 0}$ we get:

$$\begin{aligned}
\mathbb{P}\left(Y=j\right) &= \sum_{i=j}^{\infty}\binom{i}{j}\left(1-\mu\right)^{j}\mu^{i-j}\,\mathbb{P}\left(X=i\right)\\
&= \sum_{i=j}^{\infty}\binom{i}{j}\left(1-\mu\right)^{j}\mu^{i-j}\frac{\Gamma\left(k+i\right)}{i!\Gamma\left(k\right)}\left(\frac{k}{k+R}\right)^{k}\left(\frac{R}{k+R}\right)^{i}\\
&= \left(\frac{k}{k+\left(1-\mu\right)R}\right)^{k}\sum_{i=j}^{\infty}\binom{i}{j}\left(1-\mu\right)^{j}\mu^{i-j}\frac{\Gamma\left(k+i\right)}{i!\Gamma\left(k\right)}\left(\frac{k}{k+R}\right)^{k}\left(\frac{k+\left(1-\mu\right)R}{k}\right)^{k}\left(\frac{R}{k+R}\right)^{i}\\
&= \left(\frac{k}{k+\left(1-\mu\right)R}\right)^{k}\sum_{i=j}^{\infty}\binom{i}{j}\left(1-\mu\right)^{j}\mu^{i-j}\frac{\Gamma\left(k+i\right)}{i!\Gamma\left(k\right)}\left(\frac{k+\left(1-\mu\right)R}{k+R}\right)^{k}\left(\frac{R}{k+R}\right)^{i}\\
&= \frac{\Gamma\left(k+j\right)}{j!\Gamma\left(k\right)}\left(\frac{k}{k+\left(1-\mu\right)R}\right)^{k}\sum_{i=j}^{\infty}\left(1-\mu\right)^{j}\mu^{i-j}\frac{\Gamma\left(k+i\right)}{\left(i-j\right)!\Gamma\left(k+j\right)}\left(\frac{k+\left(1-\mu\right)R}{k+R}\right)^{k}\left(\frac{R}{k+R}\right)^{i}
\end{aligned}$$

Furthermore,

$$\begin{aligned}
&\sum_{i=j}^{\infty}\left(1-\mu\right)^{j}\mu^{i-j}\frac{\Gamma\left(k+i\right)}{\left(i-j\right)!\Gamma\left(k+j\right)}\left(\frac{k+\left(1-\mu\right)R}{k+R}\right)^{k}\left(\frac{R}{k+R}\right)^{i}\\
&= \left(\frac{\left(1-\mu\right)R}{k+\left(1-\mu\right)R}\right)^{j}\sum_{i=j}^{\infty}\mu^{i-j}\frac{\Gamma\left(k+i\right)}{\left(i-j\right)!\Gamma\left(k+j\right)}\left(\frac{k+\left(1-\mu\right)R}{k+R}\right)^{k}\left(\frac{k+\left(1-\mu\right)R}{R}\right)^{j}\left(\frac{R}{k+R}\right)^{i}\\
&= \left(\frac{\left(1-\mu\right)R}{k+\left(1-\mu\right)R}\right)^{j}\sum_{i=j}^{\infty}\frac{\Gamma\left(k+i\right)}{\left(i-j\right)!\Gamma\left(k+j\right)}\left(\mu R\right)^{i-j}\left(k+\left(1-\mu\right)R\right)^{k+j}\left(k+R\right)^{-\left(k+i\right)}
\end{aligned}$$

By Lemma 2, we get

$$\mathbb{P}\left(Y=j\right) = \frac{\Gamma\left(k+j\right)}{j!\Gamma\left(k\right)}\left(\frac{k}{k+\left(1-\mu\right)R}\right)^{k}\left(\frac{\left(1-\mu\right)R}{k+\left(1-\mu\right)R}\right)^{j}$$

□

B.2 Sum of independent negative binomial random variables

The statements in this chapter present the requirements for the sum of two independent negative binomial random variables to follow a negative binomial distribution. We start with an auxiliary lemma that we will need for the proof of Proposition 1.

Lemma 3. *Let $N \in \mathbb{Z}_{\geq 0}$ and let $r_1, r_2 \in \mathbb{R}_{>0}$. Then*

$$\frac{\Gamma\left(N + r_1 + r_2\right)}{N!\,\Gamma\left(r_1 + r_2\right)} = \sum_{n=0}^{N} \frac{\Gamma\left(n + r_1\right)}{n!\,\Gamma\left(r_1\right)} \frac{\Gamma\left(N - n + r_2\right)}{(N-n)!\,\Gamma\left(r_2\right)}.$$

Proof. We prove this statement by induction over N.

(i) $N = 0$:

Let $r_1, r_2 \in \mathbb{R}_{>0}$. It holds:

$$\frac{\Gamma\left(N + r_1 + r_2\right)}{N!\,\Gamma\left(r_1 + r_2\right)} = \frac{\Gamma\left(r_1 + r_2\right)}{\Gamma\left(r_1 + r_2\right)} = 1 = \frac{\Gamma\left(r_1\right)}{\Gamma\left(r_1\right)} \frac{\Gamma\left(r_2\right)}{\Gamma\left(r_2\right)} = \sum_{n=0}^{N} \frac{\Gamma\left(n + r_1\right)}{n!\,\Gamma\left(r_1\right)} \frac{\Gamma\left(N - n + r_2\right)}{(N-n)!\,\Gamma\left(r_2\right)}.$$

(ii) $N \in \mathbb{Z}_{>0}$: We assume that the statement holds for $N - 1$ and all $r_1, r_2 \in \mathbb{R}_{>0}$.

Let $r_1, r_2 \in \mathbb{R}_{>0}$. It holds:

$$\begin{aligned}
\frac{\Gamma\left(N + r_1 + r_2\right)}{N!\,\Gamma\left(r_1 + r_2\right)} &= \frac{N - 1 + r_1 + r_2}{N} \frac{\Gamma\left(N - 1 + r_1 + r_2\right)}{(N-1)!\,\Gamma\left(r_1 + r_2\right)} \\
&= \frac{N - 1 + r_1 + r_2}{N} \sum_{n=0}^{N-1} \frac{\Gamma\left(n + r_1\right)}{n!\,\Gamma\left(r_1\right)} \frac{\Gamma\left(N - 1 - n + r_2\right)}{(N-1-n)!\,\Gamma\left(r_2\right)} \\
&= \sum_{n=0}^{N-1} \frac{(n + r_1) + (N - 1 - n + r_2)}{N} \frac{\Gamma\left(n + r_1\right)}{n!\,\Gamma\left(r_1\right)} \frac{\Gamma\left(N - 1 - n + r_2\right)}{(N-1-n)!\,\Gamma\left(r_2\right)} \\
&= \frac{1}{N} \sum_{n=0}^{N-1} \left(\frac{\Gamma\left(n + 1 + r_1\right)}{n!\,\Gamma\left(r_1\right)} \frac{\Gamma\left(N - 1 - n + r_2\right)}{(N-1-n)!\,\Gamma\left(r_2\right)} + \frac{\Gamma\left(n + r_1\right)}{n!\,\Gamma\left(r_1\right)} \frac{\Gamma\left(N - n + r_2\right)}{(N-1-n)!\,\Gamma\left(r_2\right)} \right)
\end{aligned}$$

Furthermore,

$$\begin{aligned}
\sum_{n=0}^{N-1} \frac{\Gamma\left(n + 1 + r_1\right)}{n!\,\Gamma\left(r_1\right)} \frac{\Gamma\left(N - 1 - n + r_2\right)}{(N-1-n)!\,\Gamma\left(r_2\right)} &= \sum_{n=1}^{N} \frac{\Gamma\left(n + r_1\right)}{(n-1)!\,\Gamma\left(r_1\right)} \frac{\Gamma\left(N - n + r_2\right)}{(N-n)!\,\Gamma\left(r_2\right)} \\
&= \sum_{n=0}^{N} n \frac{\Gamma\left(n + r_1\right)}{n!\,\Gamma\left(r_1\right)} \frac{\Gamma\left(N - n + r_2\right)}{(N-n)!\,\Gamma\left(r_2\right)}
\end{aligned}$$

and

$$\sum_{n=0}^{N-1} \frac{\Gamma\left(n + r_1\right)}{n!\,\Gamma\left(r_1\right)} \frac{\Gamma\left(N - n + r_2\right)}{(N-1-n)!\,\Gamma\left(r_2\right)} = \sum_{n=0}^{N} (N - n) \frac{\Gamma\left(n + r_1\right)}{n!\,\Gamma\left(r_1\right)} \frac{\Gamma\left(N - n + r_2\right)}{(N-n)!\,\Gamma\left(r_2\right)}.$$

Therefore,

$$\begin{aligned}
\frac{\Gamma\left(N + r_1 + r_2\right)}{N!\,\Gamma\left(r_1 + r_2\right)} &= \frac{1}{N} \sum_{n=0}^{N} \big(n + (N - n)\big) \frac{\Gamma\left(n + r_1\right)}{n!\,\Gamma\left(r_1\right)} \frac{\Gamma\left(N - n + r_2\right)}{(N-n)!\,\Gamma\left(r_2\right)} \\
&= \sum_{n=0}^{N} \frac{\Gamma\left(n + r_1\right)}{n!\,\Gamma\left(r_1\right)} \frac{\Gamma\left(N - n + r_2\right)}{(N-n)!\,\Gamma\left(r_2\right)}.
\end{aligned}$$

□

In the following statements, we use a different notation for the parameterization of a negative binomial random variable. We describe this briefly in the following remark.

Remark 1. *There are multiple ways to parameterize a negative-binomially distributed random variable X. So far, we have used the mean $m \geq 0$ (sometimes denoted by R_e) and the dispersion parameter $k > 0$, i.e.,*

$$\mathbb{P}\left(X = j\right) = \frac{\Gamma\left(k + j\right)}{j!\,\Gamma\left(k\right)}\Big(\frac{k}{k + m}\Big)^{k}\Big(\frac{m}{k + m}\Big)^{j} \quad \text{for all } j \in \mathbb{Z}_{\geq 0}.$$

Alternatively, we can use the number of successes until the experiment is stopped $r > 0$ and the success probability $p \in [0, 1]$, i.e.,

$$\mathbb{P}\left(X = j\right) = \frac{\Gamma\left(r + j\right)}{j!\,\Gamma\left(r\right)}\left(1 - p\right)^{j} p^{r} \quad \text{for all } j \in \mathbb{Z}_{\geq 0}.$$

The connections between these setups are as follows:

(i) $m = \frac{r(1-p)}{p}$ *and* $k = r$

(ii) $r = k$ *and* $p = \frac{r}{m+r}$

Next, we prove that the sum of two independent negative binomial random variables with the same success probability follows a negative binomial distribution.

Proposition 1. *The sum of two independent negative-binomially distributed random variables V_1 and V_2 with parameters*

$$r_1,\ p \quad \text{and} \quad r_2,\ p, \quad \text{where}\, r_1, r_2 > 0 \text{ and}\, p \in \left(0, 1\right),\ \text{ i.e.,}$$

$$\mathbb{P}\left(V_1 = j\right) = \frac{\Gamma\left(r_1 + j\right)}{j!\,\Gamma\left(r_1\right)}\left(1 - p\right)^{j} p^{r_1} \text{ and } \mathbb{P}\left(V_2 = j\right) = \frac{\Gamma\left(r_2 + j\right)}{j!\,\Gamma\left(r_2\right)}\left(1 - p\right)^{j} p^{r_2} \quad \text{for all } j \in \mathbb{Z}_{\geq 0},$$

is a negative-binomially distributed random variable with parameters $r = r_1 + r_2$ and p.

Proof. We denote the moment generating functions of V_1 and V_2 by $M_{V_1}\left(t\right)$ and $M_{V_2}\left(t\right)$. It holds:

$$\begin{aligned}
M_{V_1}\left(t\right) M_{V_2}\left(t\right) &= \left(\sum_{j=0}^{\infty} e^{jt}\frac{\Gamma\left(j + r_1\right)}{j!\,\Gamma\left(r_1\right)}\left(1 - p\right)^{j} p^{r_1}\right)\left(\sum_{l=0}^{\infty} e^{lt}\frac{\Gamma\left(l + r_2\right)}{l!\,\Gamma\left(r_2\right)}\left(1 - p\right)^{l} p^{r_2}\right)\\
&= \left(\sum_{j=0}^{\infty} \frac{\Gamma\left(j + r_1\right)}{j!\,\Gamma\left(r_1\right)}\left(e^{t}\left(1 - p\right)\right)^{j}\right)\left(\sum_{l=0}^{\infty} \frac{\Gamma\left(l + r_2\right)}{l!\,\Gamma\left(r_2\right)}\left(e^{t}\left(1 - p\right)\right)^{l}\right) p^{r_1 + r_2}\\
&= \sum_{N=0}^{\infty}\sum_{n=0}^{N} \frac{\Gamma\left(n + r_1\right)}{n!\,\Gamma\left(r_1\right)}\left(e^{t}\left(1 - p\right)\right)^{n} \frac{\Gamma\left(N - n + r_2\right)}{\left(N - n\right)!\,\Gamma\left(r_2\right)}\left(e^{t}\left(1 - p\right)\right)^{N-n} p^{r_1 + r_2}\\
&= \sum_{N=0}^{\infty}\left(e^{t}\left(1 - p\right)\right)^{N} p^{r_1 + r_2}\sum_{n=0}^{N} \frac{\Gamma\left(n + r_1\right)}{n!\,\Gamma\left(r_1\right)}\frac{\Gamma\left(N - n + r_2\right)}{\left(N - n\right)!\,\Gamma\left(r_2\right)}.
\end{aligned}$$

By Lemma 3,

$$M_{V_1}\left(t\right) M_{V_2}\left(t\right) = \sum_{N=0}^{\infty} e^{Nt}\frac{\Gamma\left(N + r_1 + r_2\right)}{N!\,\Gamma\left(r_1 + r_2\right)}\left(1 - p\right)^{N} p^{r_1 + r_2}.$$

Since V_1 and V_2 are independent,

$$M_{V_1 + V_2}\left(t\right) = M_{V_1}\left(t\right) M_{V_2}\left(t\right) = \sum_{N=0}^{\infty} e^{Nt}\frac{\Gamma\left(N + r_1 + r_2\right)}{N!\,\Gamma\left(r_1 + r_2\right)}\left(1 - p\right)^{N} p^{r_1 + r_2}.$$

Since $M_{V_1+V_2}\left(t\right)$ has the form of the moment generating function of a negative binomial distribution with parameters $r_1 + r_2$ and p,

$$V_1 + V_2 \text{ is a negative-binomially distributed random variable with parameters } r_1 + r_2 \text{ and } p.$$

□

Finally, we show that for the sum of two independent negative binomial random variables to follow a negative binomial distribution, both negative binomial random variables having the same success probability is a necessary condition.

Theorem 2. *The sum of two independent negative-binomially distributed random variables V_1 and V_2 with parameters*

$$r_1,\ p_1 \quad \textit{and} \quad r_2,\ p_2, \quad \textit{where}\, r_1, r_2 > 0 \,\textit{and}\, p_1, p_2 \in (0,1)\,,\ \textit{i.e.,}$$

$$\mathbb{P}\left(V_1 = j\right) = \frac{\Gamma\left(r_1 + j\right)}{j!\,\Gamma\left(r_1\right)}\left(1 - p_1\right)^j p_1^{r_1} \ \textit{and}\ \mathbb{P}\left(V_2 = j\right) = \frac{\Gamma\left(r_2 + j\right)}{j!\,\Gamma\left(r_2\right)}\left(1 - p_2\right)^j p_2^{r_2} \quad \textit{for all}\ j \in \mathbb{Z}_{\geq 0},$$

is a negative-binomially distributed random variable if and only if $p_1 = p_2$.

Proof. (i) We assume that $p_1 = p_2 = p$. By Proposition 1,

$$V_1 + V_2 \text{ follows a negative binomial distribution.}$$

(ii) We assume that $V_1 + V_2$ follows a negative binomial distribution with parameters $r > 0$ and $p \in [0,1]$.

Since V_1 and V_2 are independent,

$$\mathbb{P}\left(V_1 + V_2 = 0\right) = \mathbb{P}\left(V_1 = 0 \cap V_2 = 0\right) = \mathbb{P}\left(V_1 = 0\right)\mathbb{P}\left(V_2 = 0\right).$$

Because of this and since $p_1, p_2 \in (0,1)$:

$$\mathbb{P}\left(V_1 + V_2 = 0\right) = p^r = p_1^{r_1} p_2^{r_2} \notin \{0,1\}\,,$$

hence $p \in (0,1)$.

We denote by $m_1,\ m_2$ and m the expected values of $V_1,\ V_2$ and $V_1 + V_2$. It holds:

$$\frac{r\left(1 - p\right)}{p} = m = m_1 + m_2 = \frac{r_1\left(1 - p_1\right)}{p_1} + \frac{r_2\left(1 - p_2\right)}{p_2}.$$

Furthermore, since $r, r_1, r_2 > 0$ and $p, p_1, p_2 \in (0,1)$:

$$m, m_1, m_2 > 0.$$

We define $\mu = \frac{m_2}{m}$. It follows:

$$m_1 = \left(1 - \mu\right)m \quad \text{and} \quad m_2 = \mu m.$$

Since V_1 and V_2 are independent,

$$m + \frac{m^2}{r} = \operatorname{Var}\left(V_1 + V_2\right) = \operatorname{Var}\left(V_1\right) + \operatorname{Var}\left(V_2\right) = m_1 + \frac{m_1^2}{r_1} + m_2 + \frac{m_2^2}{r_2},$$

hence

$$m + \frac{m^2}{r} = \left(1 - \mu\right)m + \frac{\left(\left(1 - \mu\right)m\right)^2}{r_1} + \mu\, m + \frac{\left(\mu\, m\right)^2}{r_2} = m + \frac{m^2\left(\mu^2 r_1 + \left(1 - \mu\right)^2 r_2\right)}{r_1 r_2}.$$

This implies that

$$m^2\left(\frac{1}{r}-\frac{\mu^2 r_1+(1-\mu)^2 r_2}{r_1 r_2}\right)=0.$$

Since $m>0$,

$$r=\frac{r_1 r_2}{\mu^2 r_1+(1-\mu)^2 r_2}.$$

As

$$\mathbb{P}\left(V_1+V_2=0\right)=\mathbb{P}\left(V_1=0\cap V_2=0\right)=\mathbb{P}\left(V_1=0\right)\mathbb{P}\left(V_2=0\right),$$

we get

$$\left(\frac{r}{r+m}\right)^r=\left(\frac{r_1}{r_1+m_1}\right)^{r_1}\left(\frac{r_2}{r_2+m_2}\right)^{r_2}.$$

This means that

$$\left(\frac{\frac{r_1 r_2}{\mu^2 r_1+(1-\mu)^2 r_2}}{\frac{r_1 r_2}{\mu^2 r_1+(1-\mu)^2 r_2}+m}\right)^{\frac{r_1 r_2}{\mu^2 r_1+(1-\mu)^2 r_2}}=\left(\frac{r_1}{r_1+(1-\mu)\,m}\right)^{r_1}\left(\frac{r_2}{r_2+\mu m}\right)^{r_2}.$$

As

$$\begin{aligned}\mathbb{P}\left(V_1+V_2=1\right)&=\mathbb{P}\left(V_1=1\cap V_2=0\right)+\mathbb{P}\left(V_1=0\cap V_2=1\right)\\&=\mathbb{P}\left(V_1=1\right)\mathbb{P}\left(V_2=0\right)+\mathbb{P}\left(V_1=0\right)\mathbb{P}\left(V_2=1\right),\end{aligned}$$

we get

$$\begin{aligned}r\left(\frac{r}{r+m}\right)^r\frac{m}{r+m}&=r_1\left(\frac{r_1}{r_1+m_1}\right)^{r_1}\frac{m_1}{r_1+m_1}\left(\frac{r_2}{r_2+m_2}\right)^{r_2}+\left(\frac{r_1}{r_1+m_1}\right)^{r_1}r_2\left(\frac{r_2}{r_2+m_2}\right)^{r_2}\frac{m_2}{r_2+m_2}\\&=\left(\frac{r_1}{r_1+m_1}\right)^{r_1}\left(\frac{r_2}{r_2+m_2}\right)^{r_2}\left(\frac{r_1\,m_1}{r_1+m_1}+\frac{r_2\,m_2}{r_2+m_2}\right).\end{aligned}$$

This means that

$$\frac{r_1 r_2}{\mu^2 r_1+(1-\mu)^2 r_2}\left(\frac{\frac{r_1 r_2}{\mu^2 r_1+(1-\mu)^2 r_2}}{\frac{r_1 r_2}{\mu^2 r_1+(1-\mu)^2 r_2}+m}\right)^{\frac{r_1 r_2}{\mu^2 r_1+(1-\mu)^2 r_2}}\frac{m}{\frac{r_1 r_2}{\mu^2 r_1+(1-\mu)^2 r_2}+m}=$$

$$\left(\frac{r_1}{r_1+(1-\mu)\,m}\right)^{r_1}\left(\frac{r_2}{r_2+\mu m}\right)^{r_2}\left(\frac{(1-\mu)\,m r_1}{r_1+(1-\mu)\,m}+\frac{\mu m r_2}{r_2+\mu m}\right).$$

It follows that

$$\frac{r_1 r_2}{\mu^2 r_1+(1-\mu)^2 r_2}\frac{m}{\frac{r_1 r_2}{\mu^2 r_1+(1-\mu)^2 r_2}+m}=\frac{(1-\mu)\,m r_1}{r_1+(1-\mu)\,m}+\frac{\mu m r_2}{r_2+\mu m},$$

hence

$$\frac{m r_1 r_2}{r_1 r_2+m\big(\mu^2 r_1+(1-\mu)^2 r_2\big)}=\frac{(1-\mu)\,m r_1\left(r_2+\mu m\right)+\mu m r_2\big(r_1+(1-\mu)\,m\big)}{\left(r_1+(1-\mu)\,m\right)\left(r_2+\mu m\right)}.$$

Now, our goal is to express μ in dependence of r_1, r_2 and m. This will take multiple steps.

Step 1:

$$mr_1r_2\left(r_1+\left(1-\mu\right)m\right)\left(r_2+\mu m\right)=$$
$$\left(r_1r_2+m\left(\mu^2r_1+\left(1-\mu\right)^2r_2\right)\right)\left(\left(1-\mu\right)mr_1\left(r_2+\mu m\right)+\mu mr_2\left(r_1+\left(1-\mu\right)m\right)\right)$$

Step 2:

$$mr_1r_2\left(r_1r_2+\mu mr_1+\left(1-\mu\right)mr_2+\mu\left(1-\mu\right)m^2\right)=$$
$$\left(1-\mu\right)mr_1^2r_2\left(r_2+\mu m\right)+\mu mr_1r_2^2\left(r_1+\left(1-\mu\right)m\right)+$$
$$\left(1-\mu\right)m^2r_1\left(\mu^2r_1+\left(1-\mu\right)^2r_2\right)\left(r_2+\mu m\right)+\mu m^2r_2\left(\mu^2r_1+\left(1-\mu\right)^2r_2\right)\left(r_1+\left(1-\mu\right)m\right)$$

Step 3:

$$mr_1^2r_2^2+\mu m^2r_1^2r_2+\left(1-\mu\right)m^2r_1r_2^2+\mu\left(1-\mu\right)m^3r_1r_2=$$
$$\left(1-\mu\right)mr_1^2r_2^2+\mu\left(1-\mu\right)m^2r_1^2r_2+\mu mr_1^2r_2^2+\mu\left(1-\mu\right)m^2r_1r_2^2+$$
$$\mu^2\left(1-\mu\right)m^2r_1^2r_2+\mu^3\left(1-\mu\right)m^3r_1^2+\left(1-\mu\right)^3m^2r_1r_2^2+\mu\left(1-\mu\right)^3m^3r_1r_2+$$
$$\mu^3m^2r_1^2r_2+\mu^3\left(1-\mu\right)m^3r_1r_2+\mu\left(1-\mu\right)^2m^2r_1r_2^2+\mu\left(1-\mu\right)^3m^3r_2^2$$

Step 4:

$$mr_1^2r_2^2\left(1-\left(1-\mu\right)-\mu\right)+m^2r_1^2r_2\left(\mu-\mu\left(1-\mu\right)-\mu^2\left(1-\mu\right)-\mu^3\right)+$$
$$m^2r_1r_2^2\left(\left(1-\mu\right)-\mu\left(1-\mu\right)-\left(1-\mu\right)^3-\mu\left(1-\mu\right)^2\right)-$$
$$\mu^3\left(1-\mu\right)m^3r_1^2-\mu\left(1-\mu\right)^3m^3r_2^2+m^3r_1r_2\left(\mu\left(1-\mu\right)-\mu\left(1-\mu\right)^3-\mu^3\left(1-\mu\right)\right)=0$$

Step 5:

$$-\mu^3\left(1-\mu\right)m^3r_1^2-\mu\left(1-\mu\right)^3m^3r_2^2+2\mu^2\left(1-\mu\right)^2m^3r_1r_2=0$$

Step 6:

$$\mu\left(1-\mu\right)m^3\left(\mu^2r_1^2-2\mu\left(1-\mu\right)r_1r_2+\left(1-\mu\right)^2r_2^2\right)=0$$

Step 7:

$$\mu\left(1-\mu\right)\left(\mu r_1-\left(1-\mu\right)r_2\right)^2=0$$

It follows that $\mu=0$, $\mu=1$ or $\mu=\frac{r_2}{r_1+r_2}$. Since $m_1>0$ and $m_2>0$,

$$\mu=\frac{r_2}{r_1+r_2}.$$

It holds:

$$m=\frac{r_1\left(1-p_1\right)}{p_1}+\frac{r_2\left(1-p_2\right)}{p_2}=\frac{r_1\left(1-p_1\right)p_2+r_2p_1\left(1-p_2\right)}{p_1p_2}$$

Furthermore,

$$
\begin{aligned}
p_1 &= \frac{r_1}{m_1 + r_1} \\
&= \frac{r_1}{(1-\mu)\, m + r_1} \\
&= \frac{r_1}{\frac{r_1}{r_1+r_2} \frac{r_1(1-p_1)p_2 + r_2 p_1 (1-p_2)}{p_1 p_2} + r_1} \\
&= \frac{r_1 (r_1 + r_2) p_1 p_2}{r_1^2 (1-p_1) p_2 + r_1 r_2 p_1 (1-p_2) + r_1 (r_1 + r_2) p_1 p_2} \\
&= \frac{r_1 (r_1 + r_2) p_1 p_2}{r_1^2 p_2 - r_1^2 p_1 p_2 + r_1 r_2 p_1 - r_1 r_2 p_1 p_2 + r_1^2 p_1 p_2 + r_1 r_2 p_1 p_2} \\
&= \frac{(r_1 + r_2) p_1 p_2}{r_1 p_2 + r_2 p_1}.
\end{aligned}
$$

It follows that

$$r_1 p_1 p_2 + r_2 p_1^2 = r_1 p_1 p_2 + r_2 p_1 p_2.$$

Therefore,

$$r_2 p_1^2 = r_2 p_1 p_2.$$

We conclude that

$$p_1 = p_2.$$

□

As our last statement of this chapter, we rephrase Theorem 2. Instead of parameterizing a negative-binomially distributed random variable by the number of successes until the experiment is stopped r and the success probability p, we parameterize it by the mean m and the dispersion parameter k.

Corollary 1. *Let V_1 be a negative-binomially distributed random variable with mean $m_1 > 0$ and dispersion parameter $k_1 > 0$ and let V_2 be a negative-binomially distributed random variable with mean $m_2 > 0$ and dispersion parameter $k_2 > 0$. If V_1 and V_2 are independent, then $V_1 + V_2$ is a negative-binomially distributed random variable if and only if $m_1 k_2 = m_2 k_1$.*

Proof. Let p_1 be the success probability of V_1 and let p_2 be the success probability of V_2. It holds:

$$p_1 = \frac{k_1}{m_1 + k_1} \in (0,1) \quad \text{and} \quad p_2 = \frac{k_2}{m_2 + k_2} \in (0,1).$$

By Theorem 2, $V_1 + V_2$ follows a negative binomial distribution if and only if

$$\frac{k_1}{m_1 + k_1} = \frac{k_2}{m_2 + k_2}.$$

This is equivalent to

$$m_1 k_2 = m_2 k_1.$$

□

C Model of cluster size distribution

C.1 Description of model

Both for inferring the effective reproduction number R_e and the dispersion parameter k from genomic sequence data and for simulating clusters of identical sequences we built upon a classical branching process setup that we applied to viral transmission. Starting with one source case, the cases of the subsequent generation are determined by a recurrence relation. Ordering and connecting the cases according to their generation and direct predecessor, we can represent the disease outbreak as a graph, more precisely as a rooted tree. This is why in this paragraph we often use the language of graph theory, denoting cases as nodes, or all cases sharing a common direct predecessor as offspring of a node.

We start with the same assumption that Blumberg and Lloyd-Smith [5] have made: For each node, the number of offspring T follows a negative binomial distribution with mean R_e and dispersion parameter k,

$$\mathbb{P}(T = i) = \frac{\Gamma(k+i)}{i!\,\Gamma(k)} \left(\frac{k}{k+R_e}\right)^k \left(\frac{R_e}{k+R_e}\right)^i \quad \text{for all } i \in \mathbb{Z}_{\geq 0}.$$

Having set up the scaffolding of our transmission model, we move to integrating the mutation process of the virus to separate different viral clades. At each node the occurrence of a mutation is modelled by a random variable following a Bernoulli distribution with parameter μ. We assume that at each node a mutation occurs independently of all other nodes and prior to the respective node transmitting the virus to secondary cases or being detected. Therefore, if a mutation happens at a node, all direct offspring of that particular node receive the mutation. We use two different approaches to estimate μ. On the one hand, we use an estimate of μ that is based on the estimation of the mean number of mutations per transmission of Park et al. [6]. Based on 87 transmission pairs, they obtained an estimate of $M_T = 0.33$ mutations per transmission on average with a 95% confidence interval of $0.22 - 0.48$. Plugging $M_T = 0.33$ into the formula

$$\mu = 1 - \mathrm{e}^{-M_T},$$

we get $\mu = 28.1\%$. On the other hand, we determined the mutation probability μ as

$$\mu = 1 - \mathrm{e}^{-MD/365.25},$$

where M is the yearly mutation rate of SARS-CoV-2 and D the mean generation interval. Based on the estimates of $M = 14$ mutations per year obtained by Neher et al. [7] and $D = 5.2$ days obtained by Ganyani et al. [8], we obtain $\mu = 18.1\%$.

In order to determine the distribution of the size of the identical sequence clusters, we need to determine the distribution of the number of offspring V of a node that belong to the same identical sequence cluster as their parent. The number of offspring within the same identical sequence cluster of a node is the number of children of the node at which no mutation occurred, i.e., the genotype of the virus these children transmit to their offspring is identical to the genotype of the virus they received from their parent. Therefore,

$$\mathbb{P}(V = j) = \sum_{i=j}^{\infty} \binom{i}{j} (1-\mu)^j \mu^{i-j}\, \mathbb{P}(T = i) \quad \text{for all } j \in \mathbb{Z}_{\geq 0}.$$

$\mathbb{P}(V = j)$ is the probability that at j children of a node no mutation occurs (and at all other children a mutation occurs), which means that these j children belong to the same cluster as their parent. By Theorem 1, V follows a negative binomial distribution with mean $R_g = (1-\mu) R_e$, the genomic reproduction number, and dispersion parameter k, i.e.,

$$\mathbb{P}(V = j) = \frac{\Gamma(k+j)}{j!\,\Gamma(k)} \left(\frac{k}{k+R_g}\right)^k \left(\frac{R_g}{k+R_g}\right)^j \quad \text{for all } j \in \mathbb{Z}_{\geq 0}.$$

Following Blumberg and Llyod-Smith [5], we determine the probability that an identical sequence cluster has size j by

$$\mathbb{P}(X = j) = \frac{\Gamma(kj + j - 1)}{\Gamma(kj)\,\Gamma(j + 1)} \frac{\left(\frac{R_g}{k}\right)^{j-1}}{\left(1 + \frac{R_g}{k}\right)^{kj+j-1}} \quad \text{for all } j \in \mathbb{Z}_{\geq 1}.$$

As did Blumberg and Llyod-Smith [9], we model the observation of each node by a random variable following a Bernoulli distribution with parameter τ, the detection probability. The observation of each node is independent of the observation of all other nodes. The probability τ is determined as

$$\tau = \tau_{\text{test}}\ \tau_{\text{sequence}},$$

where τ_{test} is the probability that a case is confirmed by a test and τ_{sequence} is the probability that the genome of a confirmed case is sequenced. Consequently, the probability of observing j cases contained in an identical sequence cluster of size $\geq j$ is given by

$$\mathbb{P}(Y = j) = \begin{cases} \sum_{i=1}^{\infty} (1 - \tau)^{i}\ \mathbb{P}(X = i) & \text{if } j = 0, \\ \sum_{i=j}^{\infty} \binom{i}{j} \tau^{j} (1 - \tau)^{i-j}\ \mathbb{P}(X = i) & \text{if } j \in \mathbb{Z}_{\geq 1}. \end{cases}$$

When implementing our model, we approximate the above infinite sums by limiting the number of summands to a finite threshold depending on τ. For smaller values of τ, the number of summands included is larger. As identical sequence clusters of which no case is observed do not appear in the data sets, the distribution of Y does not appropriately model the size distribution of the identical sequence clusters contained in the data. To bridge this difference, we define the random variable Z, the number of observed cases in an identical sequence cluster of which at least one case has been observed. It holds:

$$\mathbb{P}(Z = j) = \frac{\mathbb{P}(Y = j)}{1 - \mathbb{P}(Y = 0)} \quad \text{for all } j \in \mathbb{Z}_{\geq 1}.$$

C.2 Additional theoretical background

Let W be the number of offspring of a node that do not belong to the same identical sequence cluster as their parent. By similar arguments as applied above to show that the random variable V follows a negative binomial distribution with mean $(1-\mu)\,R_e$ and dispersion parameter k, the random variable W follows a negative binomial distribution with mean μR_e and dispersion parameter k.

We examine whether the random variables V and W are independent.

Proposition 2. *V and W are independent if and only if $\mu = 0$ or $\mu = 1$.*

Proof. (i) If $\mu = 0$, then $V = T$ and $W = 0$. It holds for all $i,\, j \in \mathbb{Z}_{\geq 0}$:

$$\mathbb{P}\,(V = i \cap W = j) = \begin{cases} \mathbb{P}\,(V = i) = \mathbb{P}\,(V = i)\,\mathbb{P}\,(W = j) & \text{if } j = 0, \\ \mathbb{P}\,(\emptyset) = 0 = \mathbb{P}\,(V = i)\,\mathbb{P}\,(W = j) & \text{if } j \neq 0. \end{cases}$$

We conclude that V and W are independent.

(ii) If $\mu = 1$, then $V = 0$ and $W = T$. It holds for all $i,\, j \in \mathbb{Z}_{\geq 0}$:

$$\mathbb{P}\,(V = i \cap W = j) = \begin{cases} \mathbb{P}\,(W = j) = \mathbb{P}\,(V = i)\,\mathbb{P}\,(W = j) & \text{if } i = 0, \\ \mathbb{P}\,(\emptyset) = 0 = \mathbb{P}\,(V = i)\,\mathbb{P}\,(W = j) & \text{if } i \neq 0. \end{cases}$$

We conclude that V and W are independent.

(iii) Let us assume that V and W are independent. It holds:

(a) $\mathrm{Var}\,(V + W) = \mathrm{Var}\,(T) = R_e + \dfrac{R_e^2}{k}$

(b)
$$\begin{aligned}\mathrm{Var}\,(V) + \mathrm{Var}\,(W) &= (1-\mu)\,R_e + \frac{\left((1-\mu)\,R_e\right)^2}{k} + \mu\,R_e + \frac{(\mu\,R_e)^2}{k} \\ &= R_e + \frac{\left(2\mu^2 - 2\mu + 1\right)R_e^2}{k}\end{aligned}$$

Therefore, as $R_e > 0$ and $k > 0$,

$$\mathrm{Var}\,(T) = \mathrm{Var}\,(V) + \mathrm{Var}\,(W) \iff 2\mu^2 - 2\mu = 0.$$

It holds:

$$2\mu^2 - 2\mu = 0 \iff \mu\,(\mu - 1) = 0 \iff \mu = 0 \text{ or } \mu = 1.$$

□

C.3 Possible extensions of the model

If the genomic reproduction number R_g is larger than 1, then the probability that an identical sequence cluster does not go extinct is strictly bigger than 0. This means that

$$\pi = \sum_{j=0}^{\infty} \mathbb{P}\left(Z = j\right) < 1.$$

Since we can not observe identical sequence clusters of arbitrary size, this requires additional adjustments to the model in order to fully cope with this situation. We have thought about possible approaches, but have not found a satisfactory solution. In the following we outline our ideas.

Nishiura et al. [10] have shown how to compute the probability of extinction of a branching process with a negative-binomially distributed offspring distribution. Their arguments can be extended to compute π. Computing the size distribution of identical sequence clusters conditioned on them being finite is therefore possible but would require some additional computations. In our opinion, the bigger challenge lies elsewhere, namely in the classification of the potential of the clusters to grow to a large size.

Let us assume that $R_g > 1$ for a certain period. We have a number of clusters growing during that period, some already active before, some starting during this period. It is very likely that some of the clusters that have started during this period would continue to grow indefinitely if R_g would stay above 1. In reality, this does not happen. R_g will decrease below 1 at some point and all clusters will go extinct.

However, in our calculations to determine the cluster size distribution from the secondary case distribution, the effective reproduction number, the dispersion parameter, the mutation probability and the testing probability are assumed to remain constant. In order for the cluster size distribution with $R_g > 1$ derived from theory to be consistent with the observed cluster size distribution, one needs to know which clusters from the data would have grown to arbitrary size if R_g stayed above 1. Similarly, to establish consistency between the cluster size distribution derived from theory conditioned on clusters going extinct with the data, one would need to remove those clusters from the data that would not go extinct if R_g stayed above 1.

We have not managed to implement a method to separate clusters that would be likely to continue to grow if R_g stayed above 1 from those that would go extinct. Therefore, we have left this question open for the time being. However, we are convinced that this would be an exciting starting point for further development of our model.

D Prior distributions and fixed parameters

We set weakly informative prior distributions for the effective reproduction number R_e, the dispersion parameter k and the testing probability τ_{test}. Depending on the method the mutation process was included into the model, we either directly set a prior distribution for the mutation probability μ reflecting the uncertainty of the estimate obtained by Park et al. [6] or a tight prior distribution for the yearly mutation rate M, whose value was informed by work of Neher et al. [7]. We used the same prior distributions for all countries and months. For the prior distributions of R_e, k, μ and M we opted for widely used probability distributions such as the gamma, beta or normal distribution. In addition, we incorporated expert knowledge about the proportion of new cases that are confirmed by a test into the prior distribution of τ_{test}. We scaled a beta distribution, usually defined on the interval (0, 1) or [0, 1], to the interval [0.05, 1]. The reason for applying this transformation is to control the computational cost of our inference model. The lower τ_{test}, the more summands are required to reliably approximate $\mathbb{P}(Y = j)$, see chapter Model of cluster size distribution for the definition of Y. Since we are convinced that at least 5% of all COVID-19 cases were confirmed by a test at any time in 2021 in Switzerland, Denmark and Germany, we have shifted the lower limit of the domain of the prior distribution of τ_{test} from 0 to 0.05. The probability density function of the resulting probability distribution, which we denote by scaled beta distribution, is given by the following formula:

$$f(x) = \frac{(x - 0.05)^{\alpha-1}(1-x)^{\beta-1}}{(1-0.05)^{\alpha+\beta-1}} \frac{\Gamma(\alpha+\beta)}{\Gamma(\alpha)\Gamma(\beta)} \quad \text{for all } x \in [0.05,\ 1],$$

where α and β are the two shape parameters. We included the mean generation interval as a constant value [8] into our model and determined the probability that the viral genome of a confirmed case gets sequenced by dividing the monthly number of sequences by the monthly number of confirmed cases.

Tables G1 and G2 present an overview of the prior distributions as well as the fixed parameters.

Symbol	Comment	Support	Prior
R_e	Effective reproduction number	$(0, \infty)$	Gamma(shape = 10, rate = 10)
k	Dispersion parameter	$(0, \infty)$	Gamma(shape = 5, rate = 10)
μ	Mutation probability	$[0, 1]$	Beta(shape = 27, shape = 68)
M	Yearly mutation rate	$(-\infty, \infty)$	Normal(mean = 14, variance = 0.25)
τ_{test}	Testing probability	$[0.05, 1]$	ScaledBeta(shape = 1, shape = 3)

Table G1. Summary of parameters of prior distributions.

Symbol	Comment	Value	Unit	Source
D	Mean generation interval	5.2	days	Ganyani et al. 2020
τ_{sequence}	Sequencing probability	1.42 − 85.94%	percentage	see column Nos/Nocc of Tables A, C and E

Table G2. Summary of fixed parameters.

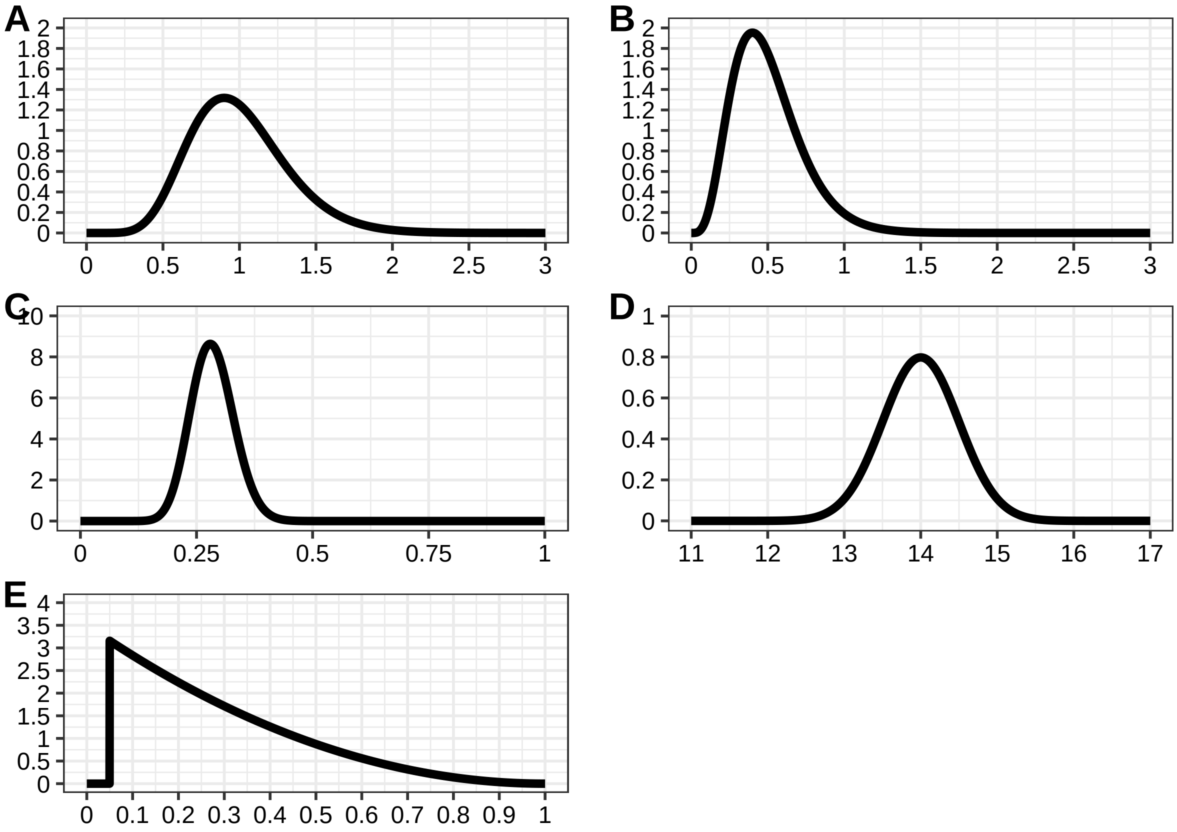

Fig A. Prior distributions. A: Prior distribution of the effective reproduction number R_e. B: Prior distribution of the dispersion parameter k. C: Prior distribution of the mutation probability μ. D: Prior distribution of the yearly mutation rate M. E: Prior distribution of the testing probability τ_{test}.

E Simulations

In this chapter, we present three alternatives to Fig 2 of the manuscript to display the results of the simulation study. We use two different methods to assess the precision of the parameter estimates. First, we use the coefficient of variation as error metric between the true value and the estimated mean of the posterior distribution. Second, we measure the error between the true value and the estimated mean of the posterior distribution applying the root mean square error (RMSE). Third, for each combination of input values we assess the proportion of repetitions of the parameter estimate in which the true value is contained in the estimated 95% credible interval of the posterior distribution.

E.1 Coefficient of variation

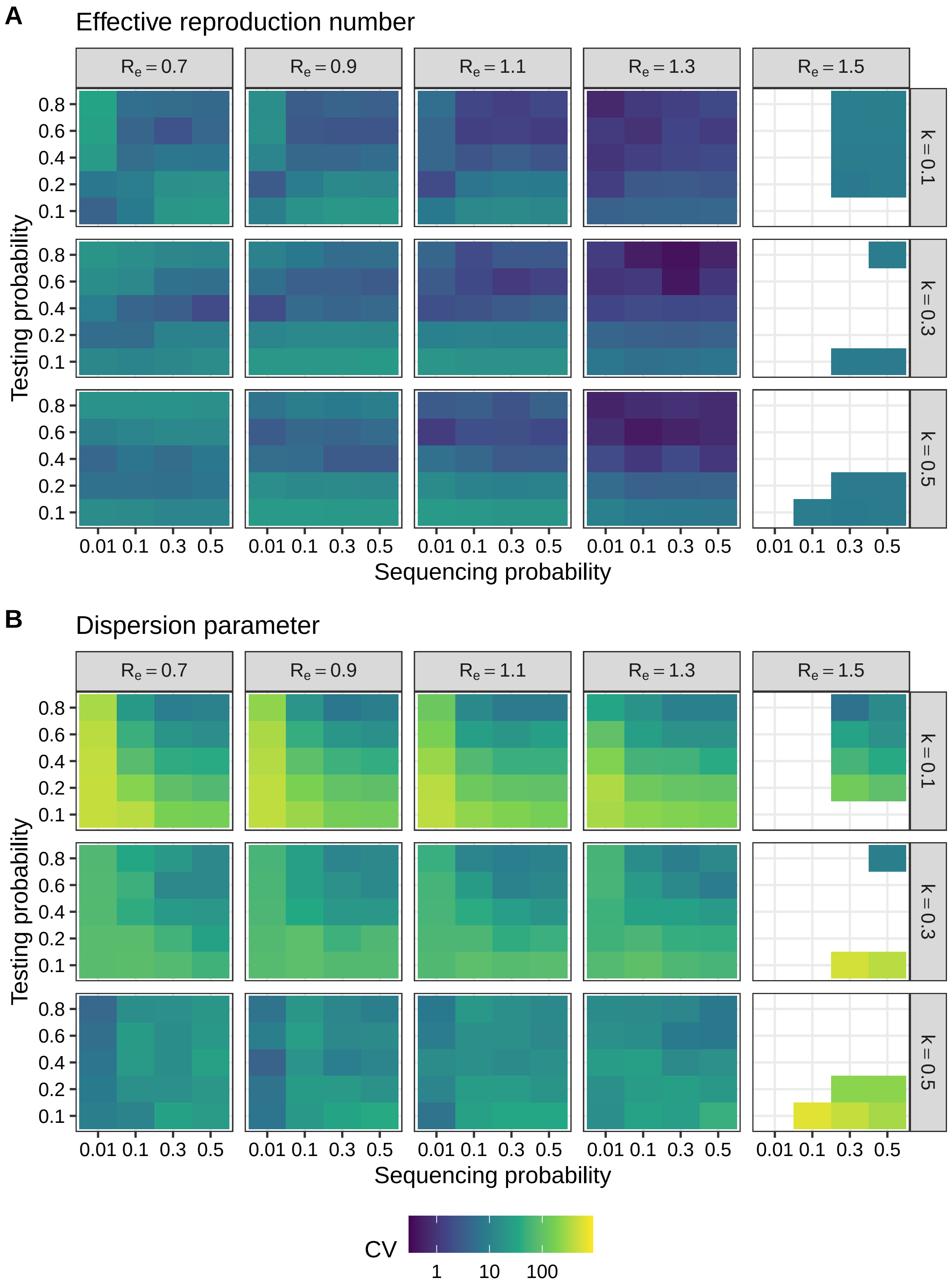

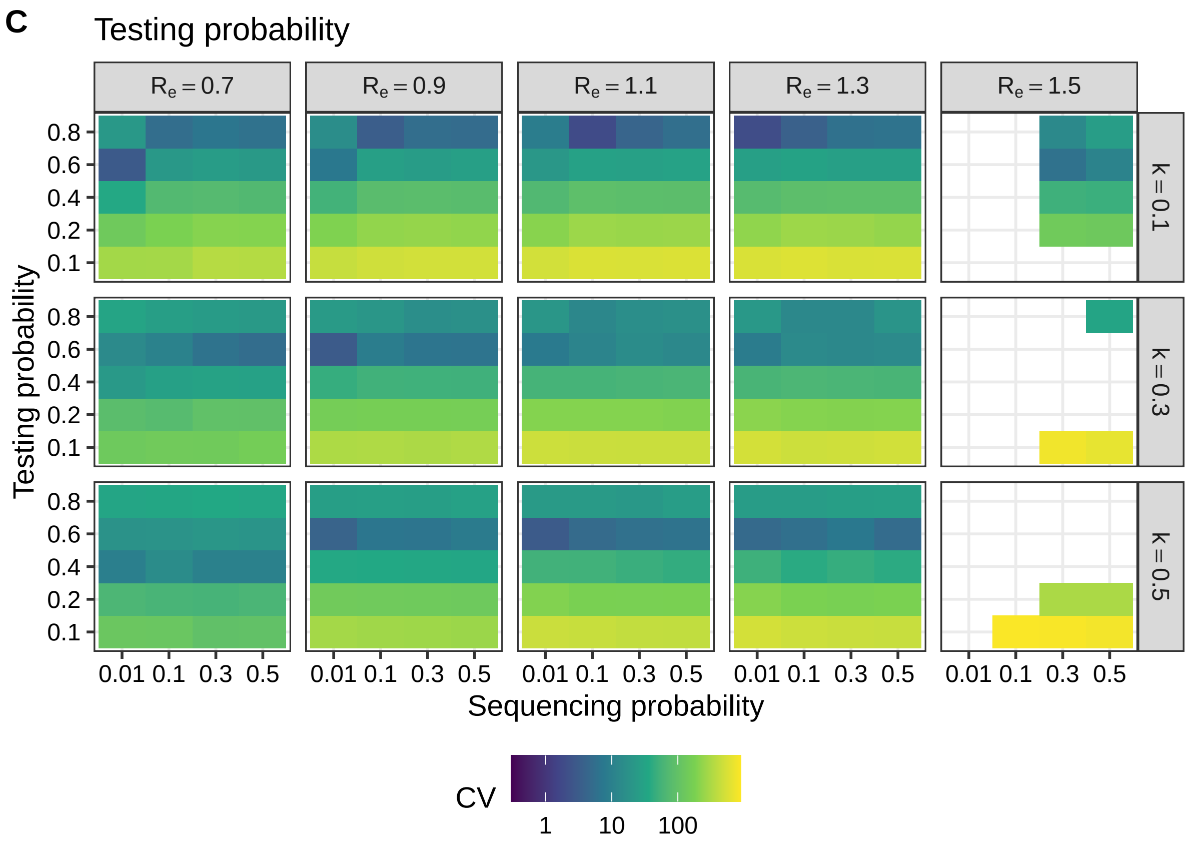

Fig B. Validation of the Bayesian inference model to estimate transmission parameters from the size distribution of identical sequence clusters. A: Coefficient of variation of the estimate of the effective reproduction number R_e. B: Coefficient of variation of the estimate of the dispersion parameter k. C: Coefficient of variation of the estimate of the testing probability τ_{test}. The coefficient of variation is computed as 100 times the ratio of the root mean squared error (RMSE) between the true value and the estimated mean of the posterior distribution to the true value. For each parameter combination, we ran the model 10 times on $3,000$ simulated clusters each.

E.2 Root mean square error

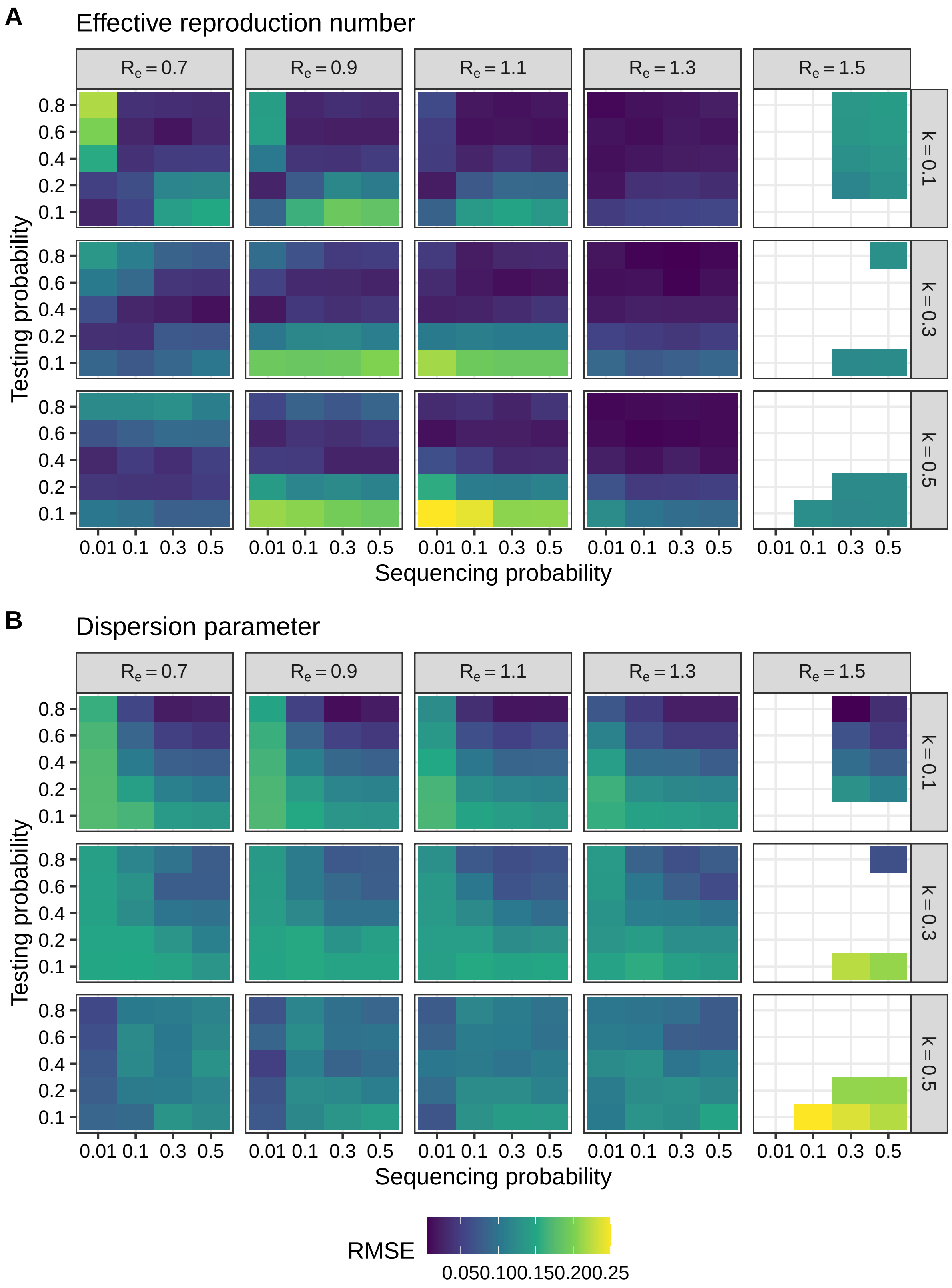

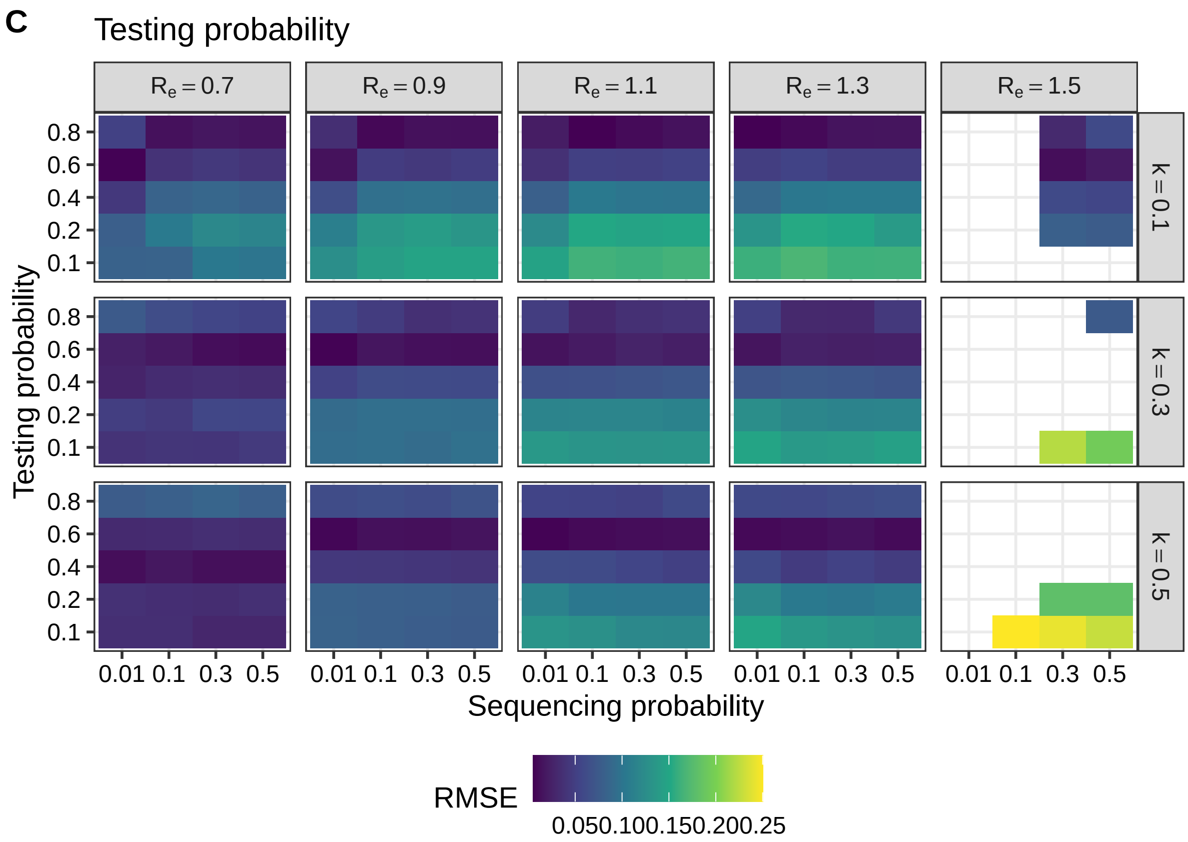

Fig C. Validation of the Bayesian inference model to estimate transmission parameters from the size distribution of identical sequence clusters. A: Error in estimating the effective reproduction number R_e. B: Error in estimating the dispersion parameter k. C: Error in estimating the testing probability τ_{test}. Error is measured by the root mean square error (RMSE) between the true value and the estimated mean of the posterior distribution. For each parameter combination, we ran the model 10 times on $3,000$ simulated clusters each.

E.3 Coverage

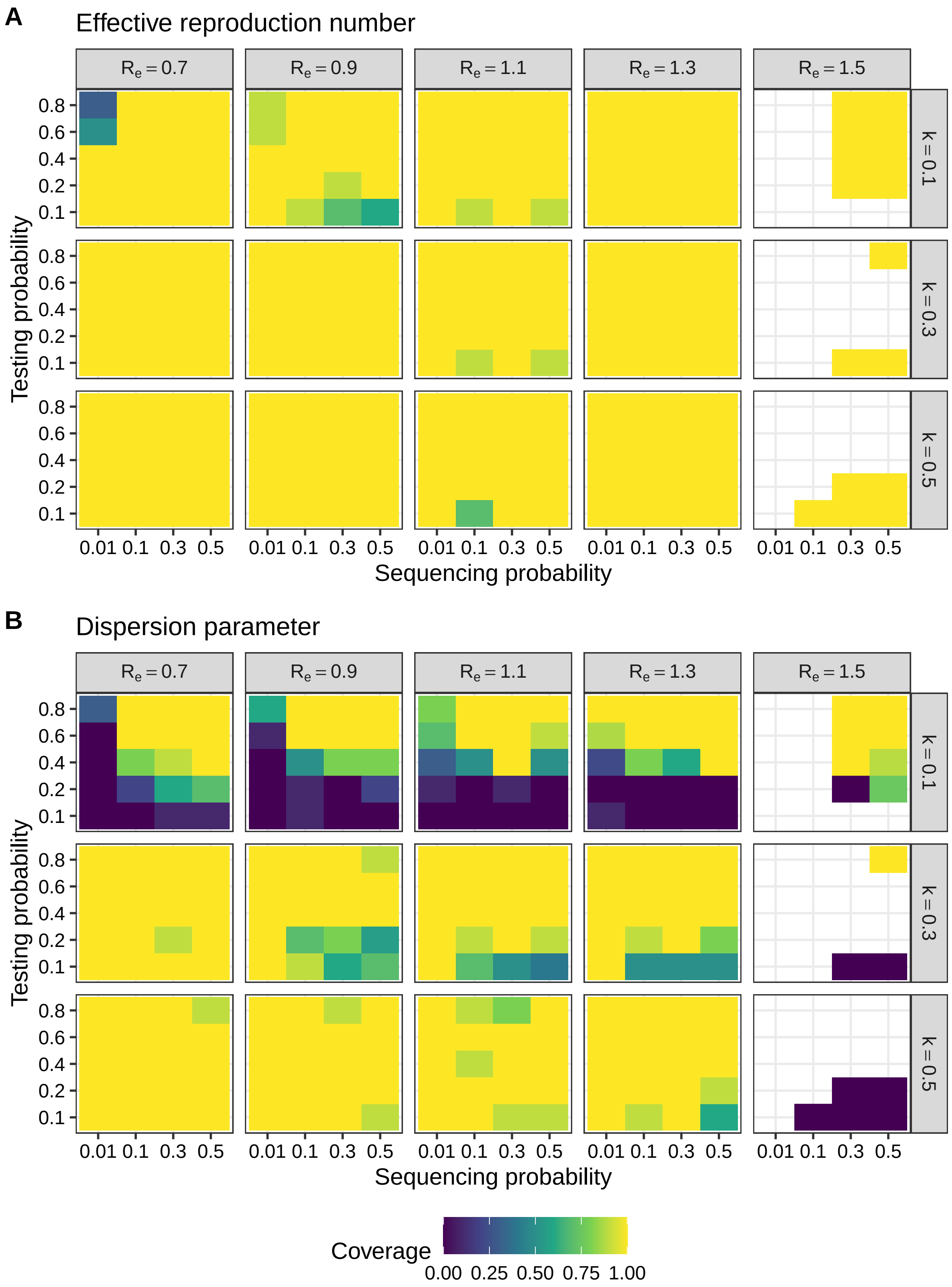
A
Effective reproduction number
Re = 0.7
Re = 0.9
Re = 1.1
Re = 1.3
Re = 1.5
k = 0.1
k = 0.3
k = 0.5
Testing probability
0.8
0.6
0.4
0.2
0.1
Sequencing probability
0.01 0.1 0.3 0.5
B
Dispersion parameter
Re = 0.7
Re = 0.9
Re = 1.1
Re = 1.3
Re = 1.5
k = 0.1
k = 0.3
k = 0.5
Testing probability
Sequencing probability
Coverage
0.00 0.25 0.50 0.75 1.00

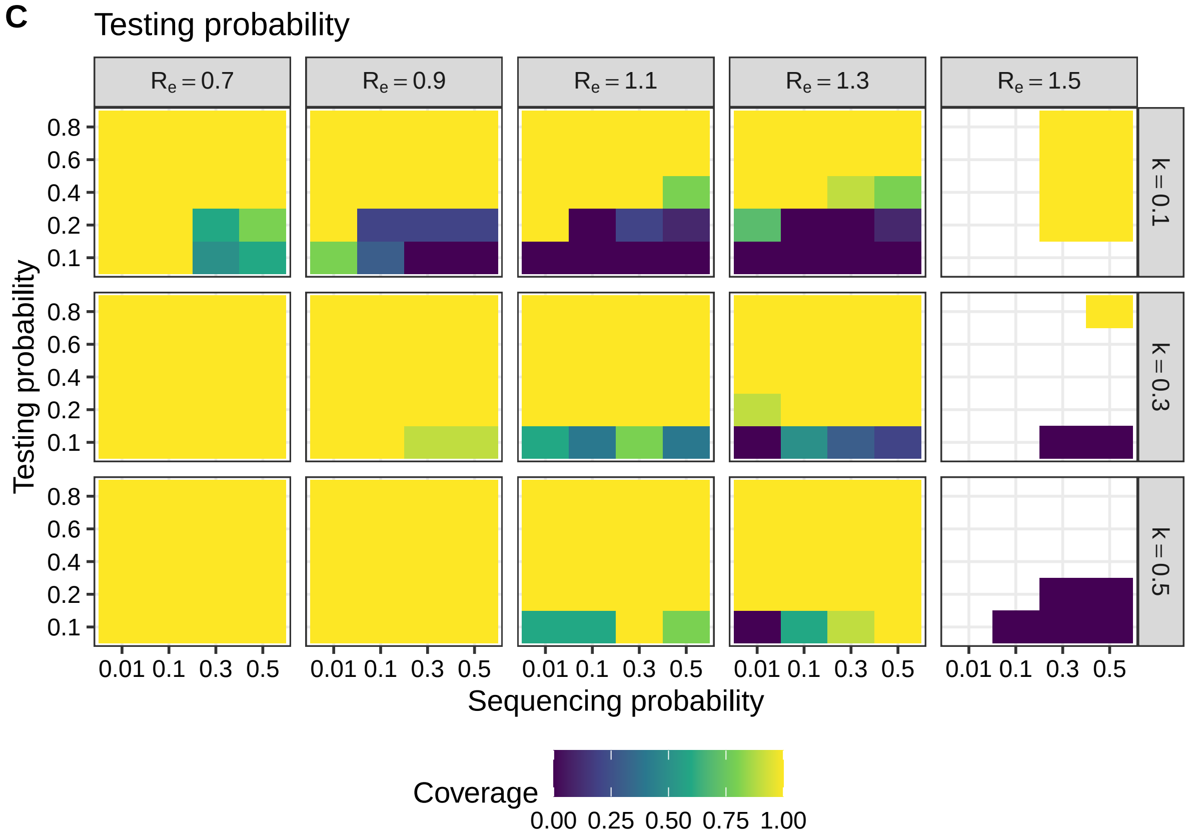

Fig D. Validation of the Bayesian inference model to estimate transmission parameters from the size distribution of identical sequence clusters. A: Coverage of true value by 95% credible interval of the posterior distribution of the effective reproduction number R_e. B: Coverage of true value by 95% credible interval of the posterior distribution of the effective reproduction number k. C: Coverage of true value by 95% credible interval of the posterior distribution of the effective reproduction number M. D: Coverage of true value by 95% credible interval of the posterior distribution of the effective reproduction number τ_{test}. For each parameter combination, we ran the model 10 times on 3000 simulated clusters each.

F Additional results

In the following tables we present the parameter estimates based on the size distribution of identical SARS-CoV-2 sequence clusters in Switzerland, Denmark and Germany in 2021 in numerical form. The results for the effective reproduction number R_e, the dispersion parameter k and the testing probability τ_{test} are also contained in Fig 4 of the manuscript. For each country, month and parameter, estimates are summarized by mean and 95% credible interval of the posterior distribution.

F.1 Switzerland

Month	Effective reproduction number	Dispersion parameter
January	0.902 [0.797-1.035]	0.081 [0.059-0.101]
February	1.114 [0.989-1.275]	0.252 [0.156-0.355]
March	1.095 [0.973-1.254]	0.191 [0.123-0.256]
April	1.053 [0.932-1.212]	0.232 [0.157-0.303]
May	1.049 [0.919-1.217]	0.248 [0.158-0.336]
June	1.100 [0.975-1.254]	0.183 [0.107-0.259]
July	0.978 [0.863-1.115]	0.195 [0.159-0.236]
August	0.901 [0.793-1.036]	0.129 [0.109-0.149]
September	1.005 [0.890-1.144]	0.156 [0.126-0.184]
October	1.023 [0.907-1.174]	0.212 [0.165-0.255]
November	1.017 [0.911-1.163]	0.115 [0.090-0.138]
December	1.003 [0.892-1.137]	0.024 [0.017-0.030]

Table H1. Results of parameter estimations (Switzerland). Samples of posterior distributions of the effective reproduction number and the dispersion parameter summarized by mean and 95% credible interval.

Month	Mutation probability	Testing probability	Detection probability
January	0.288 [0.203-0.377]	0.869 [0.575-0.995]	0.096 [0.063-0.110]
February	0.281 [0.201-0.370]	0.736 [0.297-0.992]	0.097 [0.039-0.130]
March	0.285 [0.203-0.373]	0.788 [0.382-0.992]	0.092 [0.045-0.116]
April	0.279 [0.196-0.370]	0.781 [0.372-0.991]	0.101 [0.048-0.128]
May	0.283 [0.194-0.381]	0.731 [0.285-0.987]	0.124 [0.049-0.168]
June	0.280 [0.199-0.368]	0.779 [0.340-0.992]	0.167 [0.073-0.213]
July	0.283 [0.195-0.368]	0.885 [0.637-0.997]	0.299 [0.215-0.337]
August	0.289 [0.201-0.380]	0.924 [0.724-0.998]	0.178 [0.139-0.192]
September	0.284 [0.198-0.372]	0.899 [0.653-0.997]	0.141 [0.102-0.156]
October	0.283 [0.199-0.376]	0.835 [0.509-0.994]	0.213 [0.130-0.253]
November	0.283 [0.205-0.373]	0.882 [0.610-0.996]	0.086 [0.059-0.097]
December	0.283 [0.202-0.370]	0.884 [0.603-0.997]	0.029 [0.020-0.033]

Table H2. Results of parameter estimations (Switzerland). Samples of posterior distributions of the mutation probability, the testing probability and the detection probability summarized by mean and 95% credible interval.

F.2 Denmark

Month	Effective reproduction number	Dispersion parameter
January	1.228 [1.093-1.391]	0.400 [0.310-0.478]
February	1.282 [1.133-1.471]	0.556 [0.401-0.695]
March	1.214 [1.085-1.373]	0.512 [0.423-0.591]
April	1.248 [1.112-1.419]	0.522 [0.430-0.611]
May	1.184 [1.052-1.351]	0.351 [0.313-0.390]
June	1.294 [1.157-1.461]	0.343 [0.247-0.434]
July	1.058 [0.937-1.207]	0.281 [0.254-0.310]
August	1.017 [0.904-1.170]	0.420 [0.385-0.457]
September	1.125 [1.000-1.284]	0.407 [0.344-0.465]
October	1.180 [1.052-1.345]	0.402 [0.361-0.445]
November	1.131 [1.005-1.285]	0.248 [0.229-0.265]
December	1.218 [1.094-1.379]	0.134 [0.117-0.149]

Table I1. Results of parameter estimations (Denmark). Samples of posterior distributions of the effective reproduction number and the dispersion parameter summarized by mean and 95% credible interval.

Month	Mutation probability	Testing probability	Detection probability
January	0.278 [0.198-0.367]	0.757 [0.316-0.992]	0.349 [0.146-0.457]
February	0.278 [0.189-0.370]	0.630 [0.146-0.984]	0.454 [0.105-0.709]
March	0.278 [0.199-0.365]	0.778 [0.363-0.993]	0.657 [0.306-0.839]
April	0.278 [0.197-0.365]	0.785 [0.345-0.993]	0.562 [0.247-0.711]
May	0.278 [0.195-0.371]	0.892 [0.618-0.997]	0.695 [0.481-0.777]
June	0.274 [0.193-0.359]	0.748 [0.317-0.993]	0.326 [0.138-0.433]
July	0.283 [0.198-0.370]	0.946 [0.806-0.998]	0.786 [0.670-0.830]
August	0.280 [0.198-0.373]	0.927 [0.747-0.998]	0.762 [0.614-0.820]
September	0.282 [0.201-0.373]	0.837 [0.498-0.995]	0.719 [0.428-0.855]
October	0.281 [0.200-0.369]	0.904 [0.684-0.998]	0.709 [0.536-0.783]
November	0.280 [0.191-0.372]	0.953 [0.830-0.998]	0.443 [0.386-0.464]
December	0.279 [0.200-0.366]	0.941 [0.787-0.999]	0.110 [0.092-0.117]

Table I2. Results of parameter estimations (Denmark). Samples of posterior distributions of the mutation probability, the testing probability and the detection probability summarized by mean and 95% credible interval.

F.3 Germany

Month	Effective reproduction number	Dispersion parameter
January	1.095 [0.979-1.243]	0.020 [0.013-0.027]
February	1.096 [0.973-1.257]	0.114 [0.090-0.133]
March	1.125 [1.008-1.270]	0.182 [0.150-0.208]
April	1.124 [1.005-1.295]	0.179 [0.148-0.203]
May	1.110 [0.997-1.242]	0.197 [0.164-0.226]
June	1.084 [0.960-1.234]	0.253 [0.190-0.313]
July	1.023 [0.912-1.169]	0.223 [0.176-0.267]
August	0.965 [0.854-1.111]	0.186 [0.165-0.206]
September	0.986 [0.877-1.141]	0.208 [0.188-0.230]
October	1.063 [0.947-1.214]	0.137 [0.117-0.156]
November	1.036 [0.922-1.186]	0.080 [0.069-0.090]
December	0.962 [0.855-1.098]	0.034 [0.031-0.037]

Table J1. Results of parameter estimations (Germany). Samples of posterior distributions of the effective reproduction number and the dispersion parameter summarized by mean and 95% credible interval.

Month	Mutation probability	Testing probability	Detection probability
January	0.282 [0.202-0.368]	0.859 [0.554-0.996]	0.012 [0.008-0.014]
February	0.283 [0.197-0.378]	0.901 [0.662-0.997]	0.073 [0.054-0.081]
March	0.280 [0.198-0.366]	0.896 [0.641-0.998]	0.082 [0.059-0.092]
April	0.279 [0.198-0.374]	0.898 [0.645-0.998]	0.066 [0.047-0.073]
May	0.281 [0.203-0.359]	0.897 [0.649-0.998]	0.081 [0.059-0.091]
June	0.281 [0.197-0.370]	0.836 [0.482-0.995]	0.141 [0.081-0.168]
July	0.282 [0.203-0.374]	0.870 [0.560-0.997]	0.182 [0.117-0.208]
August	0.286 [0.197-0.379]	0.937 [0.763-0.998]	0.155 [0.126-0.165]
September	0.283 [0.200-0.382]	0.941 [0.796-0.998]	0.135 [0.114-0.143]
October	0.283 [0.198-0.371]	0.919 [0.728-0.997]	0.076 [0.061-0.083]
November	0.282 [0.195-0.375]	0.939 [0.776-0.998]	0.035 [0.029-0.038]
December	0.288 [0.203-0.378]	0.964 [0.878-0.999]	0.038 [0.034-0.039]

Table J2. Results of parameter estimations (Germany). Samples of posterior distributions of the mutation probability, the testing probability and the detection probability summarized by mean and 95% credible interval.

G Sensitivity analysis

We developed several modifications of our inference model to assess our modelling assumptions regarding the mutation and the case detection process. In the following we present the results of the parameter estimations when we use a prior distribution for the yearly mutation rate M instead of a prior distribution for the mutation probability μ to model the mutation process. Furthermore, we also reran our model using constant values for the testing probability instead of a prior distribution.

As an alternative to using a prior distribution that incorporates uncertainty for the testing probability in our model, we used estimates of the ascertainment rate from [11] as constant values. We set the testing probability for the months of January to August to 57.6% and for the months of September to December to 35%.

G.1 Prior distribution for mutation probability and constant testing probability

We use a beta distribution with shape parameters $\alpha = 27$ and $\beta = 68$ as prior distribution for the mutation probability constant values for the testing probability (57.6% for the months of January to August and 35% for the months of September to December). The following figures and tables present the results.

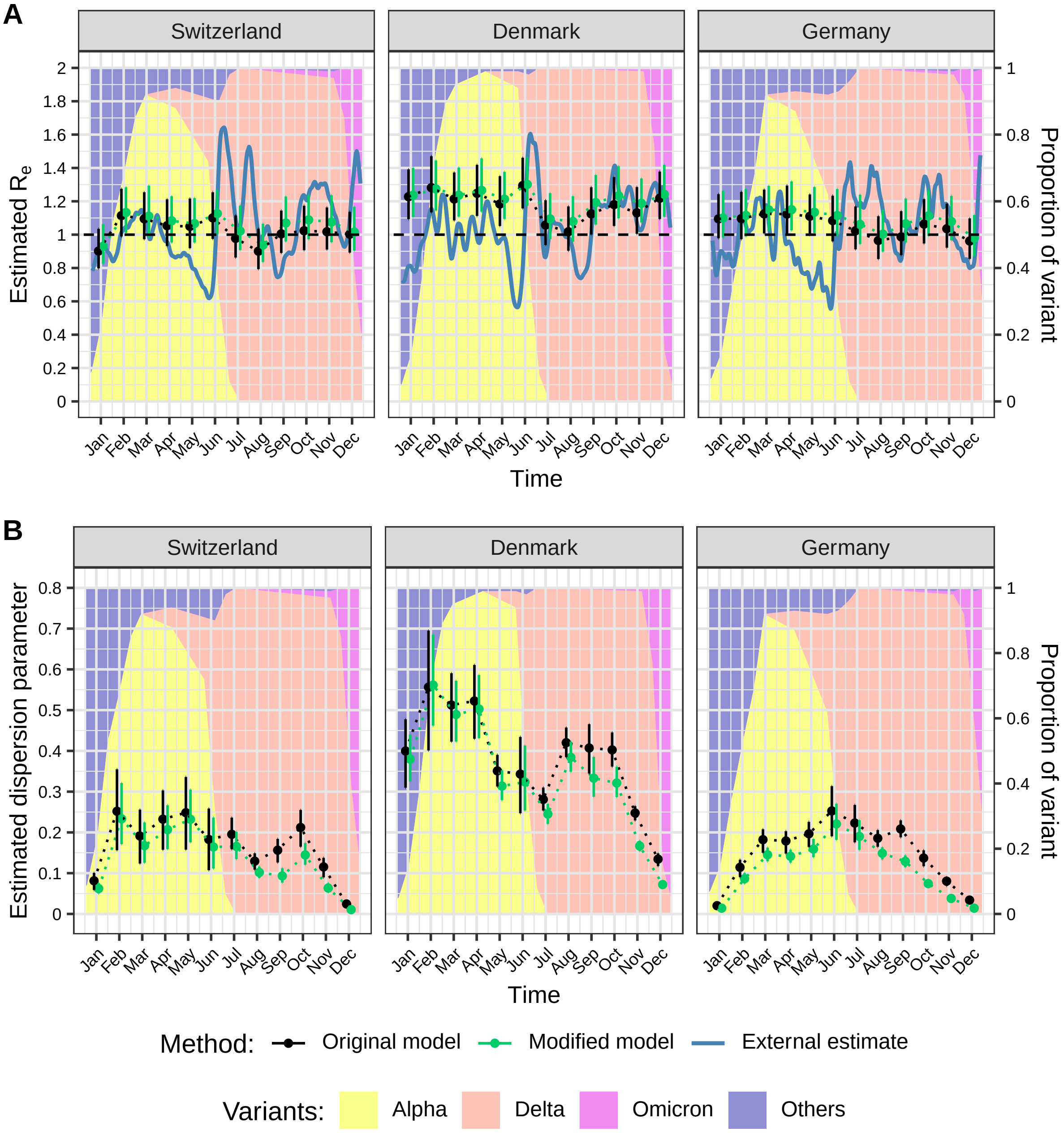

Fig E. Comparison of the estimated effective reproduction numbers and dispersion parameters by country and month. A: Effective reproduction number R_e. B: Dispersion parameter k. The estimates are based on monthly time windows of identical sequence clusters. For each month the estimated means and 95% credible intervals of the posterior distribution obtained by two different models are shown. R_e values are compared to external estimates based on laboratory-confirmed cases (in blue, from github.com/covid-19-Re [12]).

G.1.1 Switzerland

Month	Effective reproduction number	Dispersion parameter
January	0.930 [0.826-1.062]	0.062 [0.050-0.076]
February	1.133 [1.011-1.284]	0.233 [0.171-0.321]
March	1.111 [0.984-1.294]	0.167 [0.126-0.224]
April	1.083 [0.953-1.230]	0.207 [0.159-0.267]
May	1.067 [0.953-1.220]	0.232 [0.176-0.305]
June	1.127 [1.003-1.268]	0.164 [0.111-0.236]
July	1.022 [0.908-1.174]	0.166 [0.135-0.202]
August	0.938 [0.836-1.066]	0.102 [0.089-0.116]
September	1.070 [0.959-1.228]	0.093 [0.077-0.112]
October	1.089 [0.972-1.238]	0.145 [0.121-0.174]
November	1.075 [0.955-1.236]	0.063 [0.053-0.076]
December	1.015 [0.904-1.167]	0.011 [0.009-0.012]

Table K1. Results of parameter estimations with constant testing probability (Switzerland). Samples of posterior distributions of the effective reproduction number and the dispersion parameter summarized by mean and 95% credible interval.

Month	Mutation probability	Testing probability	Detection probability
January	0.289 [0.203-0.379]	0.576	0.063
February	0.282 [0.198-0.368]	0.576	0.076
March	0.280 [0.190-0.383]	0.576	0.067
April	0.282 [0.190-0.370]	0.576	0.074
May	0.281 [0.200-0.371]	0.576	0.098
June	0.284 [0.204-0.366]	0.576	0.124
July	0.283 [0.201-0.378]	0.576	0.195
August	0.284 [0.198-0.373]	0.576	0.111
September	0.283 [0.202-0.378]	0.350	0.055
October	0.279 [0.193-0.367]	0.350	0.089
November	0.286 [0.201-0.379]	0.350	0.034
December	0.282 [0.202-0.372]	0.350	0.012

Table K2. Results of parameter estimations with constant testing probability (Switzerland). Samples of posterior distributions of the mutation probability summarized by mean and 95% credible interval and constant input values of the testing probability and the detection probability.

G.1.2 Denmark

Month	Effective reproduction number	Dispersion parameter
January	1.237 [1.107-1.401]	0.380 [0.325-0.440]
February	1.274 [1.144-1.445]	0.561 [0.462-0.685]
March	1.236 [1.108-1.403]	0.489 [0.423-0.572]
April	1.266 [1.138-1.458]	0.503 [0.431-0.586]
May	1.213 [1.094-1.377]	0.314 [0.279-0.352]
June	1.301 [1.173-1.464]	0.324 [0.254-0.413]
July	1.094 [0.973-1.249]	0.245 [0.221-0.270]
August	1.079 [0.957-1.230]	0.384 [0.349-0.423]
September	1.192 [1.060-1.358]	0.333 [0.287-0.385]
October	1.233 [1.098-1.410]	0.321 [0.287-0.361]
November	1.188 [1.073-1.337]	0.167 [0.154-0.180]
December	1.242 [1.108-1.418]	0.072 [0.065-0.081]

Table L1. Results of parameter estimations with constant testing probability (Denmark). Samples of posterior distributions of the effective reproduction number and the dispersion parameter summarized by mean and 95% credible interval.

Month	Mutation probability	Testing probability	Detection probability
January	0.275 [0.194-0.362]	0.576	0.266
February	0.273 [0.192-0.362]	0.576	0.415
March	0.280 [0.201-0.365]	0.576	0.487
April	0.279 [0.203-0.376]	0.576	0.412
May	0.278 [0.201-0.366]	0.576	0.449
June	0.274 [0.198-0.358]	0.576	0.251
July	0.276 [0.190-0.366]	0.576	0.479
August	0.283 [0.195-0.377]	0.576	0.473
September	0.279 [0.193-0.370]	0.350	0.301
October	0.275 [0.189-0.368]	0.350	0.274
November	0.276 [0.200-0.358]	0.350	0.163
December	0.275 [0.192-0.366]	0.350	0.041

Table L2. Results of parameter estimations with constant testing probability (Denmark). Samples of posterior distributions of the mutation probability summarized by mean and 95% credible interval and constant input values of the testing probability and the detection probability.

G.1.3 Germany

Month	Effective reproduction number	Dispersion parameter
January	1.100 [0.982-1.262]	0.014 [0.011-0.019]
February	1.115 [0.989-1.273]	0.087 [0.074-0.100]
March	1.149 [1.024-1.293]	0.145 [0.129-0.163]
April	1.150 [1.026-1.319]	0.141 [0.126-0.158]
May	1.134 [1.010-1.286]	0.159 [0.139-0.184]
June	1.115 [0.993-1.273]	0.221 [0.182-0.270]
July	1.061 [0.944-1.239]	0.190 [0.157-0.230]
August	1.003 [0.898-1.147]	0.149 [0.134-0.164]
September	1.065 [0.953-1.217]	0.129 [0.115-0.144]
October	1.113 [0.993-1.268]	0.074 [0.066-0.084]
November	1.079 [0.966-1.232]	0.038 [0.034-0.043]
December	0.979 [0.872-1.117]	0.014 [0.013-0.015]

Table M1. Results of parameter estimations with constant testing probability (Germany). Samples of posterior distributions of the effective reproduction number and the dispersion parameter summarized by mean and 95% credible interval.

Month	Mutation probability	Testing probability	Detection probability
January	0.282 [0.198-0.374]	0.576	0.008
February	0.280 [0.193-0.373]	0.576	0.047
March	0.278 [0.194-0.361]	0.576	0.053
April	0.279 [0.194-0.374]	0.576	0.042
May	0.277 [0.191-0.366]	0.576	0.052
June	0.281 [0.197-0.375]	0.576	0.097
July	0.282 [0.197-0.385]	0.576	0.120
August	0.279 [0.198-0.371]	0.576	0.095
September	0.279 [0.195-0.370]	0.350	0.050
October	0.280 [0.198-0.372]	0.350	0.029
November	0.285 [0.204-0.375]	0.350	0.013
December	0.283 [0.198-0.374]	0.350	0.014

Table M2. Results of parameter estimations with constant testing probability (Germany). Samples of posterior distributions of the mutation probability summarized by mean and 95% credible interval and constant input values of the testing probability and the detection probability.

G.2 Constant mutation probability and prior distribution for testing probability

We use a constant mutation probability $\mu = 0.281$ and the same prior distribution for the testing probability as in the original model. The following figures and tables present the results.

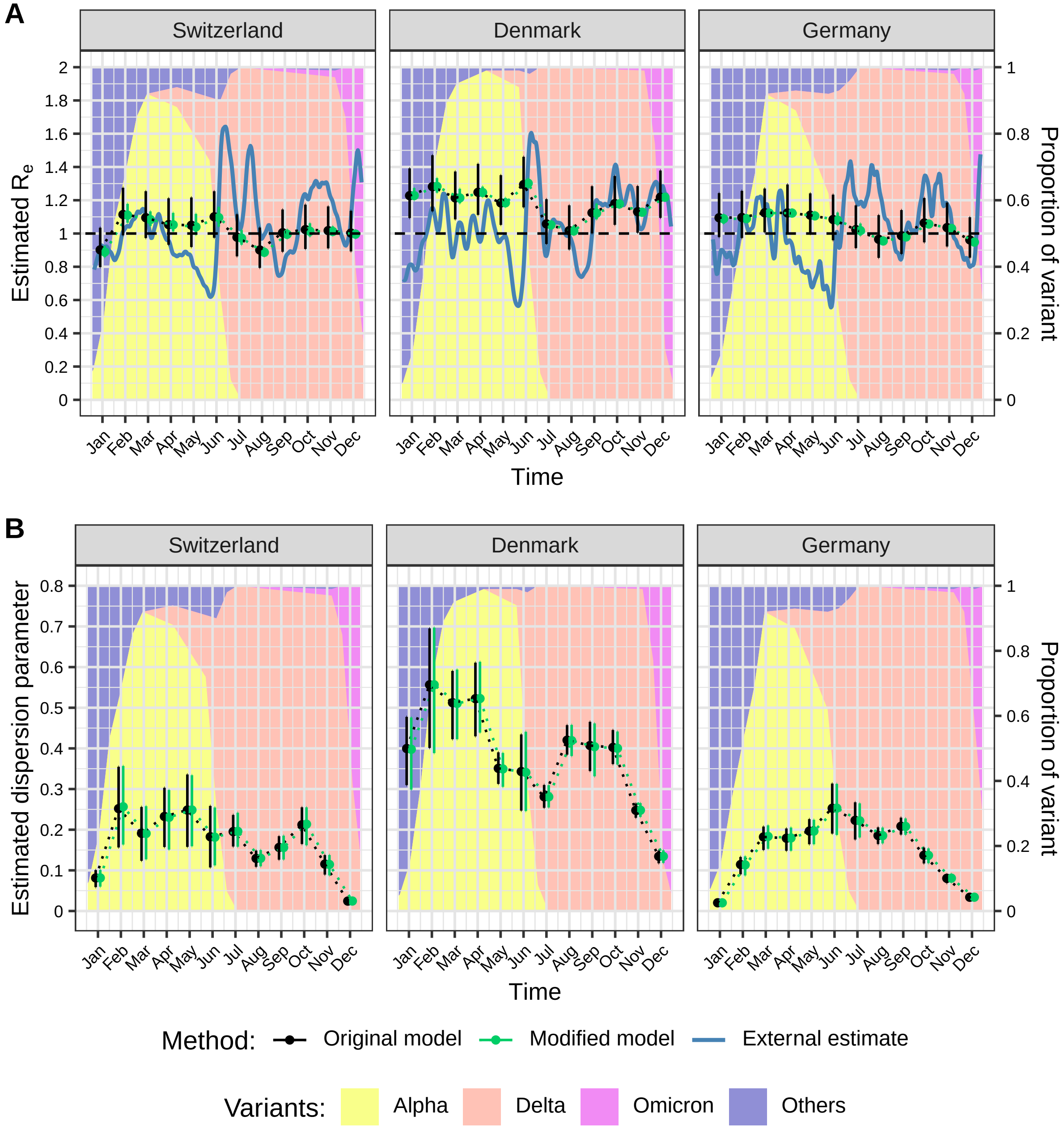

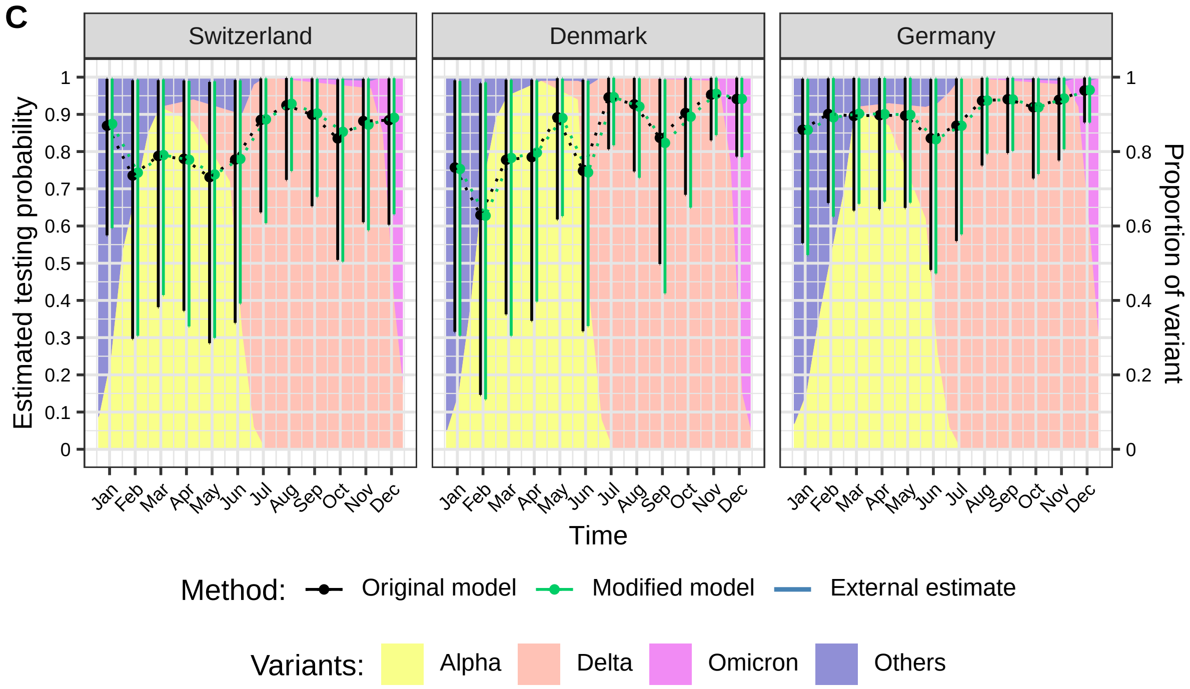

Fig F. Comparison of the estimated effective reproduction numbers, dispersion parameters and testing probabilities by country and month. A: Effective reproduction number R_e. B: Dispersion parameter k. C: Testing probability τ_{test}. The estimates are based on monthly time windows of identical sequence clusters. For each month the estimated means and 95% credible intervals of the posterior distribution obtained by two different models are shown. R_e values are compared to external estimates based on laboratory-confirmed cases (in blue, from github.com/covid-19-Re [12]).

G.2.1 Switzerland

Month	Effective reproduction number	Dispersion parameter
January	0.889 [0.857-0.925]	0.081 [0.060-0.101]
February	1.110 [1.065-1.178]	0.256 [0.163-0.357]
March	1.084 [1.045-1.133]	0.191 [0.127-0.258]
April	1.052 [1.012-1.124]	0.230 [0.151-0.298]
May	1.040 [0.992-1.118]	0.249 [0.159-0.334]
June	1.096 [1.041-1.160]	0.182 [0.112-0.255]
July	0.972 [0.932-1.021]	0.196 [0.158-0.242]
August	0.887 [0.863-0.916]	0.130 [0.110-0.150]
September	0.995 [0.971-1.025]	0.156 [0.127-0.185]
October	1.014 [0.982-1.062]	0.214 [0.162-0.255]
November	1.012 [0.989-1.044]	0.114 [0.088-0.138]
December	0.996 [0.971-1.019]	0.025 [0.018-0.030]

Table N1. Results of parameter estimations with constant mutation probability (Switzerland). Samples of posterior distributions of the effective reproduction number and the dispersion parameter summarized by mean and 95% credible interval.

Month	Mutation probability	Testing probability	Detection probability
January	0.281	0.874 [0.594-0.996]	0.096 [0.066-0.110]
February	0.281	0.743 [0.305-0.993]	0.098 [0.040-0.130]
March	0.281	0.791 [0.414-0.994]	0.092 [0.048-0.116]
April	0.281	0.778 [0.330-0.990]	0.100 [0.043-0.127]
May	0.281	0.738 [0.299-0.989]	0.126 [0.051-0.168]
June	0.281	0.780 [0.392-0.991]	0.168 [0.084-0.213]
July	0.281	0.887 [0.608-0.997]	0.300 [0.206-0.337]
August	0.281	0.928 [0.748-0.998]	0.179 [0.144-0.192]
September	0.281	0.902 [0.679-0.995]	0.141 [0.106-0.156]
October	0.281	0.853 [0.504-0.996]	0.217 [0.129-0.254]
November	0.281	0.872 [0.589-0.996]	0.085 [0.057-0.097]
December	0.281	0.891 [0.632-0.997]	0.029 [0.021-0.033]

Table N2. Results of parameter estimations with constant mutation probability (Switzerland). Constant input values of the mutation probability and samples of posterior distributions of the testing probability and the detection probability summarized by mean and 95% credible interval.

G.2.2 Denmark

Month	Effective reproduction number	Dispersion parameter
January	1.228 [1.200-1.274]	0.398 [0.298-0.476]
February	1.283 [1.245-1.333]	0.557 [0.388-0.697]
March	1.214 [1.181-1.269]	0.511 [0.422-0.594]
April	1.247 [1.221-1.282]	0.523 [0.439-0.613]
May	1.184 [1.160-1.214]	0.349 [0.305-0.389]
June	1.302 [1.275-1.332]	0.341 [0.246-0.441]
July	1.052 [1.025-1.078]	0.281 [0.255-0.310]
August	1.015 [0.992-1.048]	0.419 [0.381-0.458]
September	1.122 [1.085-1.180]	0.404 [0.331-0.461]
October	1.177 [1.155-1.206]	0.400 [0.360-0.441]
November	1.128 [1.115-1.142]	0.248 [0.231-0.265]
December	1.218 [1.208-1.228]	0.134 [0.118-0.149]

Table O1. Results of parameter estimations with constant mutation probability (Denmark). Samples of posterior distributions of the effective reproduction number and the dispersion parameter summarized by mean and 95% credible interval.

Month	Mutation probability	Testing probability	Detection probability
January	0.281	0.753 [0.305-0.989]	0.347 [0.141-0.456]
February	0.281	0.628 [0.134-0.986]	0.452 [0.097-0.711]
March	0.281	0.783 [0.305-0.993]	0.661 [0.258-0.838]
April	0.281	0.797 [0.397-0.991]	0.571 [0.284-0.710]
May	0.281	0.890 [0.627-0.996]	0.693 [0.488-0.776]
June	0.281	0.744 [0.331-0.990]	0.324 [0.144-0.432]
July	0.281	0.946 [0.817-0.998]	0.787 [0.679-0.830]
August	0.281	0.921 [0.730-0.997]	0.757 [0.600-0.819]
September	0.281	0.823 [0.419-0.994]	0.708 [0.360-0.854]
October	0.281	0.894 [0.649-0.998]	0.701 [0.509-0.782]
November	0.281	0.955 [0.844-0.999]	0.444 [0.393-0.464]
December	0.281	0.941 [0.785-0.998]	0.110 [0.092-0.117]

Table O2. Results of parameter estimations with constant mutation probability (Denmark). Constant input values of the mutation probability and samples of posterior distributions of the testing probability and the detection probability summarized by mean and 95% credible interval.

G.2.3 Germany

Month	Effective reproduction number	Dispersion parameter
January	1.088 [1.058-1.116]	0.020 [0.012-0.027]
February	1.089 [1.070-1.113]	0.113 [0.087-0.134]
March	1.122 [1.109-1.144]	0.183 [0.153-0.211]
April	1.123 [1.109-1.144]	0.180 [0.148-0.205]
May	1.106 [1.090-1.130]	0.197 [0.164-0.224]
June	1.080 [1.049-1.126]	0.253 [0.187-0.313]
July	1.017 [0.987-1.060]	0.222 [0.181-0.265]
August	0.955 [0.940-0.977]	0.185 [0.166-0.205]
September	0.979 [0.965-0.997]	0.208 [0.187-0.228]
October	1.057 [1.043-1.078]	0.137 [0.117-0.154]
November	1.030 [1.018-1.044]	0.081 [0.070-0.091]
December	0.948 [0.937-0.959]	0.034 [0.031-0.037]

Table P1. Results of parameter estimations with constant mutation probability (Germany). Samples of posterior distributions of the effective reproduction number and the dispersion parameter summarized by mean and 95% credible interval.

Month	Mutation probability	Testing probability	Detection probability
January	0.281	0.858 [0.522-0.995]	0.012 [0.007-0.014]
February	0.281	0.892 [0.625-0.998]	0.073 [0.051-0.081]
March	0.281	0.902 [0.660-0.997]	0.083 [0.061-0.092]
April	0.281	0.901 [0.666-0.998]	0.066 [0.049-0.073]
May	0.281	0.899 [0.663-0.998]	0.082 [0.060-0.091]
June	0.281	0.833 [0.472-0.996]	0.141 [0.080-0.168]
July	0.281	0.869 [0.578-0.996]	0.182 [0.121-0.208]
August	0.281	0.937 [0.795-0.999]	0.155 [0.131-0.165]
September	0.281	0.940 [0.802-0.998]	0.135 [0.115-0.143]
October	0.281	0.919 [0.740-0.997]	0.076 [0.062-0.083]
November	0.281	0.943 [0.806-0.999]	0.035 [0.030-0.038]
December	0.281	0.965 [0.878-0.999]	0.038 [0.034-0.039]

Table P2. Results of parameter estimations with constant mutation probability (Germany). Constant input values of the mutation probability and samples of posterior distributions of the testing probability and the detection probability summarized by mean and 95% credible interval.

G.3 Constant mutation probability and constant testing probability

We use a constant mutation probability $\mu = 28.1\%$ and constant values for the testing probability (57.6% for the months of January to August and 35% for the months of September to December). The following figures and tables present the results.

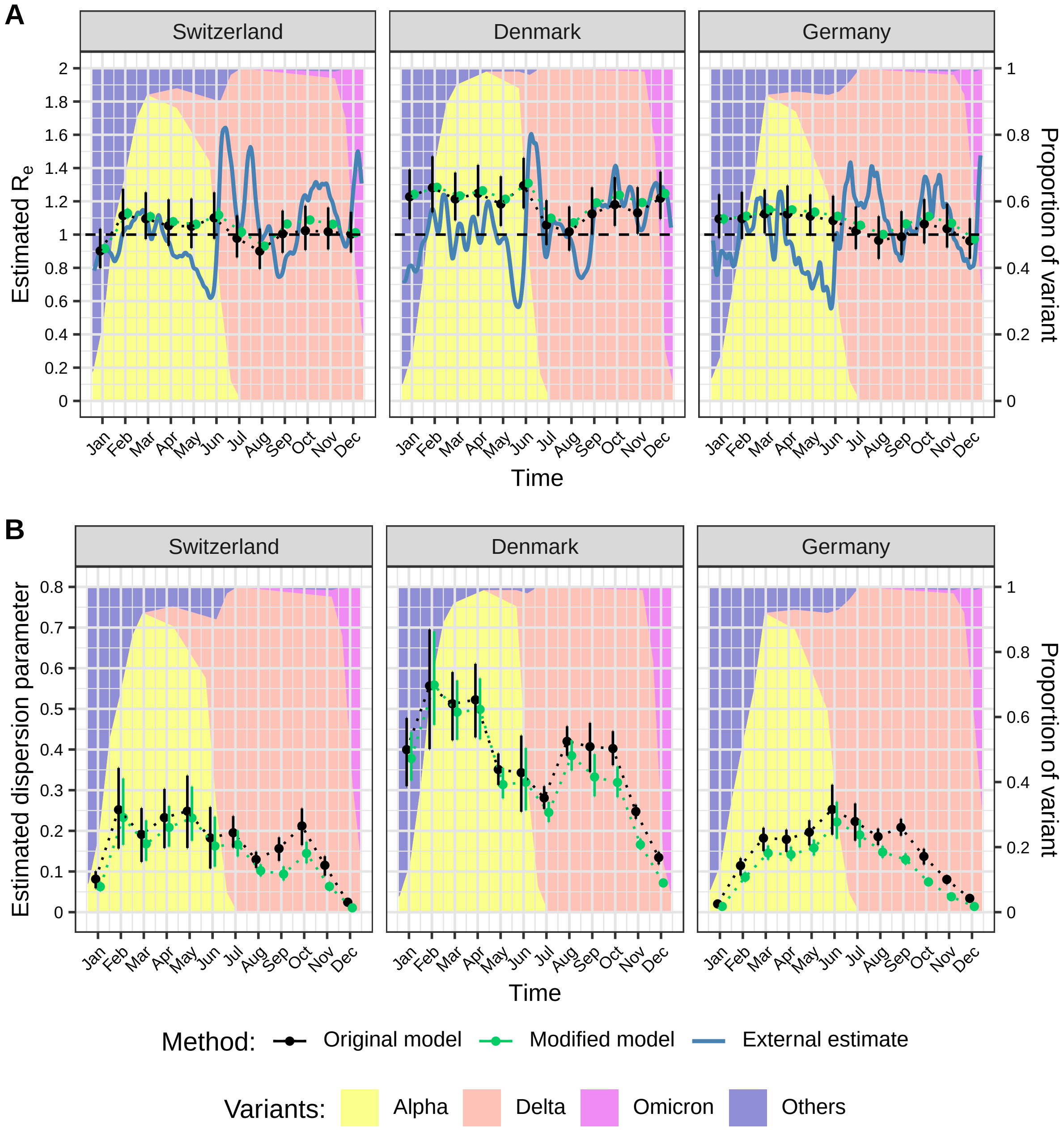

Fig G. Comparison of the estimated effective reproduction numbers and dispersion parameters by country and month. A: Effective reproduction number R_e. B: Dispersion parameter k. The estimates are based on monthly time windows of identical sequence clusters. For each month the estimated means and 95% credible intervals of the posterior distribution obtained by two different models are shown. R_e values are compared to external estimates based on laboratory-confirmed cases (in blue, from github.com/covid-19-Re [12]).

G.3.1 Switzerland

Month	Effective reproduction number	Dispersion parameter
January	0.917 [0.888-0.942]	0.063 [0.052-0.075]
February	1.128 [1.097-1.157]	0.233 [0.166-0.329]
March	1.108 [1.082-1.134]	0.168 [0.126-0.226]
April	1.078 [1.054-1.100]	0.208 [0.161-0.261]
May	1.062 [1.036-1.087]	0.232 [0.176-0.309]
June	1.117 [1.073-1.159]	0.163 [0.112-0.235]
July	1.015 [0.983-1.047]	0.166 [0.137-0.201]
August	0.931 [0.911-0.952]	0.102 [0.088-0.118]
September	1.064 [1.042-1.084]	0.094 [0.078-0.113]
October	1.088 [1.067-1.108]	0.145 [0.120-0.173]
November	1.063 [1.043-1.081]	0.063 [0.053-0.074]
December	1.011 [0.987-1.035]	0.011 [0.009-0.013]

Table Q1. Results of parameter estimations with constant mutation probability and constant testing probability (Switzerland). Samples of posterior distributions of the effective reproduction number and the dispersion parameter summarized by mean and 95% credible interval.

Month	Mutation probability	Testing probability	Detection probability
January	0.281	0.576	0.063
February	0.281	0.576	0.076
March	0.281	0.576	0.067
April	0.281	0.576	0.074
May	0.281	0.576	0.098
June	0.281	0.576	0.124
July	0.281	0.576	0.195
August	0.281	0.576	0.111
September	0.281	0.350	0.055
October	0.281	0.350	0.089
November	0.281	0.350	0.034
December	0.281	0.350	0.012

Table Q2. Results of parameter estimations with constant mutation probability and constant testing probability (Switzerland). Constant input values of the mutation probability, the testing probability and the detection probability.

G.3.2 Denmark

Month	Effective reproduction number	Dispersion parameter
January	1.242 [1.227-1.258]	0.378 [0.324-0.443]
February	1.284 [1.267-1.302]	0.559 [0.460-0.691]
March	1.233 [1.214-1.251]	0.492 [0.424-0.570]
April	1.264 [1.248-1.280]	0.499 [0.425-0.575]
May	1.213 [1.195-1.230]	0.314 [0.280-0.355]
June	1.309 [1.290-1.328]	0.319 [0.251-0.404]
July	1.099 [1.077-1.120]	0.245 [0.222-0.271]
August	1.071 [1.055-1.087]	0.385 [0.348-0.423]
September	1.191 [1.174-1.209]	0.332 [0.285-0.389]
October	1.238 [1.225-1.250]	0.319 [0.283-0.358]
November	1.193 [1.183-1.202]	0.166 [0.154-0.181]
December	1.247 [1.238-1.256]	0.072 [0.064-0.080]

Table R1. Results of parameter estimations with constant mutation probability and constant testing probability (Denmark). Samples of posterior distributions of the effective reproduction number and the dispersion parameter summarized by mean and 95% credible interval.

Month	Mutation probability	Testing probability	Detection probability
January	0.281	0.576	0.266
February	0.281	0.576	0.415
March	0.281	0.576	0.487
April	0.281	0.576	0.412
May	0.281	0.576	0.449
June	0.281	0.576	0.251
July	0.281	0.576	0.479
August	0.281	0.576	0.473
September	0.281	0.350	0.301
October	0.281	0.350	0.274
November	0.281	0.350	0.163
December	0.281	0.350	0.041

Table R2. Results of parameter estimations with constant mutation probability and constant testing probability (Denmark). Constant input values of the mutation probability, the testing probability and the detection probability.

G.3.3 Germany

Month	Effective reproduction number	Dispersion parameter
January	1.095 [1.064-1.122]	0.014 [0.011-0.018]
February	1.111 [1.095-1.125]	0.086 [0.074-0.099]
March	1.150 [1.139-1.160]	0.145 [0.129-0.164]
April	1.149 [1.139-1.159]	0.142 [0.126-0.161]
May	1.135 [1.124-1.147]	0.158 [0.139-0.179]
June	1.111 [1.091-1.129]	0.222 [0.180-0.272]
July	1.056 [1.034-1.076]	0.190 [0.159-0.227]
August	1.001 [0.989-1.014]	0.148 [0.133-0.163]
September	1.064 [1.054-1.075]	0.130 [0.117-0.145]
October	1.109 [1.098-1.122]	0.074 [0.066-0.085]
November	1.069 [1.056-1.081]	0.038 [0.034-0.043]
December	0.972 [0.962-0.984]	0.014 [0.013-0.015]

Table S1. Results of parameter estimations with constant mutation probability and constant testing probability (Germany). Samples of posterior distributions of the effective reproduction number and the dispersion parameter summarized by mean and 95% credible interval.

Month	Mutation probability	Testing probability	Detection probability
January	0.281	0.576	0.008
February	0.281	0.576	0.047
March	0.281	0.576	0.053
April	0.281	0.576	0.042
May	0.281	0.576	0.052
June	0.281	0.576	0.097
July	0.281	0.576	0.120
August	0.281	0.576	0.095
September	0.281	0.350	0.050
October	0.281	0.350	0.029
November	0.281	0.350	0.013
December	0.281	0.350	0.014

Table S2. Results of parameter estimations with constant mutation probability and constant testing probability (Germany). Constant input values of the mutation probability, the testing probability and the detection probability.

G.4 Prior distribution for yearly mutation rate and prior distribution for testing probability

We use a scaled beta distribution with shape parameters $\alpha = 1$ and $\beta = 3$ defined on the interval $[0.05, 1]$ as prior distribution for the yearly mutation rate M and the same prior distribution for the testing probability as in the original model. The following figures and tables present the results.

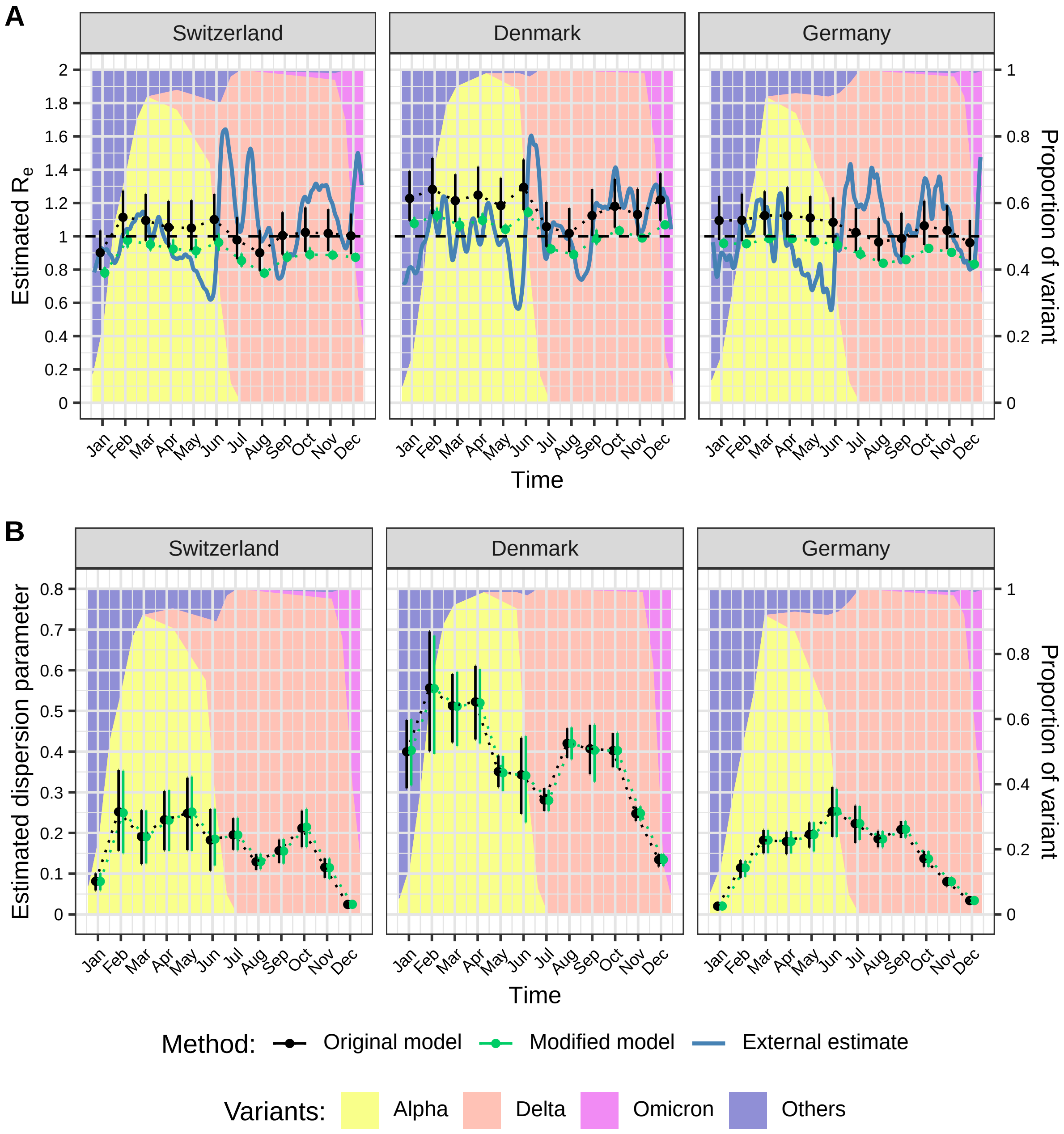

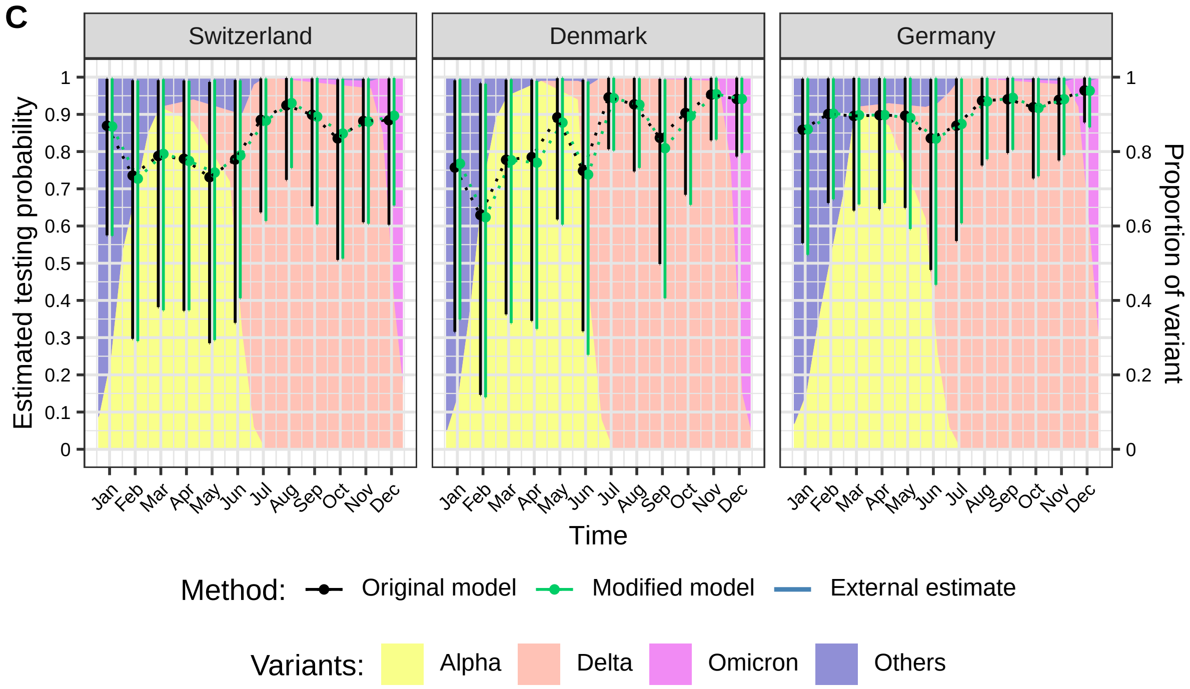

Fig H. Comparison of the estimated effective reproduction numbers, dispersion parameters and testing probabilities by country and month. A: Effective reproduction number R_e. B: Dispersion parameter k. C: Testing probability τ_{test}. The estimates are based on monthly time windows of identical sequence clusters. For each month the estimated means and 95% credible intervals of the posterior distribution obtained by two different models are shown. R_e values are compared to external estimates based on laboratory-confirmed cases (in blue, from github.com/covid-19-Re [12]).

G.4.1 Switzerland

Month	Effective reproduction number	Dispersion parameter	Testing probability
January	0.780 [0.748-0.816]	0.081 [0.059-0.102]	0.867 [0.574-0.997]
February	0.976 [0.932-1.036]	0.250 [0.150-0.353]	0.727 [0.291-0.991]
March	0.951 [0.914-1.000]	0.191 [0.125-0.256]	0.794 [0.374-0.994]
April	0.924 [0.886-0.981]	0.231 [0.156-0.306]	0.775 [0.374-0.990]
May	0.913 [0.869-0.984]	0.252 [0.156-0.339]	0.744 [0.293-0.993]
June	0.963 [0.913-1.017]	0.185 [0.120-0.260]	0.790 [0.406-0.991]
July	0.854 [0.817-0.897]	0.195 [0.157-0.238]	0.883 [0.614-0.996]
August	0.778 [0.755-0.805]	0.130 [0.111-0.149]	0.930 [0.756-0.997]
September	0.874 [0.848-0.908]	0.155 [0.125-0.185]	0.894 [0.604-0.998]
October	0.890 [0.860-0.933]	0.215 [0.165-0.259]	0.848 [0.512-0.996]
November	0.887 [0.863-0.914]	0.115 [0.087-0.137]	0.880 [0.606-0.997]
December	0.874 [0.851-0.896]	0.025 [0.018-0.030]	0.896 [0.655-0.998]

Table T1. Results of parameter estimations (Switzerland). Samples of posterior distributions of the effective reproduction number, the dispersion parameter and the testing probability summarized by mean and 95% credible interval.

Month	Yearly mutation rate	Mutation probability	Detection probability
January	14.002 [13.016-14.982]	0.181 [0.169-0.192]	0.096 [0.063-0.110]
February	14.009 [13.063-14.976]	0.181 [0.170-0.192]	0.095 [0.038-0.130]
March	14.004 [12.956-14.958]	0.181 [0.168-0.192]	0.092 [0.044-0.116]
April	13.996 [13.013-14.940]	0.181 [0.169-0.192]	0.100 [0.048-0.127]
May	13.986 [13.091-14.920]	0.181 [0.170-0.191]	0.127 [0.050-0.169]
June	13.996 [12.967-14.998]	0.181 [0.169-0.192]	0.170 [0.087-0.213]
July	14.011 [13.107-14.967]	0.181 [0.170-0.192]	0.299 [0.208-0.337]
August	14.016 [13.070-14.962]	0.181 [0.170-0.192]	0.179 [0.146-0.192]
September	13.993 [12.998-14.973]	0.181 [0.169-0.192]	0.140 [0.095-0.156]
October	13.983 [13.042-14.973]	0.180 [0.169-0.192]	0.216 [0.131-0.254]
November	13.988 [13.013-14.918]	0.181 [0.169-0.191]	0.086 [0.059-0.097]
December	13.994 [12.966-14.982]	0.181 [0.169-0.192]	0.030 [0.022-0.033]

Table T2. Results of parameter estimations (Switzerland). Samples of posterior distributions of the yearly mutation rate, the mutation probability and the detection probability summarized by mean and 95% credible interval.

G.4.2 Denmark

Month	Effective reproduction number	Dispersion parameter	Testing probability
January	1.077 [1.048-1.116]	0.403 [0.317-0.480]	0.768 [0.349-0.994]
February	1.126 [1.088-1.173]	0.555 [0.395-0.685]	0.624 [0.140-0.982]
March	1.066 [1.033-1.111]	0.511 [0.414-0.596]	0.777 [0.339-0.994]
April	1.096 [1.066-1.138]	0.520 [0.420-0.603]	0.770 [0.324-0.990]
May	1.041 [1.015-1.072]	0.348 [0.303-0.389]	0.878 [0.604-0.998]
June	1.143 [1.116-1.175]	0.341 [0.226-0.438]	0.738 [0.254-0.988]
July	0.924 [0.898-0.951]	0.280 [0.254-0.306]	0.943 [0.803-0.998]
August	0.890 [0.867-0.918]	0.420 [0.381-0.459]	0.926 [0.756-0.997]
September	0.986 [0.950-1.040]	0.403 [0.326-0.466]	0.809 [0.406-0.994]
October	1.033 [1.009-1.062]	0.403 [0.360-0.446]	0.896 [0.657-0.998]
November	0.990 [0.972-1.009]	0.248 [0.230-0.266]	0.954 [0.832-0.999]
December	1.069 [1.052-1.086]	0.135 [0.118-0.149]	0.941 [0.796-0.998]

Table U1. Results of parameter estimations (Denmark). Samples of posterior distributions of the effective reproduction number, the dispersion parameter and the testing probability summarized by mean and 95% credible interval.

Month	Yearly mutation rate	Mutation probability	Detection probability
January	14.030 [13.071-14.993]	0.181 [0.170-0.192]	0.354 [0.161-0.458]
February	14.008 [13.049-14.949]	0.181 [0.170-0.192]	0.449 [0.101-0.708]
March	14.016 [12.964-15.019]	0.181 [0.169-0.193]	0.656 [0.286-0.840]
April	14.024 [12.999-15.031]	0.181 [0.169-0.193]	0.552 [0.232-0.709]
May	14.022 [13.101-14.962]	0.181 [0.170-0.192]	0.684 [0.470-0.777]
June	13.997 [12.987-15.011]	0.181 [0.169-0.192]	0.322 [0.111-0.431]
July	14.030 [13.077-14.957]	0.181 [0.170-0.192]	0.784 [0.667-0.830]
August	14.008 [13.004-15.014]	0.181 [0.169-0.192]	0.761 [0.621-0.820]
September	13.993 [12.920-14.935]	0.181 [0.168-0.192]	0.695 [0.349-0.854]
October	14.003 [12.983-14.976]	0.181 [0.169-0.192]	0.703 [0.515-0.782]
November	13.988 [13.038-14.976]	0.181 [0.169-0.192]	0.444 [0.387-0.464]
December	13.992 [13.050-14.920]	0.181 [0.170-0.191]	0.110 [0.093-0.117]

Table U2. Results of parameter estimations (Denmark). Samples of posterior distributions of the yearly mutation rate, the mutation probability and the detection probability summarized by mean and 95% credible interval.

G.4.3 Germany

Month	Effective reproduction number	Dispersion parameter	Testing probability
January	0.957 [0.929-0.986]	0.020 [0.013-0.028]	0.860 [0.523-0.997]
February	0.955 [0.935-0.975]	0.114 [0.092-0.132]	0.902 [0.672-0.997]
March	0.985 [0.966-1.009]	0.183 [0.150-0.208]	0.898 [0.658-0.998]
April	0.985 [0.967-1.006]	0.179 [0.149-0.205]	0.898 [0.662-0.997]
May	0.971 [0.950-0.997]	0.196 [0.154-0.226]	0.890 [0.592-0.998]
June	0.948 [0.916-0.996]	0.253 [0.190-0.309]	0.835 [0.442-0.997]
July	0.893 [0.864-0.933]	0.223 [0.183-0.266]	0.874 [0.608-0.996]
August	0.838 [0.820-0.859]	0.185 [0.165-0.204]	0.935 [0.779-0.998]
September	0.859 [0.843-0.879]	0.209 [0.188-0.229]	0.944 [0.805-0.999]
October	0.927 [0.909-0.948]	0.136 [0.116-0.155]	0.917 [0.734-0.997]
November	0.903 [0.886-0.921]	0.080 [0.069-0.091]	0.941 [0.791-0.998]
December	0.832 [0.818-0.847]	0.034 [0.030-0.037]	0.963 [0.865-0.998]

Table V1. Results of parameter estimations (Germany). Samples of posterior distributions of the effective reproduction number, the dispersion parameter and the testing probability summarized by mean and 95% credible interval.

Month	Yearly mutation rate	Mutation probability	Detection probability
January	14.041 [13.101-15.026]	0.181 [0.170-0.193]	0.012 [0.007-0.014]
February	13.990 [12.999-14.888]	0.181 [0.169-0.191]	0.073 [0.055-0.081]
March	14.004 [13.050-15.007]	0.181 [0.170-0.192]	0.083 [0.061-0.092]
April	14.032 [12.965-14.993]	0.181 [0.169-0.192]	0.066 [0.048-0.073]
May	13.986 [13.031-14.967]	0.181 [0.169-0.192]	0.081 [0.054-0.091]
June	14.006 [13.043-15.017]	0.181 [0.169-0.192]	0.141 [0.075-0.168]
July	14.012 [12.998-14.996]	0.181 [0.169-0.192]	0.183 [0.127-0.208]
August	13.992 [13.098-15.029]	0.181 [0.170-0.193]	0.154 [0.129-0.165]
September	13.996 [13.093-15.012]	0.181 [0.170-0.192]	0.135 [0.115-0.143]
October	14.017 [13.020-14.973]	0.181 [0.169-0.192]	0.076 [0.061-0.083]
November	13.951 [12.994-15.005]	0.180 [0.169-0.192]	0.035 [0.030-0.038]
December	14.009 [13.003-15.045]	0.181 [0.169-0.193]	0.038 [0.034-0.039]

Table V2. Results of parameter estimations (Germany). Samples of posterior distributions of the yearly mutation rate, the mutation probability and the detection probability summarized by mean and 95% credible interval.

G.5 Prior distribution for yearly mutation rate and constant testing probability

We use a scaled beta distribution with shape parameters $\alpha = 1$ and $\beta = 3$ defined on the interval $[0.05, 1]$ as prior distribution for the yearly mutation rate M. Instead of a prior distribution for the testing probability, constant values are used. The following figures and tables present the results.

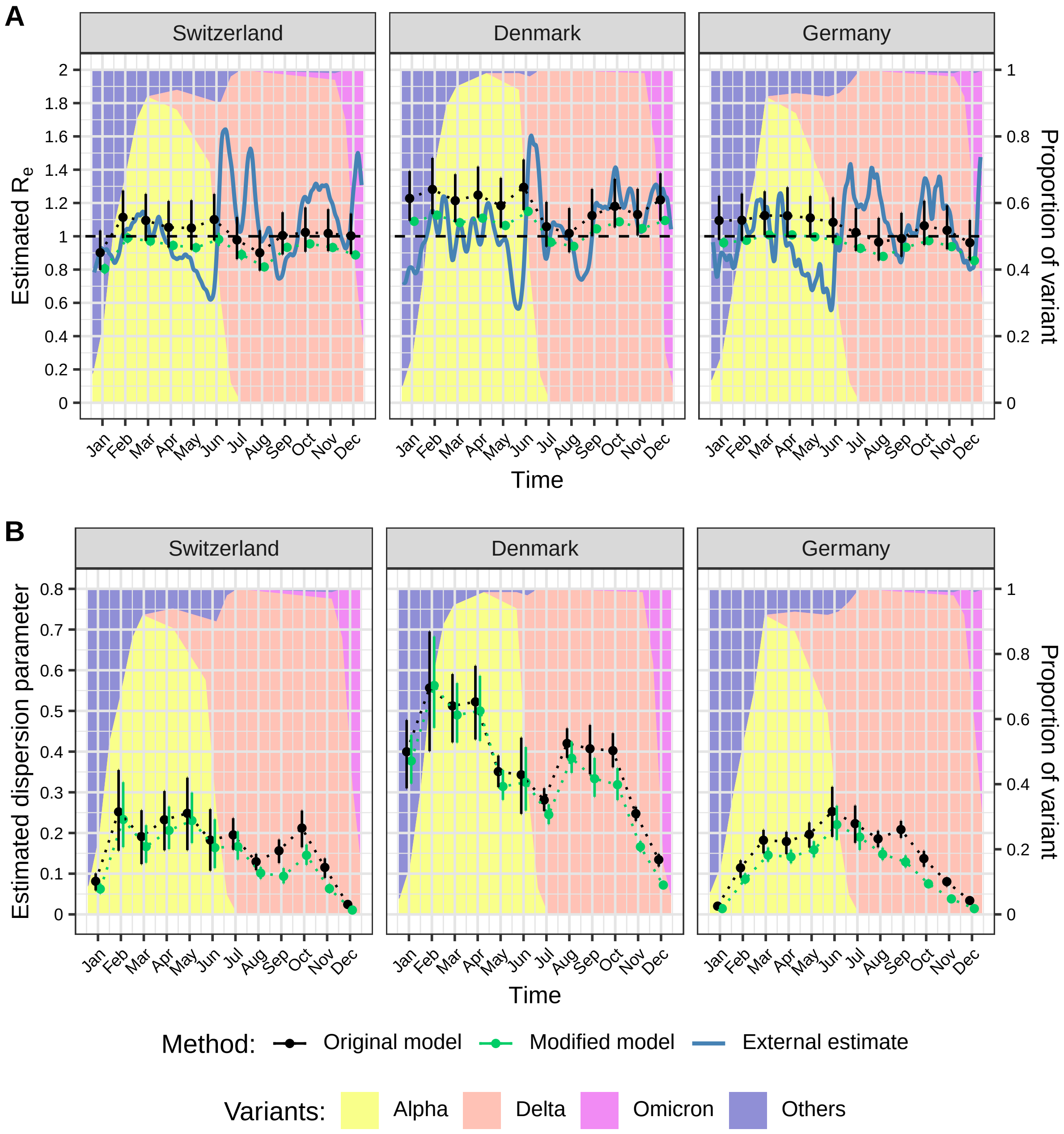

Fig I. Comparison of the estimated effective reproduction numbers and dispersion parameters by country and month. A: Effective reproduction number R_e. B: Dispersion parameter k. The estimates are based on monthly time windows of identical sequence clusters. For each month the estimated means and 95% credible intervals of the posterior distribution obtained by two different models are shown. R_e values are compared to external estimates based on laboratory-confirmed cases (in blue, from github.com/covid-19-Re [12]).

G.5.1 Switzerland

Month	Effective reproduction number	Dispersion parameter	Testing probability
January	0.805 [0.775-0.834]	0.062 [0.051-0.076]	0.576
February	0.990 [0.960-1.019]	0.233 [0.165-0.325]	0.576
March	0.971 [0.944-0.997]	0.166 [0.127-0.219]	0.576
April	0.945 [0.920-0.969]	0.206 [0.161-0.265]	0.576
May	0.932 [0.905-0.957]	0.230 [0.175-0.299]	0.576
June	0.982 [0.942-1.021]	0.164 [0.114-0.234]	0.576
July	0.890 [0.859-0.920]	0.167 [0.135-0.204]	0.576
August	0.816 [0.794-0.838]	0.101 [0.087-0.117]	0.576
September	0.934 [0.911-0.956]	0.094 [0.076-0.114]	0.350
October	0.955 [0.932-0.976]	0.145 [0.122-0.172]	0.350
November	0.933 [0.911-0.955]	0.063 [0.053-0.075]	0.350
December	0.888 [0.866-0.912]	0.011 [0.009-0.013]	0.350

Table W1. Results of parameter estimations with constant testing probability (Switzerland). Samples of posterior distributions of the effective reproduction number and the dispersion parameter summarized by mean and 95% credible interval.

Month	Yearly mutation rate	Mutation probability	Detection probability
January	14.006 [13.036-15.000]	0.181 [0.169-0.192]	0.063
February	13.998 [13.041-15.006]	0.181 [0.169-0.192]	0.076
March	14.008 [13.000-14.928]	0.181 [0.169-0.191]	0.067
April	14.013 [13.062-14.939]	0.181 [0.170-0.192]	0.074
May	13.969 [13.045-14.925]	0.180 [0.169-0.191]	0.098
June	13.988 [13.037-14.919]	0.181 [0.169-0.191]	0.124
July	13.996 [13.053-14.984]	0.181 [0.170-0.192]	0.195
August	13.987 [13.035-15.005]	0.181 [0.169-0.192]	0.111
September	13.998 [13.089-14.965]	0.181 [0.170-0.192]	0.055
October	13.980 [13.001-14.958]	0.180 [0.169-0.192]	0.089
November	14.010 [12.997-15.035]	0.181 [0.169-0.193]	0.034
December	14.008 [13.078-14.973]	0.181 [0.170-0.192]	0.012

Table W2. Results of parameter estimations with constant testing probability (Switzerland). Samples of posterior distributions of the yearly mutation rate and the mutation probability summarized by mean and 95% credible interval.

G.5.2 Denmark

Month	Effective reproduction number	Dispersion parameter	Testing probability
January	1.090 [1.070-1.110]	0.377 [0.321-0.441]	0.576
February	1.127 [1.105-1.149]	0.562 [0.458-0.683]	0.576
March	1.082 [1.058-1.105]	0.490 [0.422-0.568]	0.576
April	1.109 [1.088-1.130]	0.500 [0.426-0.586]	0.576
May	1.065 [1.043-1.086]	0.314 [0.281-0.355]	0.576
June	1.149 [1.126-1.173]	0.324 [0.255-0.411]	0.576
July	0.964 [0.941-0.988]	0.245 [0.222-0.271]	0.576
August	0.940 [0.921-0.958]	0.384 [0.348-0.425]	0.576
September	1.045 [1.024-1.067]	0.333 [0.289-0.385]	0.350
October	1.087 [1.069-1.107]	0.319 [0.281-0.359]	0.350
November	1.047 [1.029-1.064]	0.166 [0.153-0.180]	0.350
December	1.095 [1.077-1.111]	0.072 [0.064-0.081]	0.350

Table X1. Results of parameter estimations with constant testing probability (Denmark). Samples of posterior distributions of the effective reproduction number and the dispersion parameter summarized by mean and 95% credible interval.

Month	Yearly mutation rate	Mutation probability	Detection probability
January	13.979 [13.050-14.925]	0.180 [0.170-0.191]	0.266
February	13.978 [12.993-14.963]	0.180 [0.169-0.192]	0.415
March	13.987 [12.933-14.946]	0.181 [0.168-0.192]	0.487
April	14.005 [13.058-14.970]	0.181 [0.170-0.192]	0.412
May	14.007 [13.042-14.988]	0.181 [0.169-0.192]	0.449
June	14.006 [13.023-15.025]	0.181 [0.169-0.193]	0.251
July	14.004 [12.982-14.969]	0.181 [0.169-0.192]	0.479
August	13.983 [12.996-14.964]	0.180 [0.169-0.192]	0.473
September	13.979 [13.021-14.918]	0.180 [0.169-0.191]	0.301
October	14.043 [13.099-15.041]	0.181 [0.170-0.193]	0.274
November	13.997 [12.996-15.026]	0.181 [0.169-0.193]	0.163
December	14.013 [13.053-14.927]	0.181 [0.170-0.191]	0.041

Table X2. Results of parameter estimations with constant testing probability (Denmark). Samples of posterior distributions of the yearly mutation rate and the mutation probability summarized by mean and 95% credible interval.

G.5.3 Germany

Month	Effective reproduction number	Dispersion parameter	Testing probability
January	0.961 [0.932-0.991]	0.014 [0.011-0.018]	0.576
February	0.976 [0.957-0.994]	0.087 [0.075-0.101]	0.576
March	1.009 [0.991-1.026]	0.145 [0.129-0.164]	0.576
April	1.008 [0.992-1.025]	0.141 [0.125-0.159]	0.576
May	0.997 [0.979-1.013]	0.159 [0.140-0.180]	0.576
June	0.975 [0.953-0.996]	0.221 [0.183-0.267]	0.576
July	0.928 [0.905-0.951]	0.190 [0.158-0.227]	0.576
August	0.879 [0.863-0.895]	0.148 [0.133-0.164]	0.576
September	0.934 [0.918-0.951]	0.129 [0.115-0.145]	0.350
October	0.974 [0.956-0.992]	0.075 [0.066-0.085]	0.350
November	0.938 [0.920-0.956]	0.038 [0.034-0.043]	0.350
December	0.854 [0.838-0.869]	0.014 [0.013-0.015]	0.350

Table Y1. Results of parameter estimations with constant testing probability (Germany). Samples of posterior distributions of the effective reproduction number and the dispersion parameter summarized by mean and 95% credible interval.

Month	Yearly mutation rate	Mutation probability	Detection probability
January	14.012 [13.074-15.007]	0.181 [0.170-0.192]	0.008
February	14.002 [12.981-14.943]	0.181 [0.169-0.192]	0.047
March	13.989 [13.030-14.994]	0.181 [0.169-0.192]	0.053
April	14.011 [13.022-15.013]	0.181 [0.169-0.192]	0.042
May	14.003 [12.990-14.956]	0.181 [0.169-0.192]	0.052
June	13.999 [13.057-14.982]	0.181 [0.170-0.192]	0.097
July	14.032 [13.083-15.020]	0.181 [0.170-0.193]	0.120
August	14.032 [13.061-14.963]	0.181 [0.170-0.192]	0.095
September	14.010 [13.021-15.013]	0.181 [0.169-0.192]	0.050
October	13.965 [12.993-14.988]	0.180 [0.169-0.192]	0.029
November	14.001 [13.048-15.001]	0.181 [0.170-0.192]	0.013
December	14.005 [13.033-14.973]	0.181 [0.169-0.192]	0.014

Table Y2. Results of parameter estimations with constant testing probability (Germany). Samples of posterior distributions of the yearly mutation rate and the mutation probability summarized by mean and 95% credible interval.

H Comparison with literature

In order to compare our model with the work of Tran-Kiem and Bedford [13], we apply our model to the same data set from New Zealand as they did and also use the same parameters. In order to achieve the highest possible comparability, we use constant values for both the mutation probability and the testing probability. We retrieved the cluster data as well as the values for mutation probability, testing probability and sequencing probability from the GitHub repository size-genetic-clusters [14]. We have obtained very similar results and present them in the following figure in the same way as did Tran-Kiem and Bedford in Figure S9 of the supplementary information of [13].

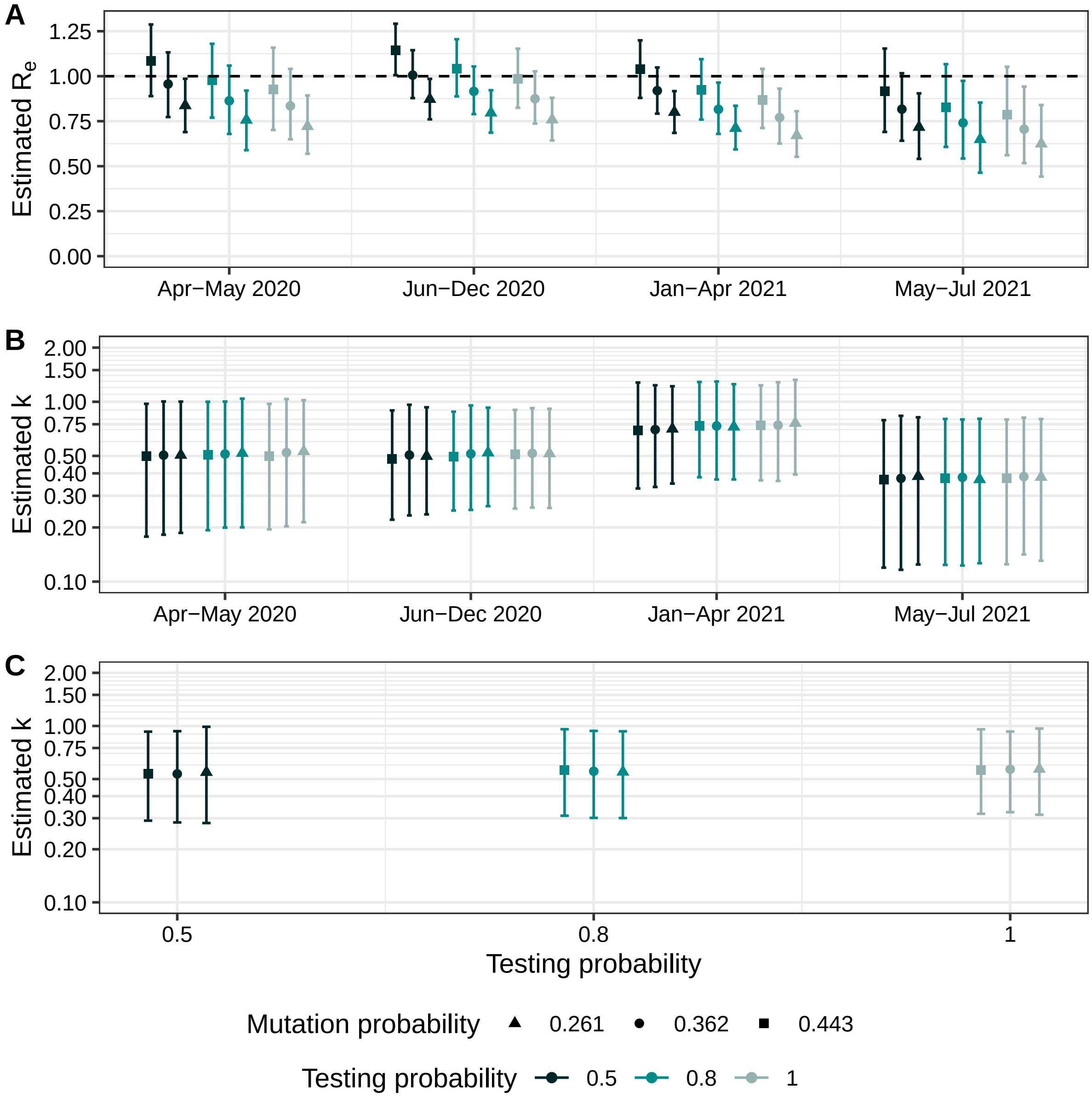

Fig J. Comparison of the estimated effective reproduction numbers and dispersion parameters in New Zealand for different timne periods. A: Effective reproduction number R_e by period. B: Dispersion parameter k by period. C: Dispersion parameter k over whole time period. For each period and combination of mutation probability and testing probability the estimated means and 95% credible intervals of the posterior distribution are shown.

Index	Period	Effective reproduction number	Dispersion parameter
1	Apr-May 2020	0.837 [0.689-0.986]	0.506 [0.187-1.003]
2	Jun-Dec 2020	0.872 [0.760-0.985]	0.498 [0.237-0.932]
3	Jan-Apr 2021	0.800 [0.685-0.917]	0.707 [0.351-1.220]
4	May-Jul 2021	0.717 [0.541-0.905]	0.385 [0.124-0.820]
5	Apr-May 2020	0.956 [0.773-1.133]	0.505 [0.182-1.004]
6	Jun-Dec 2020	1.006 [0.878-1.144]	0.505 [0.233-0.963]
7	Jan-Apr 2021	0.920 [0.792-1.048]	0.700 [0.336-1.235]
8	May-Jul 2021	0.817 [0.641-1.016]	0.375 [0.116-0.835]
9	Apr-May 2020	1.086 [0.889-1.287]	0.499 [0.178-0.974]
10	Jun-Dec 2020	1.144 [1.006-1.292]	0.481 [0.221-0.895]
11	Jan-Apr 2021	1.039 [0.879-1.199]	0.692 [0.329-1.280]
12	May-Jul 2021	0.916 [0.690-1.154]	0.368 [0.120-0.790]
13	Apr-May 2020	0.757 [0.589-0.920]	0.518 [0.200-1.040]
14	Jun-Dec 2020	0.796 [0.686-0.922]	0.521 [0.263-0.927]
15	Jan-Apr 2021	0.711 [0.593-0.836]	0.726 [0.370-1.253]
16	May-Jul 2021	0.649 [0.464-0.854]	0.370 [0.127-0.804]
17	Apr-May 2020	0.863 [0.679-1.059]	0.512 [0.200-1.003]
18	Jun-Dec 2020	0.916 [0.789-1.054]	0.514 [0.250-0.954]
19	Jan-Apr 2021	0.816 [0.679-0.965]	0.733 [0.370-1.296]
20	May-Jul 2021	0.741 [0.543-0.974]	0.380 [0.123-0.797]
21	Apr-May 2020	0.978 [0.770-1.180]	0.506 [0.193-1.000]
22	Jun-Dec 2020	1.041 [0.888-1.205]	0.495 [0.248-0.881]
23	Jan-Apr 2021	0.923 [0.759-1.095]	0.733 [0.380-1.288]
24	May-Jul 2021	0.827 [0.607-1.067]	0.375 [0.124-0.802]

Table Z1. Results of parameter estimations with constant mutation probability and constant testing probability (New Zealand). Samples of posterior distributions of the effective reproduction number and the dispersion parameter summarized by mean and 95% credible interval.

Index	Period	Effective reproduction number	Dispersion parameter
25	Apr-May 2020	0.722 [0.569-0.893]	0.530 [0.214-1.021]
26	Jun-Dec 2020	0.759 [0.643-0.880]	0.516 [0.257-0.914]
27	Jan-Apr 2021	0.671 [0.552-0.805]	0.761 [0.395-1.324]
28	May-Jul 2021	0.625 [0.442-0.840]	0.381 [0.131-0.802]
29	Apr-May 2020	0.835 [0.649-1.040]	0.522 [0.203-1.035]
30	Jun-Dec 2020	0.875 [0.737-1.027]	0.516 [0.258-0.922]
31	Jan-Apr 2021	0.770 [0.626-0.931]	0.741 [0.363-1.285]
32	May-Jul 2021	0.705 [0.517-0.942]	0.383 [0.142-0.816]
33	Apr-May 2020	0.926 [0.701-1.158]	0.498 [0.195-0.973]
34	Jun-Dec 2020	0.984 [0.825-1.153]	0.509 [0.255-0.901]
35	Jan-Apr 2021	0.868 [0.712-1.041]	0.738 [0.365-1.235]
36	May-Jul 2021	0.787 [0.561-1.052]	0.375 [0.125-0.797]

Table Z1. Results of parameter estimations with constant mutation probability and constant testing probability (New Zealand). Samples of posterior distributions of the effective reproduction number and the dispersion parameter summarized by mean and 95% credible interval.

Index	Period	Mutation probability	Testing probability	Detection probability
1	Apr-May 2020	0.261	0.500	0.126
2	Jun-Dec 2020	0.261	0.500	0.254
3	Jan-Apr 2021	0.261	0.500	0.229
4	May-Jul 2021	0.261	0.500	0.240
5	Apr-May 2020	0.362	0.500	0.126
6	Jun-Dec 2020	0.362	0.500	0.254
7	Jan-Apr 2021	0.362	0.500	0.229
8	May-Jul 2021	0.362	0.500	0.240
9	Apr-May 2020	0.443	0.500	0.126
10	Jun-Dec 2020	0.443	0.500	0.254
11	Jan-Apr 2021	0.443	0.500	0.229
12	May-Jul 2021	0.443	0.500	0.240

Table Z2. Results of parameter estimations with constant mutation probability and constant testing probability (New Zealand). Constant input values of the mutation probability, the testing probability and the detection probability.

Index	Period	Mutation probability	Testing probability	Detection probability
13	Apr-May 2020	0.261	0.800	0.202
14	Jun-Dec 2020	0.261	0.800	0.407
15	Jan-Apr 2021	0.261	0.800	0.367
16	May-Jul 2021	0.261	0.800	0.384
17	Apr-May 2020	0.362	0.800	0.202
18	Jun-Dec 2020	0.362	0.800	0.407
19	Jan-Apr 2021	0.362	0.800	0.367
20	May-Jul 2021	0.362	0.800	0.384
21	Apr-May 2020	0.443	0.800	0.202
22	Jun-Dec 2020	0.443	0.800	0.407
23	Jan-Apr 2021	0.443	0.800	0.367
24	May-Jul 2021	0.443	0.800	0.384
25	Apr-May 2020	0.261	1.000	0.252
26	Jun-Dec 2020	0.261	1.000	0.508
27	Jan-Apr 2021	0.261	1.000	0.459
28	May-Jul 2021	0.261	1.000	0.480
29	Apr-May 2020	0.362	1.000	0.252
30	Jun-Dec 2020	0.362	1.000	0.508
31	Jan-Apr 2021	0.362	1.000	0.459
32	May-Jul 2021	0.362	1.000	0.480
33	Apr-May 2020	0.443	1.000	0.252
34	Jun-Dec 2020	0.443	1.000	0.508
35	Jan-Apr 2021	0.443	1.000	0.459
36	May-Jul 2021	0.443	1.000	0.480

Table Z2. Results of parameter estimations with constant mutation probability and constant testing probability (New Zealand). Constant input values of the mutation probability, the testing probability and the detection probability.

I Posterior predictive check

We ran a posterior predictive check to assess whether our simulation of identical sequence clusters is compatible with the cluster data from Switzerland, Denmark and Germany. For each country and month we randomly chose 50 samples each from the posterior distributions of the effective reproduction number R_e, the dispersion parameter k, the mutation probability μ and the detection probability τ obtained with our main model (presented in the paper). We then simulated the same number of clusters observed in the respective country and month, running the simulation once for each of the 50 parameter combinations. We summarized the results of the simulations by the 95% prediction interval, calculated individually for each cluster size. The following figures present the outcome of the posterior predictive check.

I.1 Switzerland

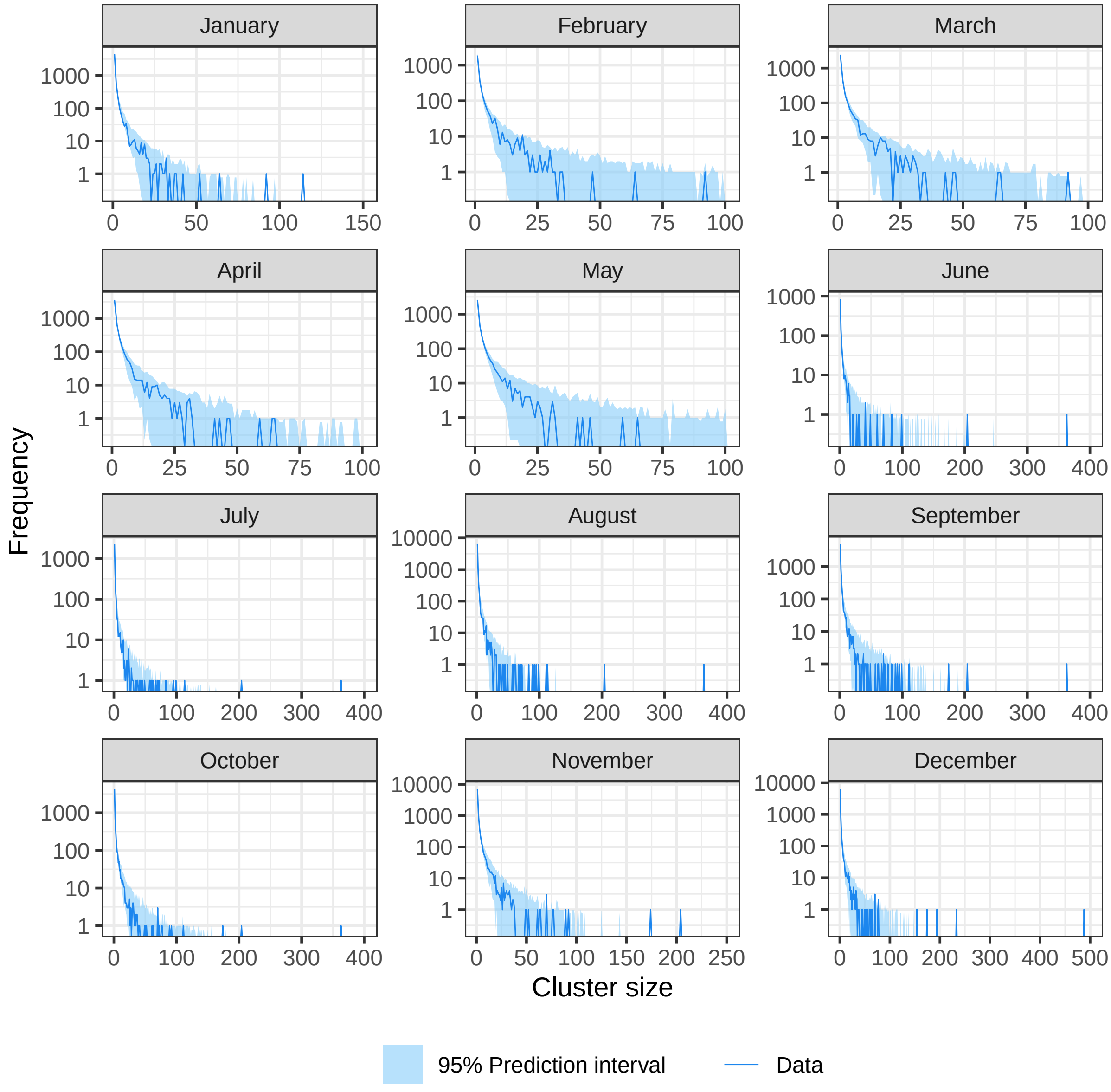

Fig K. Posterior predictive check of simulation of cluster size distribution (Switzerland).

I.2 Denmark

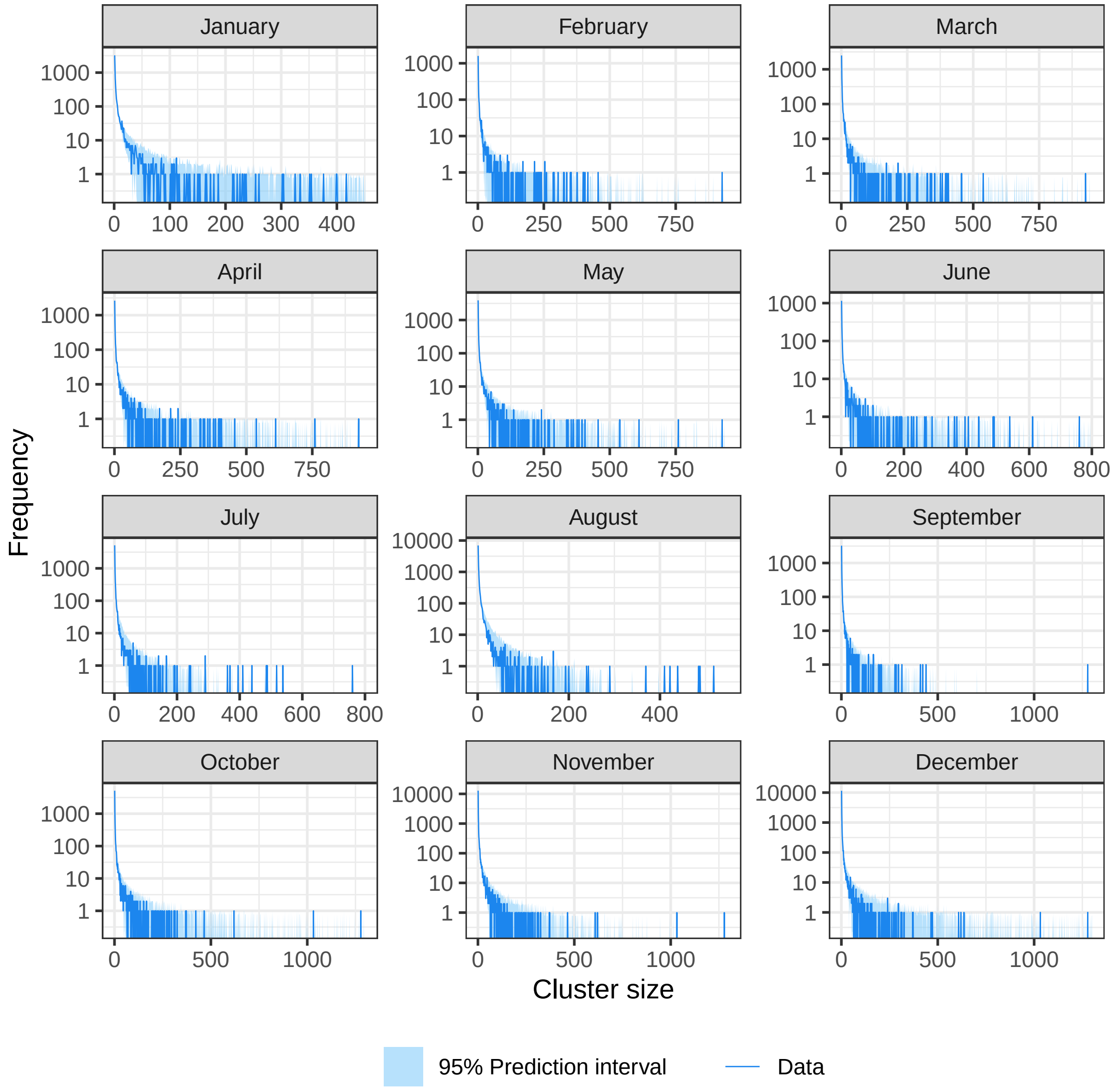

Fig L. Posterior predictive check of simulation of cluster size distribution (Denmark).

I.3 Germany

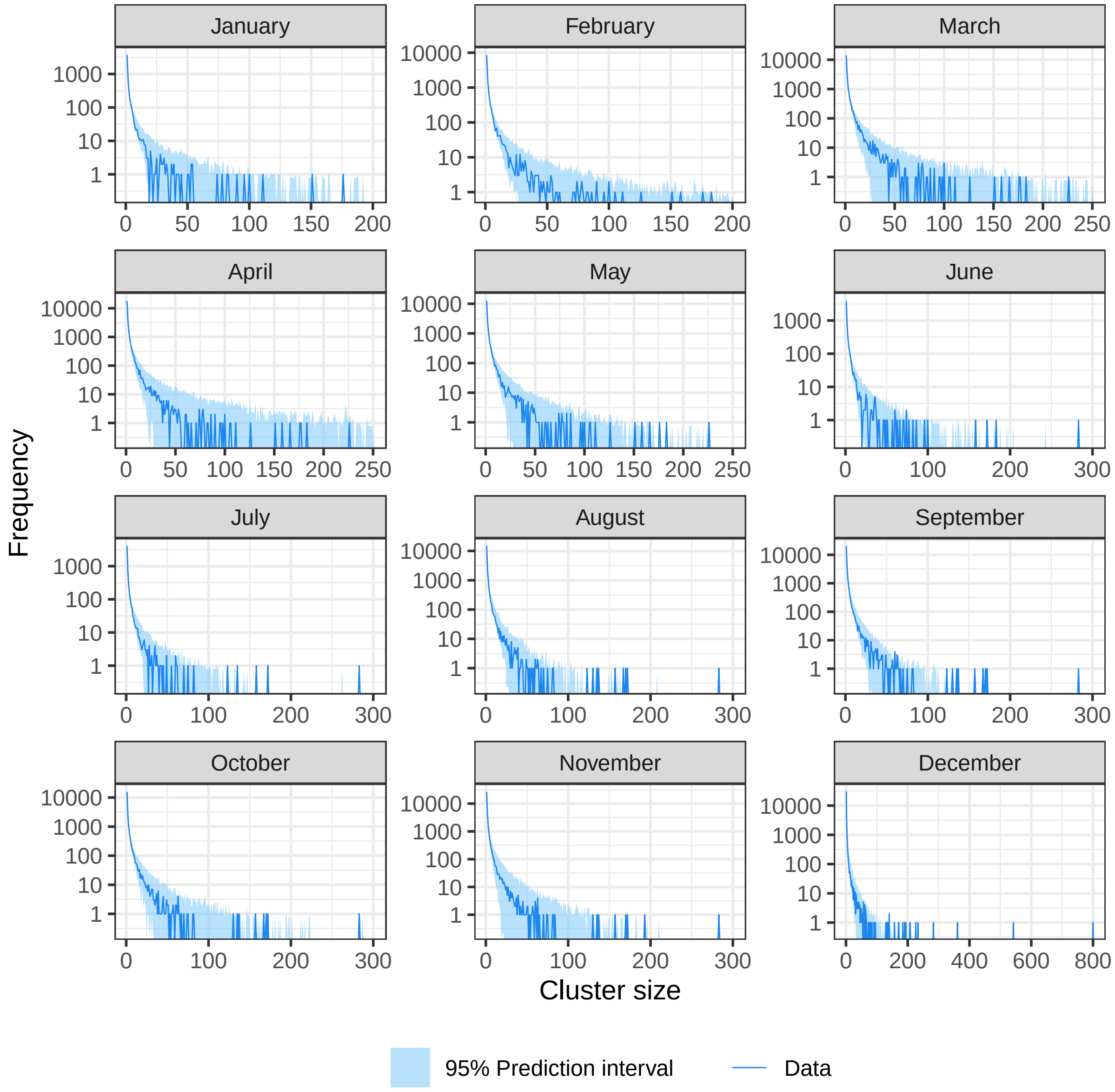

Fig M. Posterior predictive check of simulation of cluster size distribution (Germany).

J Goodness of fit

We performed a goodness of fit check to assess whether our main model of the cluster size distribution (presented in the paper) is compatible with the cluster data from Switzerland, Denmark and Germany. For each country and month we took the estimated mean of the effective reproduction number R_e, the dispersion parameter k, the mutation probability μ and the testing probability τ_{test} as an input for our model of the cluster size distribution. This line is surrounded by an area whose boundaries are the results of the model of the cluster size distribution when the estimated 2.5% and 97.5% quantiles of the estimated posterior distributions of the parameters are used as input. The following figures present the outcome of the goodness of fit check.

J.1 Switzerland

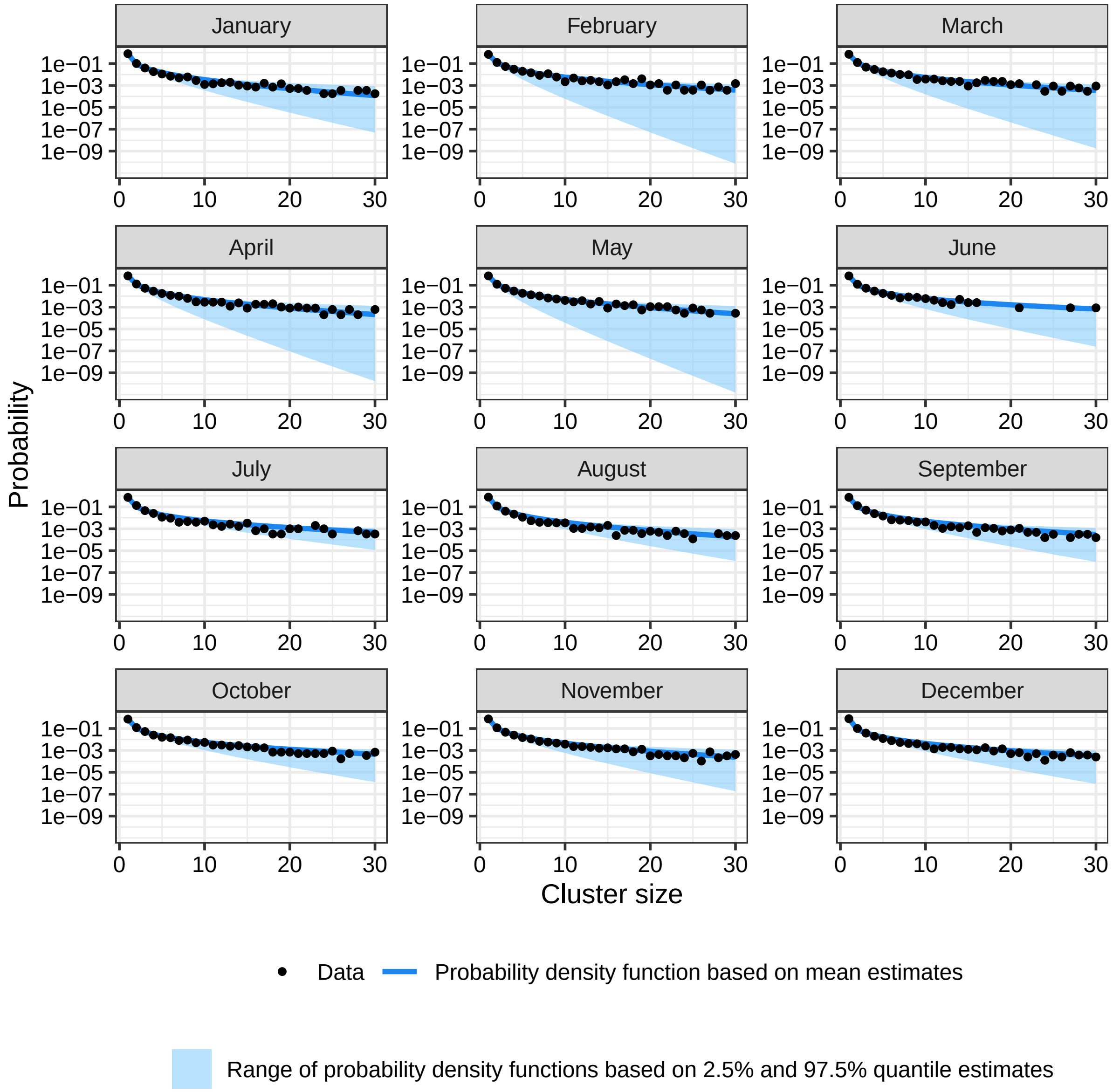

Fig N. Goodness of fit of cluster size distribution (Switzerland).

J.2 Denmark

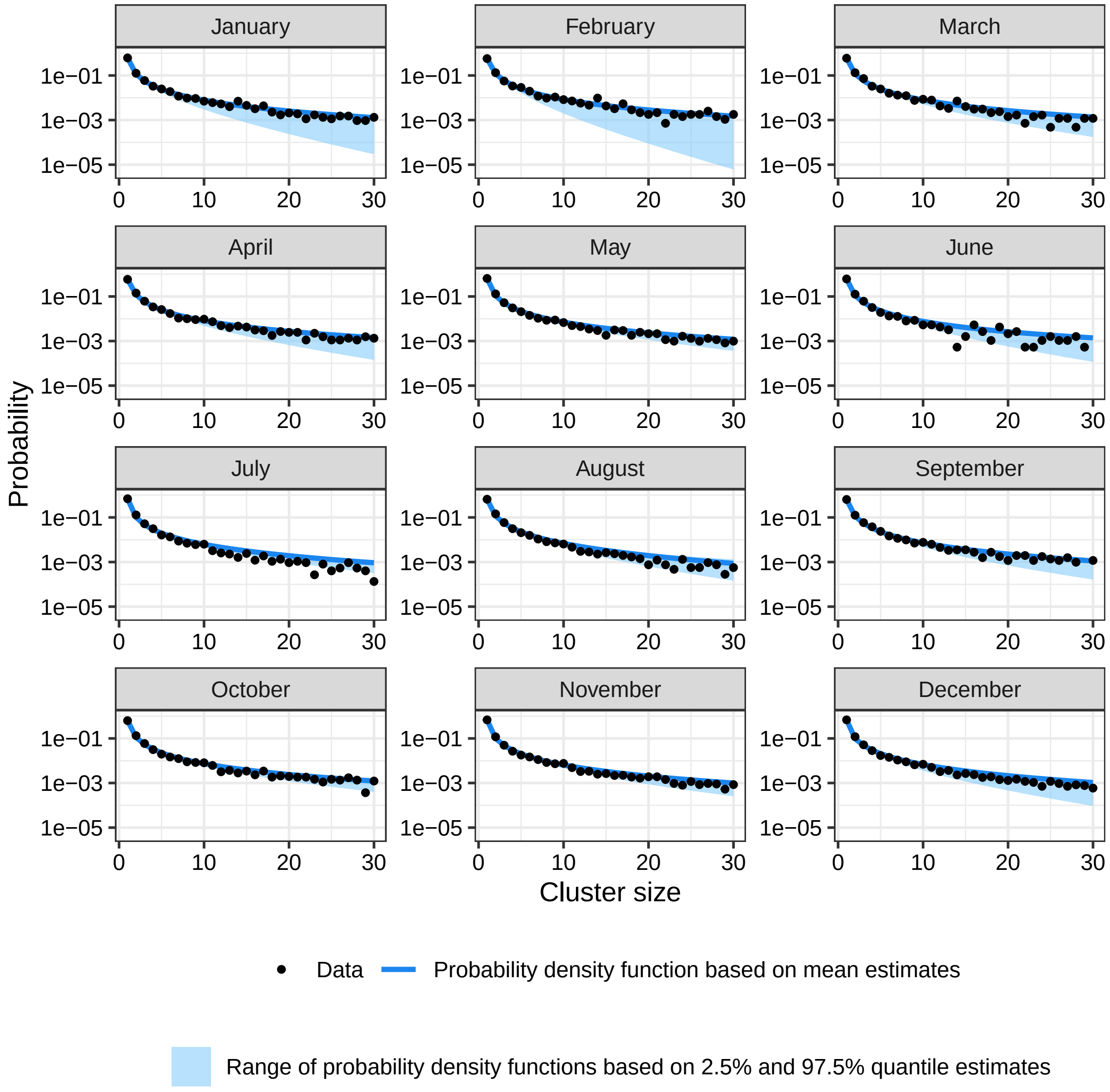

Fig O. Goodness of fit of cluster size distribution (Denmark).

J.3 Germany

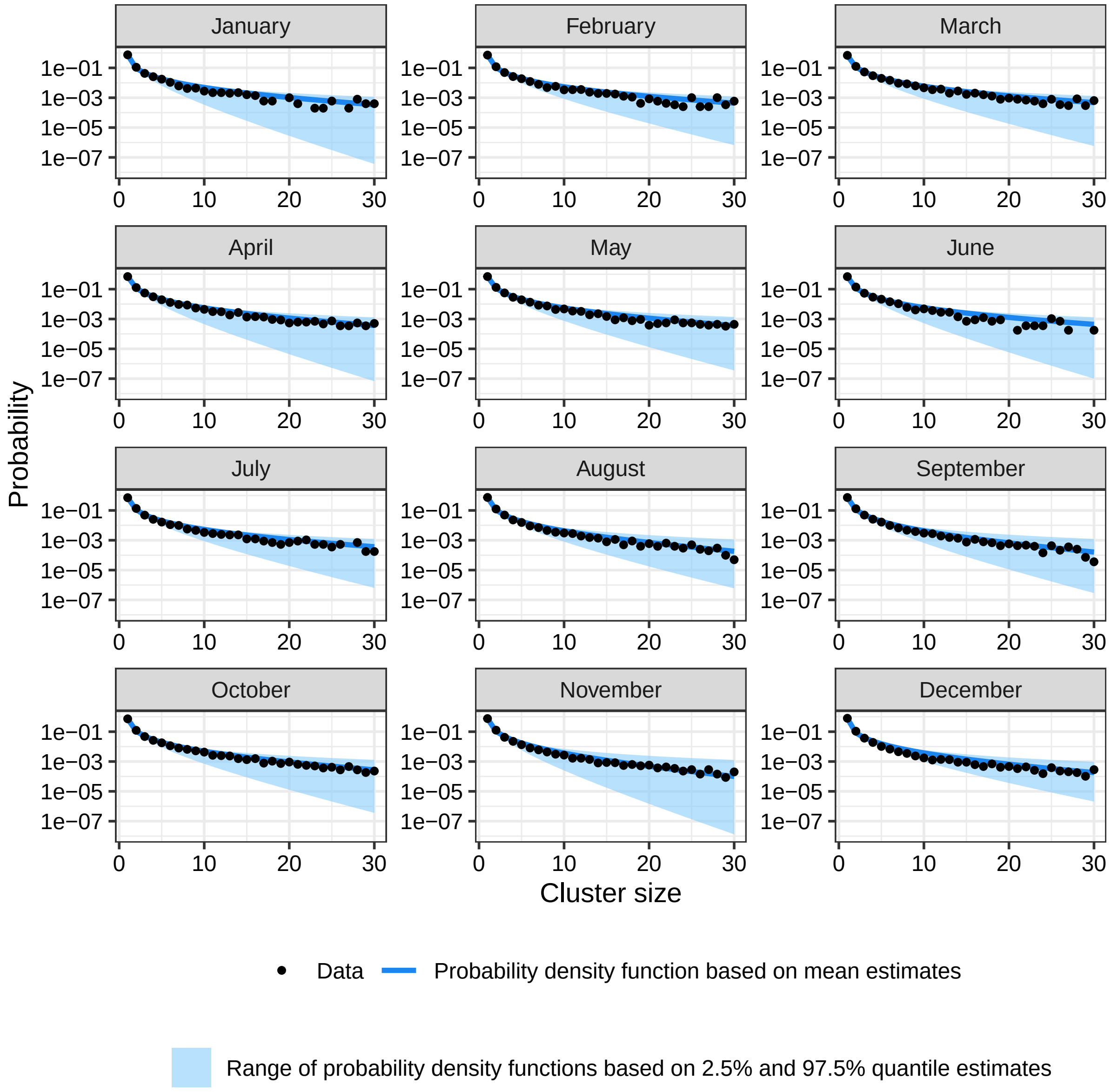

Fig P. Goodness of fit of cluster size distribution (Germany).

K Bibliography

1. Shu Y, McCauley J. GISAID: Global initiative on sharing all influenza data – from vision to reality. Eurosurveillance. 2017;22: 30494. doi:10.2807/1560-7917.ES.2017.22.13.30494
2. Federal Department of Home Affairs FDHA, Federal Office of Public Health, Prevention and Public Health Services Directorate, Communicable Diseases Division. Infectious diseases dashboard (IDD). 2024. Available: `https://idd.bag.admin.ch/portal-data`
3. Statens Serum Institut. Covid-19 - Danmark (region). 2024. Available: `https://experience.arcgis.com/experience/220fef27d07d438889d651cc2e00076c/page/Covid-19-Regionalt/`
4. Robert Koch-Institut. 7-Tage-Inzidenz der COVID-19-Fälle in Deutschland. 2024. doi:10.5281/zenodo.10819876
5. Blumberg S, Lloyd-Smith JO. Comparing methods for estimating R0 from the size distribution of subcritical transmission chains. Epidemics. 2013;5: 131–145. doi:10.1016/j.epidem.2013.05.002
6. Park Y, Martin MA, Koelle K. Epidemiological inference for emerging viruses using segregating sites. Nature Communications. 2023;14: 3105. doi:10.1038/s41467-023-38809-7
7. Neher RA. Contributions of adaptation and purifying selection to SARS-CoV-2 evolution. Virus Evolution. 2022;8: veac113. doi:10.1093/ve/veac113
8. Ganyani T, Kremer C, Chen D, Torneri A, Faes C, Wallinga J, et al. Estimating the generation interval for coronavirus disease (COVID-19) based on symptom onset data, March 2020. Eurosurveillance. 2020;25: 2000257. doi:10.2807/1560-7917.ES.2020.25.17.2000257
9. Blumberg S, Lloyd-Smith JO. Inference of R0 and Transmission Heterogeneity from the Size Distribution of Stuttering Chains. PLOS Computational Biology. 2013;9: e1002993. doi:10.1371/journal.pcbi.1002993
10. Nishiura H, Yan P, Sleeman CK, Mode CJ. Estimating the transmission potential of supercritical processes based on the final size distribution of minor outbreaks. Journal of Theoretical Biology. 2012;294: 48–55. doi:10.1016/j.jtbi.2011.10.039
11. Eales O, Haw D, Wang H, Atchison C, Ashby D, Cooke GS, et al. Dynamics of SARS-CoV-2 infection hospitalisation and infection fatality ratios over 23 months in England. PLOS Biology. 2023;21: e3002118. doi:10.1371/journal.pbio.3002118
12. Huisman JS, Scire J, Angst DC, Li J, Neher RA, Maathuis MH, et al. Estimation and worldwide monitoring of the effective reproductive number of SARS-CoV-2. Davenport MP, editor. eLife. 2022;11: e71345. doi:10.7554/eLife.71345
13. Tran-Kiem C, Bedford T. Estimating the reproduction number and transmission heterogeneity from the size distribution of clusters of identical pathogen sequences. Proceedings of the National Academy of Sciences. 2024;121: e2305299121. doi:10.1073/pnas.2305299121
14. Blab/size-genetic-clusters. Bedford Lab; 2024. Available: `https://github.com/blab/size-genetic-clusters`
