# Supplementary material for: Estimating Re and overdispersion in secondary cases from the size of identical sequence clusters of SARS-CoV-2
Source: PLoS Comput Biol. 2025 Apr 15;21(4):e1012960. doi: 10.1371/journal.pcbi.1012960 (PMC12040226; doi:10.1371/journal.pcbi.1012960)
Supplement: S3 Table — (PDF) [file pcbi.1012960.s004.pdf]

SUPPLEMENTAL TABLE

Data Availability

GISAID Identifier: EPI_SET_240326uh
doi: 10.55876/gis8.240326uh

All genome sequences and associated metadata in this dataset are published in GISAID's EpiCoV database. To view the contributors of each individual sequence with details such as accession number, Virus name, Collection date, Originating Lab and Submitting Lab and the list of Authors, visit 10.55876/gis8.240326uh

Data Snapshot

- EPI_SET_240326uh is composed of 355,192 individual genome sequences.
- The collection dates range from 2020-02-03 to 2022-11-24;
- Data were collected in 3 countries and territories;
- All sequences in this dataset are compared relative to hCoV-19/Wuhan/WIV04/2019 (WIV04), the official reference sequence employed by GISAID (EPI_ISL_402124). Learn more at https://gisaid.org/WIV04.
