# Supplementary material for: Estimating Re and overdispersion in secondary cases from the size of identical sequence clusters of SARS-CoV-2
Source: PLoS Comput Biol. 2025 Apr 15;21(4):e1012960. doi: 10.1371/journal.pcbi.1012960 (PMC12040226; doi:10.1371/journal.pcbi.1012960)
Supplement: Attachment — Submitted filename: 062224 Review.pdf [file pcbi.1012960.s005.pdf]

In this manuscript, Hodcroft et al. develop a Bayesian inference framework for estimating the reproduction number, dispersion parameter and testing probability for SARS-Cov transmission from sequence data. For each month of data, the size distribution of clusters of identical sequences are used to estimate the 'genomic' reproduction number and dispersion parameter. The genomic reproduction number is than transformed into an effective reproduction number based on the mutation rate and generation time. This method is applied to Switzerland, Denmark, and Germany from January to December 2021. The analysis suggests that Denmark had less superspreading than Switzerland or Germany.

Given the increased availability of sequencing data, the types of analyses presented in the manuscript are increasingly relevant. My biggest concern is that the likelihood calculations that the paper presents has already been published by Tran-Kiem and Bedford [Ref 39]. So I think more effort is needed to distinguish this manuscript from existing literature.

Major concerns:

- As mentioned within the manuscript, the inference method is predicated on the assumption that R_g is less than. In practice that means that there must be few clusters that have ongoing transmission to successive months. Can this assumption be validated based on what proportion of clusters extend beyond one month?
- In figure 4 and S3 there are notable time periods where the R estimates from sequences and case counts differ markedly. While the manuscript does a nice job in the discussion of explaining potential reasons why this may have occurred, there is little quantitative exploration for how various modifications to the modeling assumptions would impact the inference of R_e. However, I think this is also an opportunity, because I think a more in-depth exploration of biases of the estimation technique would effectively distinguish this paper from Ref 39.
- On p.11, it appears that the estimates of the dispersion parameter are assumed to be more reliable than the estimates of R_e. To my mind, it is unclear why estimates of the dispersion parameter should be trusted more when there isn't a clear understanding of why estimates of the effective reproduction number differ depending on whether it is estimated from cases or genomic sequences. I think more exploration and discussion about the potential biases of k estimation are needed.

Additional thoughts (page numbers refer to the # noted in the lower right corner of the pdf):

- P.4: I am not familiar with the methods used to cluster genomes. So I was confused by the explanation that only sequences with more than four mutations are used. Does that only refer to comparisons to the original consensus sequence? Or does it mean that within a cluster, sequences can differ by up to four mutations. If the

latter, how do we know that clusters are not over-aggregated. Can you explain a bit more?

- P.6. 'the number of secondary cases that belong to the same identical sequence cluster as their source case also follows a negative binomial distribution with mean R_g and dispersion parameter k.'. Naively, I would have assumed that the coefficient of variation for the full distribution and with-in sequence distribution of secondary cases is the same. So I would think the k for the with-in sequence distribution needs to be updated because the coefficient of variation of a negative binomial distribution depends on both R and k, and not just k. However, the derivation in the supplement is noted – so perhaps reference that derivation and/or provide a more intuitive explanation for why k does not change?
- Table S1: I'm not sure why the number of sampled sequences (Nos) is less than the number of sequences in clusters (Nosic). Can you clarify the definitions
- Fig 2: It's a bit hard to comprehend the errors for the dispersion parameter. Consider using a more interpretable metric for computing RMSE – such as std. deviation, or coefficient of variance.
- Fig 4: Naively, the estimates of testing probability seem quite high, particularly given the moderate proportion of asymptomatic cases. Are these estimates aligned with other published studies?
- Consider adding a comment about how importation and/or expectation of cases effects parameter estimates.
- Consider adding a comment on how contact-tracing efforts (or even natural clustering of testing due to human behavior) effects parameter estimaton. I.e., each case is assumed to be tested and sequenced with equal probability. What happens if this probability varies by cluster? How will this bias the inferred values of R and k?
