# Supplementary material for: Estimating Re and overdispersion in secondary cases from the size of identical sequence clusters of SARS-CoV-2
Source: PLoS Comput Biol. 2025 Apr 15;21(4):e1012960. doi: 10.1371/journal.pcbi.1012960 (PMC12040226; doi:10.1371/journal.pcbi.1012960)
Supplement: Attachment — Submitted filename: responseToReviews_Randk.pdf [file pcbi.1012960.s007.pdf]

Dear Konstantin Blyuss and Hannah Clapham,

We would like to thank you for the opportunity to resubmit our manuscript, "Estimating R_e and overdispersion in secondary cases from the size of identical sequence clusters of SARS-CoV-2", to PLoS Computational Biology. We greatly appreciate both the thoughtful reviews we received and the editor feedback, both of which we took onboard fully and have addressed in the revised version of the manuscript we submit today. In particular, we appreciated the opportunity to more fully demonstrate how our manuscript differs from Tran-Kiem and Bedford's work, and to modify the estimate of the mean number of mutations per transmission we use in the model (both of which are explained more fully below).

Please note that in the changes below, we reference line numbers in the *tracked changes* version of the manuscript to make it easier for the reviewers and editors to see the change in question.

During the original submission, we did not find a clear way to specify within the PLoS system that Dr Hodcroft and Mr Wohlfender are co-first authors, though this was included in the PDF. We'd greatly appreciate if there would be a way to adjust this so that it's recorded appropriately in the system.

Once again, we thank you for this opportunity to re-submit our work, which we believe is greatly improved by the feedback we've received, and thank the editors and reviewers in advance for their additional time.

Sincerely,
Emma B Hodcroft, on behalf of all authors
emma.hodcroft@unibas.ch

Editor comment:

As with all papers reviewed by the journal, your manuscript was reviewed by members of the editorial board and by several independent reviewers. In light of the reviews (below this email), we would like to invite the resubmission of a significantly-revised version that takes into account the reviewers' comments.

Two of the three Reviewers have raised a point about the results presented in this manuscript being too close or identical to the work published in a recent paper of Tran-Kiem and Bedford (https://www.pnas.org/doi/10.1073/pnas.2305299121). It is essential that the authors clearly indicate both in the revised version of the manuscript, and in their responses the novelty of their method and/or results in relation to that work. The Reviewers have also made a number of other suggestions on how to improve the paper and make the presentation clearer, so I suggest the authors carefully go through them and address them.

Response:

We thank the editor for the feedback. We have carefully read all comments of the three reviewers and have made improvements to our manuscript.

In the discussion of the revised manuscript (pages 11-12, lines 307-324), we have highlighted the conceptually novel aspect of our study, that identical sequences can efficiently inform epidemiological parameter inference even if the data sets are too large to allow phylodynamic analysis:

"During our work, Tran-Kiem and Bedford (2024) [39] published a study based on the same underlying theory as ours. The fact that two research groups applied the same concepts at the same time independently of each other to estimate parameters related to transmission dynamics of infectious diseases is certainly linked to the increasing availability of viral genomic data, and underlines the promising nature of this approach. A major difference between the two studies is the choice of the method for parameter inference. Instead of a maximum likelihood approach, we have decided to use a Bayesian framework allowing the full propagation of the uncertainty related to data and incompletely known input parameters throughout the estimation process. Furthermore, while Tran-Kiem and Bedford (2024) [39] applied their model to rather small datasets of different diseases where traditional phylodynamic methods would also have been applicable, we have dealt with significantly larger country-wide datasets covering the whole year 2021. We have developed approaches to efficiently identify clusters of identical sequences within these datasets containing hundreds of thousands of sequences and applied our model to monthly segments of the data to estimate the effective reproduction number R_e and the dispersion parameter k and track their change over the year 2021. These points demonstrate the different applications of the underlying theory and the complementary nature of these two studies."

Following the comments of several reviewers about the way we included the mutation process into our model of the size distribution of identical sequence clusters, we have reparameterized the model to use an estimate of the mean number of mutations per transmission to inform the mutation probability μ, instead of determining μ based on estimates of the yearly mutation rate M and the mean generation interval D. One reviewer highlighted that estimates of M are impacted by purifying selection (Ghafari et al. MBE 2022) and that the generation interval similarly can change substantially over time because of control efforts (Ali et al. Science 2020) or because of viral differences (e.g., generation interval differences by variant of concern). We are convinced that the new method used is a more direct and reliable way to inform μ because the estimate of the mean number of mutations per transmission is obtained directly from the comparison of the genome sequences of a set of transmission pairs.

This led us to updating all results of parameter estimations presented in the manuscript. Since the mutation probability is higher in the new version of the model, the estimates of the effective reproduction number R_e are higher as well. The estimates of the dispersion parameter and the testing probability remain almost identical, however. We have moved the results of parameter estimations obtained with the version of our model presented in the original submission to section 7.4 of the Supplementary Material.

Reviewer #1:

In this manuscript, Hodcroft et al. develop a Bayesian inference framework for estimating the reproduction number, dispersion parameter and testing probability for SARS-Cov transmission from sequence data. For each month of data, the size distribution of clusters of identical sequences are used to estimate the 'genomic' reproduction number and dispersion parameter. The genomic reproduction number is than transformed into an effective reproduction number based on the mutation rate and generation time. This method is applied to Switzerland, Denmark, and Germany from January to December 2021. The analysis suggests that Denmark had less superspreading than Switzerland or Germany.

Given the increased availability of sequencing data, the types of analyses presented in the manuscript are increasingly relevant. My biggest concern is that the likelihood calculations that the paper presents has already been published by Tran-Kiem and Bedford [Ref 39]. So I think more effort is needed to distinguish this manuscript from existing literature.

Response:
We thank the reviewer for their thoughtful response and insightful questions and suggestions. Regarding the similarity with the work of Tran-Kiem and Bedford, please see our response to the editor addressing this point above.

Major concerns:

1. *As mentioned within the manuscript, the inference method is predicated on the assumption that R_g is less than 1. In practice that means that there must be few clusters that have ongoing transmission to successive months. Can this assumption be validated based on what proportion of clusters extend beyond one month?*

Response:
We thank the reviewer for this interesting question.

The reviewer is correct that our estimation framework is based on the assumption that R_g is strictly less than 1. As R_g corresponds to the average number of secondary cases generated by a case with the same viral genomic sequence, a value of $R_g > 1$ would imply that there is a non-zero probability that identical sequence clusters grow to infinite size. This means that the value of R_g would become unidentifiable with our inference framework. Still, R_g represents only an average, and stochastic events may still lead to large cluster sizes. In our framework, this phenomenon is measured by the parameter k. The reviewer is correct that large clusters may span over several months, so that the same phenomenon could also be measured by the proportion of clusters extending beyond 1 month. We have added these proportions in the results section (page 9, lines 248-254) to show that the proportion of clusters extending beyond one month is lower than 10%.

"Each identical sequence cluster was assigned to a given month if it contained at least one sequence from that month, thus we assigned 3603 (6.1%) identical sequence clusters from Switzerland, 6610 (7.8%) from Denmark and 14,520 (6.6%) from Germany to at least two months of 2021. Including the identical sequence clusters overlapping from 2020 into 2021 or from 2021 into 2022, the number of identical sequence clusters extending beyond one month among the identified clusters was 4151 (7.1%) in Switzerland, 8144 (9.6%) in Denmark and 15,992 (7.3%) in Germany."

2. *In figure 4 and S3 there are notable time periods where the R estimates from sequences and case counts differ markedly. While the manuscript does a nice job in the discussion of explaining potential reasons why this may have occurred, there is little quantitative exploration for how various modifications to the modeling assumptions would impact the inference of R_e. However, I think this is also an opportunity, because I think a more in-depth exploration of biases of the estimation technique would effectively distinguish this paper from Ref 39.*

Response:
We thank the reviewer for this suggestion on how to improve our manuscript. We took this opportunity to extend our exploration of the parametrization of our model, which led to changing the manner the mutation probability is included into our model of the size distribution of identical sequence clusters, please see our response to the editor addressing this point.

Chapter 7 of the supplementary material now contains an expanded sensitivity analysis of our model presenting the results obtained with the modified models. We have considered five modified models that supplement the model presented in the manuscript to cover all six combinations of three assumptions on the mutation probability (prior distribution for mutation probability, constant value for mutation probability or prior distribution for yearly mutation rate) and two assumptions on the testing probability (prior distribution or constant value).

We see that our results are robust to these assumptions. As expected, the estimates for R_e have been scaled up when assuming a higher mutation probability. However, the estimates of k and the testing probability are almost identical compared to the original model.

We now refer to the collection of alternative models in our discussion (page 13, lines 383-385):

"For further exploration of the impact of different model assumptions on R_e estimates, see Supplementary Material S1 Text, chapter 7."

3. *On p.11, it appears that the estimates of the dispersion parameter are assumed to be more reliable than the estimates of R_e. To my mind, it is unclear why estimates of the dispersion parameter should be trusted more when there isn't a clear understanding of why estimates of the effective reproduction number differ depending on whether it is*

estimated from cases or genomic sequences. I think more exploration and discussion about the potential biases of k estimation are needed.

Response:
We thank the reviewer for this excellent comment and suggestion to explore this more in the manuscript. In short, since *k* is expected to vary less over time than R_e, our assumption that values are constant within our one-month time windows is less restrictive to the estimation of these variables. We have added a few sentences to the discussion section (page 13, lines 387-394) of our manuscript to explore this aspect a bit further:

"While R_e is constantly changing by definition, for example due to increasing immunity in the population, *k*, which reflects the degree of individual variation [52], is more stable over time. Still, *k* can vary, for example depending on the variant of SARS-CoV-2 or the implementation of public health measures. Therefore, our assumption of constant values of R_e and *k* within monthly time windows is less restrictive for the estimation of *k* than for R_e, which makes our framework more suitable for the estimation of *k* than for the estimation of R_e."

Additional thoughts (page numbers refer to the # noted in the lower right corner of the pdf):

1. *P.4: I am not familiar with the methods used to cluster genomes. So I was confused by the explanation that only sequences with more than four mutations are used. Does that only refer to comparisons to the original consensus sequence? Or does it mean that within a cluster, sequences can differ by up to four mutations. If the latter, how do we know that clusters are not over-aggregated. Can you explain a bit more?*

Response:
We thank the reviewer for pointing out that this was not as clear as it could be. This limitation was only applied to the starting set of those mutations used for the analysis as a whole, and the four mutations are indeed relative to the original Wuhan reference. This exclusion was done to avoid clustering sequences in the very early pandemic where identical mutations appeared independently on the Wuhan background. However as we used sequences from January to December 2021 (when circulating variants generally had more than 4 mutations), this had little impact on our analysis. We have clarified this on page 4, lines 90-96 in the introduction:

"To minimize the chance that sequences with low numbers of mutations, which may have arisen independently, falsely cluster together, only sequences with more than four mutations, relative to the reference Wuhan strain, were used to form clusters. Due to the mutation rate of SARS-CoV-2, this limitation only excluded sequences at the very start of the pandemic and before our study period of 2021, as most lineages had more than four nucleotide mutations by March/April 2020."

2. *P.6. ‘the number of secondary cases that belong to the same identical sequence cluster as their source case also follows a negative binomial distribution with mean R_g and dispersion parameter k.’. Naively, I would have assumed that the coefficient of variation for the full distribution and with-in sequence distribution of secondary cases is the same. So I would think the k for the with-in sequence distribution needs to be updated because the coefficient of variation of a negative binomial distribution depends on both R and k, and not just k. However, the derivation in the supplement is noted – so perhaps reference that derivation and/or provide a more intuitive explanation for why k does not change?*

Response:
We thank the reviewer for pointing out that our derivation in the Supplementary Material was not made clearly visible to readers. We have added an introduction to chapter 2 of the Supplementary Material to provide more context to the mathematical work:

“In this chapter, we present a detailed mathematical derivation of a statement upon which our model of the size distribution of identical sequence clusters is built: If the number of secondary cases created by an infected individual follows a negative binomial distribution with mean R_e and dispersion parameter k, then the number of secondary cases that belong to the same identical sequence cluster as their source case also follows a negative binomial distribution with mean $R_g = (1 - \mu) R_e$, where μ is the probability of a mutation occurring in an infected individual, and dispersion parameter k.

First, we present this result in a mathematical context. Its application within our model of the size distribution of identical sequence clusters will be explained in the next chapter. We start by proofing two auxiliary results that we will use within the proof of our main statement.”

In addition, on page 6, lines 188-189 of the manuscript, we have added a reference to the derivation of the statement on how the mean and the dispersion parameter change between the complete distribution and the with-in sequence distribution of secondary cases:

“We have included a complete mathematical derivation of this property in the Supplementary Material S1 Text, chapter 2.”

3. *Table S1: I’m not sure why the number of sampled sequences (Nos) is less than the number of sequences in clusters (Nosic). Can you clarify the definitions*

Response:
We thank the reviewer for pointing out that this definition is not formulated in sufficient detail. We have extended the paragraph before table S1 of the Supplementary Material (page 3):

[Existing text] “A cluster is assigned to a month if there is a sequence in the cluster that has been sampled in the month. The same cluster can therefore be assigned to several months.”

[New text] “This is why the clusters assigned to a month contain strictly more sequences than sequences were sampled during the respective month.” [Existing text] “Furthermore, we retrieved daily numbers of newly confirmed cases from public sources of Switzerland [2], Denmark [3] and Germany [4] and summarized them to monthly values.” [New text] “The number of confirmed cases, the number of sampled sequences, the number of assigned identical sequence clusters, the number of sequences contained in the assigned identical sequence clusters, the sequencing coverage and the size of the largest assigned identical sequence cluster by country and month are presented in the following tables.”

4. *Fig 2: It’s a bit hard to comprehend the errors for the dispersion parameter. Consider using a more interpretable metric for computing RMSE – such as std. deviation, or coefficient of variance.*

Response:
We are aware that the interpretation of the RMSE is not straightforward, and thank the reviewer for this suggestion. We have already included a variant of this plot using another error metric, the coverage of the true value by the 95% credibility interval of the posterior distribution, in the supplementary material of our original submission (Figure S4). Furthermore, we have reworked Figure 2 to include a more readily interpretable display of the error, by using the mean and 95% credible interval of the pooled samples of the posterior distribution. We have moved the original figure using RMSE to section 5.2 of the supplementary material as Figure S3.

5. *Fig 4: Naively, the estimates of testing probability seem quite high, particularly given the moderate proportion of asymptomatic cases. Are these estimates aligned with other published studies?*

Response:
We thank the reviewer for this observation. Indeed our estimates of testing probability are high, as we note at the end of the Results section, and higher than estimates of case ascertainment from England (ref 51), which estimated around 57% ascertainment for 2021. However, it should be noted that the uncertainty on this parameter remains very high, highlighting the difficulty of estimating such quantities with these data. The main focus of our study was on the estimation of R_e and k, but including the testing probability does have some advantages, which we now include in the manuscript (page 7, lines 203-207):

“While the main focus was to estimate R_e and k, inclusion of the testing probability allows for our approach to be generalizable to contexts where few data exist about this quantity. In addition, this allows incorporating the uncertainty about testing in the final estimates of R_e and k.”

Additionally, when we fixed the testing probability to the estimated ascertainment in England, we found this had only a small impact on our estimates of R_e and k (Supplementary Material, sections 7.1, 7.3 and 7.5).

6. *Consider adding a comment about how importation and/or expectation of cases effects parameter estimates.*

Response:
We thank the reviewer for this stimulating comment. Though we cannot estimate importation/exportation with every country globally, we investigated this briefly by looking for clusters with identical sets of mutations found in more than 1 of our focal countries. We found that between 0.7-3.1% of clusters in one country were found in another country, indicating possible importation/exportation. As expected, the majority of these were singletons and small clusters. 370 clusters were found in all 3 countries, a very small proportion of the tens of thousands to hundreds of thousands of clusters we observe within each country. We have added a paragraph to the discussion of our manuscript (pages 13-14, lines 411 - 429) to explain the potential impact of imported and exported cases on the parameter estimation:

"Another potential source of bias of the estimates of both R_e and k is the import and export of cases. An imported case from abroad most likely carries a mutation not yet present in the country and hence starts a new cluster of identical sequences. Imported cases could on one hand create fewer secondary cases compared to local cases because their infectious period already might have started prior to arrival. Tsang et al. [53] took this into account in their study by adjusting the infectiousness profiles of imported cases. Furthermore, the imposition of quarantine measures was likely effective in reducing transmission [54]. On the other hand Creswell et al. [55] argue that frequent travelers might have more contacts than those individuals who do not travel abroad. This would lead to a higher risk of transmission. An exported case leads to the corresponding identical sequence cluster not getting as big as it would in a scenario without exportation of cases. Therefore, in general the importation and exportation of cases could increase the overall number of identical sequence clusters and reduce the mean size of identical sequence clusters. Such a deformation of the size distribution of identical sequence clusters could lead to an underestimation of both the effective reproduction number and the dispersion parameter. However, we expect that the proportion of imported, respectively exported, cases in the dataset is small. Therefore, we assume that our results are not significantly influenced by case importation and exportation."

7. *Consider adding a comment on how contact-tracing efforts (or even natural clustering of testing due to human behavior) effects parameter estimation. I.e., each case is assumed to be tested and sequenced with equal probability. What happens if this probability varies by cluster? How will this bias the inferred values of R and k?*

Response:
We thank the reviewer for this excellent remark. At the start of the analysis we tried to include a testing probability depending on the cluster size into our model, but refrained from this due to computational difficulties. We have expanded the description of this aspect in the discussion section of our manuscript (page 12, lines 347-360):

[Existing text] “Third, we assumed an independent observation process, i.e., infected cases are detected randomly and tested cases are sequenced at random. However, testing and sequencing uptake might cluster in certain settings, such as during contact tracing and outbreak investigations.” [New text] “This would on average lead to a higher detection probability for cases contained in a large identical sequence cluster and to a lower detection probability for cases contained in a small identical sequence cluster. We think that the effects on the estimations of R_e and k resulting from this are limited. For example, a lower probability of observing an identical sequence cluster of size one would indicate a larger value of k, whereas a higher probability of observing large clusters would indicate a lower value of k. However, it is possible that the assumption of an independent observation process that we took to limit computational complexity of the model is not perfectly realistic, which manifests in large credible intervals for the estimates of the testing probability.”

Reviewer #2:

I think this is a great, useful idea, that is well-explained. I don't have much in the way of criticisms or suggestions.

Response:
We thank the reviewer for the positive overall assessment of our manuscript.

Some small points:

1. *From text on page 5, and also in Section 3, it was not 100% clear to me that when a mutation occurs at a node, then it is assumed that the node *has* that mutation. This is clear from the figure on page 5. There is an assumption built in here which is that any mutation occurs early in the life of the pathogen within one host, both before anyone else is infected and also before the host is sampled. I think this is a good assumption, but you certainly could imagine situations where it is violated.*

Response:
We appreciate the reviewer’s comment that the description of the assumptions underlying the mutation process should be more detailed. We have added a few words to both respective paragraphs in the manuscript (page 5, lines 142-150):

“Provided a mutation took place, we assumed that it happened prior to the respective node further transmitting the virus or being detected. Consequently, a new mutation was passed on to all secondary cases. This assumption is backed by the work of Braun et al. [46], which showed that overall within-host diversity is low during acute infection and estimated that transmission bottlenecks between hosts are narrow. Furthermore, they argued that the low number of viral

particles transmitted while infecting another person can induce a founder effect that wipes out low-frequency intra-host single nucleotide variants.”

and the Supplementary Material (page 12:

“[Existing text] We assume that at each node a mutation occurs independently of all other nodes” [New text] “and prior to the respective node transmitting the virus to secondary cases or being detected. Therefore, if a” [Existing text] “mutation happens at a node, all direct offspring of that particular node receive the mutation.”

Furthermore, we have updated figure 1 on page 6 of our manuscript to convey this more clearly.

2. *As the authors acknowledge, the error bars on the estimates of R_e are suspiciously small. Part of the issue is that the model assumes things that aren't true (like R_e being constant over the course of a month). But I wondered about D and \tau_sequence being fixed parameters. We know that D can change with conditions in a pandemic, and even if it didn't, I don't think we know it with high precision.*

Response:
We thank the reviewer for their comment. The assumption that R_e is constant during a certain time period is necessary to be able to determine the cluster size distribution from the secondary case distribution with our approach. We opted for months in order to find a compromise between a certain time span that covers the entire activity period of a large number of clusters and a certain number of time periods that make it possible to observe changes over time.

The reviewer is right pointing out that there is some uncertainty around the estimated value of *D*. We have developed models incorporating the mutation process in an alternative way (based on an estimate of the mutation probability from transmission pairs) and now present this reparameterized model in the manuscript. We have included the results of parameter estimations with the original version of our model and with several other modified versions in chapter 7 (“Sensitivity analysis”) of the Supplementary Material (main model of original submission in section 7.4). As expected, the estimates for R_e in the main text have been scaled up due to assuming a higher mutation probability. *k* and the testing probability are not much different compared to the original model. We conclude from these results that the uncertainty around the estimate of *D*, which simply scales the mutation process, has no substantial impact on the estimated parameters.

3. *In section 4 of the SI, I didn't understand the table entry for \tau_sequence "1.42 − 85.94%". Also, I was curious about the scaled beta for \tau_test. How did expert knowledge lead to a distribution where it's sharply peaked above 0.05 but zero below it? It may be some Bayesian thing I don't understand.*

Response:

We thank the reviewer for this point and acknowledge that we have not sufficiently explained our reasoning of defining the prior distribution for τ_{test} on [0.05, 1] instead of [0, 1].

We have added a few sentences to the first paragraph of chapter 4 ("Prior distributions and fixed parameters*) of the Supplementary Material (page 15) to clarify this aspect:

[Existing text] "We scaled a beta distribution, usually defined on the interval [0, 1] or]0, 1[, to the interval [0.05, 1]." [New text] "The reason for applying this transformation is to control the computational cost of our inference model. The lower τ_{test}, the more summands are required to reliably approximate *P(Y = j)*, see chapter "Model of cluster size distribution" for the definition of *Y*. Since we are convinced that at least 5% of all COVID-19 cases were confirmed by a test at any time in 2021 in Switzerland, Denmark and Germany, we have shifted the lower limit of the domain of the prior distribution of τ_{test} from 0 to 0.05."

Concerning the entry of $\tau_{sequence}$ in table S7B in the Supplementary Material: This range refers to the minimal and maximal value of the combined columns Nos/Nocc of tables S1, S3 and S5. We have computed the sequencing probability as the ratio of the number of viral genome sequences sampled during a month in a certain country to the number of confirmed cases during the same month in the same country.

4. *page 4. "To minimize the chance... only sequences with more than four mutations were used." I would have appreciated "four mutations with respect to the reference genome" or something like that.*

Response:
We thank the reviewer for catching that this was not as clear as it could be, and have added their suggestion on page 4, lines 90-96 in the manuscript:

"To minimize the chance that sequences with low numbers of mutations, which may have arisen independently, falsely cluster together, only sequences with more than four mutations, relative to the reference Wuhan strain, were used to form clusters. Due to the mutation rate of SARS-CoV-2, this limitation only excluded sequences at the very start of the pandemic and before our study period, as most lineages had more than four nucleotide mutations by March/April 2020."

Even smaller points:

- *page 3, line 48. The first two sentences of the paragraph start with the same phrase. Also, is the pandemic still ongoing?*

Response:
We thank the reviewer for spotting this issue. We have rephrased the sentence (page 3, lines 49-53):

“During a pandemic, individual transmission chains might stop, but the state of being in a pandemic implies that overall these chains persist. Therefore, cases can not be readily decomposed into individual limited transmission chains.”

- *page 8, fig 2, thin out the y axis labels so they are not overlapping?*

Response:
We thank the reviewer for bringing this to our attention. Based on feedback of another reviewer we have substantially changed the way we present the results of the simulation study and therefore created a new Fig 2.

- *page 12, line 320, "events may *be* favoured" ?*

Response:
We thank the reviewer for having read our manuscript so carefully. We have corrected this (page 13, lines 409-410):

“Another potential explanation is that superspreading events may be favoured by indoor gathering during winter periods.”

Reviewer #3:

In this manuscript, Hodcroft and colleagues propose to use sets of identical viral sequences (readily available from public databases) to estimate the time-varying effective reproduction number R_e and, more critically, the overdispersion parameter k of the negative binomial distribution that quantifies the extent of transmission heterogeneity. The overall idea of the manuscript is interesting, as case data alone are not very informative of the extent of transmission heterogeneity, and studies of detailed outbreaks are few. As such, approaches such as the one proposed show great promise to getting a handle on the extent of viral superspreading that occurs and how this changes over time. I do, however, have some major concerns with the manuscript as is and I hope that my recommendations below prove helpful.

Major concerns/suggestions:

1. *The authors briefly mention the published work of Tran-Kiem and Bedford (PNAS) on lines 59-60. How does the method in that published paper compare to the current method? The authors indicate that this is a related study using the same approach. In the discussion, they then say it is a similar approach. Is it exactly the same approach, so if the authors used their approach on the same dataset from New Zealand (line 313), would they estimate the same k of 0.63, or how do the approaches differ? More detailed*

information on this would be very helpful, especially to gauge the novelty of the currently submitted work.

Response:
We thank the reviewer for this suggestion. This aspect has also been brought up by other reviewers, and a full response can also be seen in our reply to the editor's comments. We have applied our model to the dataset from New Zealand and have obtained similar results as Tran-Kiem and Bedford. We have included our results in the Supplementary Material in chapter 8 ("Comparison to literature").

2. *Equation (1) for mu: instead of using the estimated substitution rate (M) and the generation interval (D set to 5.2 days) to estimate mu = 0.181, I recommend using the quantified probability of mu from established transmission pairs. This is a more relevant data point, given that estimates of M are impacted by purifying selection (Ghafari et al. MBE 2022) and the generation interval similarly can change substantially over time because of control efforts (see, e.g., Ali et al. Science 2020) or because of viral differences (e.g., generation interval differences by VOC). Park et al. Nature Communications (2023) provide an empirical estimate of the mean number of mutations that occur at transmission of mu = 0.33 (95% CI = 0.22-0.48; their Fig 6C) from transmission pair data. This is the mean of a fit Poisson distribution. Therefore, the probability of 0 mutations at transmission is given by exp(-mu) = 0.7189, which is ballpark similar to Hodcroft et al.'s (1-0.181) = 0.8190, but the CI based on the empirical transmission pairs yields a range between 0.6188 to 0.8025, which does not include 0.8190.*

Response:
We thank the reviewer for pointing out the direct measurements of mu from transmission pairs and we agree that these data are more directly applicable to our use case. We have modified our model and run it with the suggested values, which we now use in the main text. The results of our previous estimations (using the mutation probability μ = 18.1%) are now included in the Supplementary Material, section 7.4 (in chapter 7 "Sensitivity analysis").

We have added additional explanation around the two possible mutation rates, and the decision to use the higher one suggested by the reviewer in the manuscript.

Page 5, lines 164-171:

"The mean number of mutations per transmission has been estimated to be M_T = 0.33 [47]. Based on M_T we can determine the probability μ that genomes from two subsequent cases in a transmission chain differ from each other by at least one mutation:

$$\mu = 1 - e^{-M_T}$$

Plugging in M_T = 0.33 into the above formula, we get μ = 28.1%. Alternatively, we estimated the probability that two such genomes are not identical from the evolutionary rate M and the serial interval D:"

And pages 5-6, lines 174-180:
"While the former value for μ might be a slight over-estimate since it is not clear whether all transmission pairs used in this estimation are direct transmission pairs, the latter is likely an under-estimate since deleterious mutations contribute more on short time scales within transmission clusters than on longer time scales used to estimate the rate M. We use μ = 28.1% in the main text and provide an alternative analysis with μ = 18.1% in the supplementary materials (Supplementary Material S1 Text, chapter 7)."

3. *The authors indicate that if the number of offspring is negative binomially distributed with mean R_e and overdispersion parameter k, then the number of viral offspring that are genetically identical to the focal individual is similarly negative binomially distributed with mean R_e(1-mu) and overdispersion parameter k. By the same reasoning, this would indicate that the number of viral offspring that are genetically dissimilar to the focal individual would be distributed with mean R_e(mu) and overdispersion parameter k. If this is the case, is the sum of two random variables drawn from these distributions negative binomially distributed with mean R_e and overdispersion parameter k?*

Response:
We thank the reviewer for sharing these thoughts with us. The reviewer is completely right that the number of secondary cases that are not genetically identical to the source case follows a negative binomial distribution with mean $\mu * R_e$ and overdispersion parameter k.

In general, the sum of a random variable following a negative binomial distribution with mean $\mu * R_e$ and overdispersion parameter k and a random variable following a negative binomial distribution with mean $(1 - \mu) * R_e$ and overdispersion parameter k does not necessarily follow a negative binomial distribution with mean R_e and overdispersion parameter k. If the two random variables are stochastically independent, then the sum follows a negative binomial distribution with mean R_e and overdispersion parameter k if and only if μ = 0.5.

In our setup, the number of secondary cases that are genetically identical to the source case and the number of secondary cases that are not genetically identical to the source case are not stochastically independent.

4. *Supplemental methods: Section 2: probability theory: the structure of this could be improved upon, at least by adding an introduction as to why the specific lemmas and theorems are being provided to the reader. Also (obviously minor): Table S7A:]…[should be […].*

Response:
We thank the reviewer for this suggestion. We agree that this section needs some additional description and explanation of its purpose. Therefore we have added an introductory paragraph to chapter 2 of the Supplementary Material (page 9):

"In this chapter, we present a detailed mathematical derivation of a statement upon which our model of the size distribution of identical sequence clusters is built: If the number of secondary cases created by an infected individual follows a negative binomial distribution with mean R_e and dispersion parameter k, then the number of secondary cases that belong to the same identical sequence cluster as their source case also follows a negative binomial distribution with mean $R_g = (1-\mu) R_e$, where μ is the probability of a mutation occurring in an infected individual, and dispersion parameter k.

First, we present this result in a mathematical context. Its application within our model of the size distribution of identical sequence clusters will be explained in the next chapter. We start by proofing two auxiliary results that we will use within the proof of our main statement."

Concerning the reviewer's comment about the brackets in Table S7A, we are grateful to the reviewer for having studied our manuscript so carefully. Concerning the brackets, we use square brackets opening to the outer side] [to denote an interval excluding the boundary points and square brackets opening to the inner side [ ] to denote an interval including the boundary points. The gamma distribution is defined on the positive real axis. This is why we write]0, ∞[to emphasize that all real numbers greater than zero are included in the interval, but 0 and infinity are not included. For analogous reasons, we write]-∞, ∞[in the case of the normal distribution.

5. *Figure 1: I was initially confused by this figure, because the two solid blue colored nodes do not need to be a transmission pair. There could be multiple individuals between them that are all unsampled. Perhaps instead show a transmission tree, highlighting sampled individuals?*

Response:
We acknowledge that there is indeed potential to improve the clarity of the figure. Therefore we have reworked the figure (page 6) and changed the caption:

"Fig 1. Transmission chain and identical sequence clusters
The branching process model creates transmission chains. Viral mutation splits the transmission chains into identical sequence clusters. The numbers indicate the size of the observed identical sequence clusters, i.e. the number of cases in the clusters that were tested and sequenced."

This way, the plot better fulfills its purpose, illustrating the division of a transmission chain into clusters of identical sequences.

6. *Figure 2: This figure is meant to show how the method does on simulated data, however, it's very difficult to interpret the results from these RMSE plots, and I highly recommend revising the figure. For example, could you plot estimated versus true R_e, estimated versus true k, estimated versus true testing probability in some way where the readers could see how close the estimates are to their true values and if estimates are biased in one direction of the other? From Figure 2 I have a very hard time interpreting whether this method does a good job or a terrible job.*

Response:
We thank the reviewer for this suggestion. A similar comment was raised by another reviewer. Indeed, the RMSE does not indicate the direction estimated parameters deviate from the true values. We have created an additional plot of the results of the simulation study that uses coefficient of variation and have included this plot in section 5.1 of the Supplementary Material as Figure S2. In addition, we have modified how we display the error in the main text, by replacing Figure 2 with a new figure showing the error as the mean and 95% credible interval of the pooled samples of the posterior distribution. The original Figure 2 using the RMSE is now included in section 5.2 of the Supplementary Material as Figure S3.

7. *In the supercritical case (R_e > 1), the same expression holds (see Blumberg and Lloyd-Smith (2013) Epidemics section 2.3.1). Given that this is the case, could you then fit k in the supercritical case of R_e > 1 by just looking at the sizes of the subset of identical clusters, conditioning on their extinction?*

Response:
We thank the reviewer for their question. In our opinion this would be the next step to expand our model. We have thought about ways to include the case $R_g > 1$ into our model, but have not found a satisfactory solution.

We have set out our ideas in chapter 3.1 of the Supplementary Material and added a short remark about this aspect to the manuscript:

In the Supplementary Material:
"Possible extensions of the model

If the genomic reproduction number R_g is larger than 1, then the probability that an identical sequence cluster does not go extinct is strictly bigger than 0. This means that

$$\pi = \sum_{j=0}^{\infty} P(Z = j) < 1.$$

Since we can not observe identical sequence clusters of arbitrary size, this requires additional adjustments to the model in order to fully cope with this situation. We have thought about possible approaches, but have not found a satisfactory solution. In the following we outline our ideas.

Nishiura et al. [7] have shown how to compute the probability of extinction of a branching process with a negative binomially distributed offspring distribution. Their arguments can be extended to compute π. Computing the size distribution of identical sequence clusters conditioned on them being finite is therefore possible but would require some additional computations. In our opinion, the bigger challenge lies elsewhere, namely in the classification of the potential of the clusters to grow to a large size.

Let us assume that $R_g > 1$ for a certain period. We have a number of clusters growing during that period, some already active before, some starting during this period. It is very likely that some of the clusters that have started during this period would continue to grow indefinitely if R_g would stay above 1. In reality, this does not happen. R_g will decrease below 1 at some point and all clusters will go extinct.

However, in our calculations to determine the cluster size distribution from the secondary case distribution, the effective reproduction number, the dispersion parameter, the mutation probability and the testing probability are assumed to remain constant. In order for the cluster size distribution with $R_g > 1$ derived from theory to be consistent with the observed cluster size distribution, one needs to know which clusters from the data would have grown to arbitrary size if R_g stayed above 1. Similarly, to establish consistency between the cluster size distribution derived from theory conditioned on clusters going extinct with the data, one would need to remove those clusters from the data that would not go extinct if R_g stayed above 1.

We have not managed to implement a method to separate clusters that would be likely to continue to grow if R_g stayed above 1 from those that would go extinct. Therefore, we have left this question open for the time being. However, we are convinced that this would be an exciting starting point for further development of our model."

In the manuscript (page 13, lines 378-381):
"We considered ways to enable the model to deal with situations in which R_g is larger than 1, but we have not found a suitable solution. We set out our considerations in the Supplementary Material S1 Text, chapter 3.1."

8. *Is the underlying distribution generated under the estimated R_e and k model consistent with the observed distribution of cluster sizes (at some instance in time)? Visualization of goodness of fit between the model (parameterized by fitting to cluster size data) and the cluster size data would be helpful.*

Response:
This is a valid question. We have already included a comparison between the cluster size data and model-predicted data based on the estimated parameters in chapter 9 ("Posterior predictive check") of the Supplementary Material of our original submission. Furthermore we have added a goodness of fit visualization to chapter 10 ("Goodness of fit") of the Supplementary Material.

We have added a comment to the manuscript (page 9, lines 290-293) to point the readers towards it:

“Furthermore, we have carried out a posterior predictive check and a goodness of fit analysis to assess the compatibility of our simulation of identical sequence clusters based on the estimated parameters with the cluster data from Switzerland, Denmark and Germany (Supplementary Material S1 Text, chapters 9 and 10).”
