# Supplementary material for: Estimating Re and overdispersion in secondary cases from the size of identical sequence clusters of SARS-CoV-2
Source: PLoS Comput Biol. 2025 Apr 15;21(4):e1012960. doi: 10.1371/journal.pcbi.1012960 (PMC12040226; doi:10.1371/journal.pcbi.1012960)
Supplement: Attachment — Submitted filename: reponse_to_review_RandK_Jan2025.pdf [file pcbi.1012960.s008.pdf]

Dear Konstantin Blyuss and Hannah Clapham,

We would like to thank you for the opportunity to once again resubmit our manuscript, “Estimating Re and overdispersion in secondary cases from the size of identical sequence clusters of SARS-CoV-2”, to PLoS Computational Biology.

We sincerely appreciate the further thoughtful reviews, which we have carefully considered and incorporated into the revised manuscript submitted today.

Among our revisions, to help address concerns by Reviewer #1, we have taken additional steps to further clarify the distinction between our work and that of Tran-Kiem and Bedford. We have explicitly highlighted the independent development of our mathematical model and emphasized key differences in our methodological approach, including the use of a Bayesian framework and the application to larger datasets.

Once again, we thank you for this opportunity to re-submit our work, and to include the additional feedback we’ve received, and thank the editors and reviewers in advance for their time.

Sincerely,
Emma B Hodcroft, on behalf of all authors
emma.hodcroft@unibas.ch

Reviewer #1

> *“I appreciate the thoughtful set of revisions that the authors have made based on the reviewer comments. I do have some lingering concerns though.”*

We are thankful to the reviewer for having re-examined our manuscript and are pleased to hear that the reviewer has appreciated our revisions.

> *“I still feel that the manuscript oversells the novelty as compared to the published work of Tran-Kiem and Bedford. If the key distinction is that inference is done in a Bayesian rather than a frequentist framework, then I think this should be made explicit throughout – including in the abstract. (E.g. in the abstract, instead of saying ‘we developed a mathematical model for the distribution of the size of identical sequence clusters, in which we integrated viral transmission, the mutation rate of the virus, and incomplete case-detection’, I would say something like ‘We adapted a model … for Bayesian inference’)”*

We thank the reviewer for this comment. We put some additional effort into explaining more clearly that we did not use the study of Tran-Kiem and Bedford as the basis for our work, but independently derived the same mathematical model (adapted from Blumberg et al.) for the distribution of the size of identical sequence clusters based on the same theoretical concept. We therefore don't feel it would be accurate to write that we "adapted a mathematical model" (in reference to Tran-Kiem and Bedford's work), and thus prefer to keep the expression that we "developed a mathematical model. Furthermore, the framework and setting that we used

to apply the model to infer epidemiological parameters from genomic data differs considerably.

For better clarification, we added the following text to the introduction (lines 58 to 65):

“While concluding this work, Tran-Kiem and Bedford [39] published a similar study deriving the same mathematical model for the distribution of the size of identical sequence clusters based on the same theoretical concept. The development of the respective mathematical models was carried out in parallel and independently by both groups of authors, with considerable differences in the approach to estimating epidemiological parameters from genomic data, including an efficient way of determining genetic clusters, utilizing a Bayesian framework, and applying the method to much larger datasets.”

Major concerns

> *“Here is some feedback regarding my three ‘major’ concerns (replicated below, along with follow-up feedback)*
>
> *1. '… the inference method is predicated on the assumption that R_g is less than ...'*
>
> *I appreciate the additional analysis that the authors did regarding the proportion of clusters that did not end within a month. Within the text, I would add an explicit explanation for why this proportion is important (i.e. explicitly mention the assumption that the genomic reproduction number is less than one, and that the validity of this assumption can be assessed by evaluating the proportion of clusters that did not end within a month).”*

We thank the reviewer for the suggestion to explicitly mention the assumption of $R_g \leq 1$ in the manuscript and link it to the proportion of clusters that did not end within a month.

One the one hand, we added a paragraph to the methods section (lines 184 - 190):

“A key assumption of the model is that $R_g \leq 1$. If $R_g > 1$, then a case in an identical sequence cluster creates on average more than one secondary case that belongs to the same cluster. This results in a non-zero probability that such an identical sequence cluster will not go extinct if allowed to grow over a long time period. This introduces practical issues for inference that are beyond the scope of this work. We present our ideas on how to extend our model to deal with a scenario in which $R_g > 1$ in the Supplementary Material, chapter 3.1.”

On the other hand, we added a sentence to the results section (lines 265 - 267):

“The small proportions of clusters extending beyond one month support the assumption that $R_g \leq 1$ in these particular settings, justifying the choice of a monthly time window.”

> *“2. 'In figure 4 and S3 there are notable time periods where the R estimates from sequences and case counts differ markedly. ….'*
>
> *I appreciate the extra sensitivity analyses the authors have done regarding Re, but the variability of R estimates from case counts compared to sequences is still notable*

(e.g. R of 1.6 from case counts vs 1.1 from sequence data). It would help to learn about potential reasons why there is so much volatility in the case base estimates compared to the genomic estimates."

We thank the reviewer for this comment. We revised one paragraph of the discussion section of our manuscript (lines 407 - 418):

"During some months, our estimates of R_e differ notably from estimates of R_e derived from laboratory-confirmed cases and were not able to fully capture the changes observed in the comparator estimate. On the one hand, this could simply be due to the use of two different methods with different assumptions [52]. On the other hand, this could be due to the assumption of a fixed time window, resulting in monthly rather than daily estimates of R_e. Due to this rather low time resolution during an ongoing epidemic, rapid increases and decreases in R_e cannot be observed and are averaged into a mean estimate for the corresponding time period. As the estimates of R_e based on laboratory-confirmed cases can be obtained on a daily basis, this more traditional method is more suited to detect sudden changes of R_e."

"3. '…the estimates of the dispersion parameter are assumed to be more reliable than the estimates of R_e ….'

I am confused by the explanation provided. To my mind any change in transmission has the capability of influencing both R and k. For example, decrease in R due to immunity is likely going to be patchy (and thus contribute to heterogeneity). Likewise changes in R due to various public health interventions aren't expected to be uniformly applicable across individuals. In fact, some studies have looked at temporal trend of the dispersion parameter and found it can vary more than R (e.g. https://doi.org/10.1371/journal.pcbi.1010078). I recognize that relating the time dependence of R and k is probably beyond the scope of this paper, so I would just be more careful with the wording. I.e. I wouldn't take for granted that k doesn't change. I would rather explain what modeling assumptions this corresponds to and ideally find data to support it (or run some sensitivity analyses to evaluate how important the assumption is)."

We fully agree with the reviewer on this point. We revised the corresponding paragraph in the discussion section of our manuscript (lines 439 - 455):

"In contrast to the increasingly precise estimates of R_e based on laboratory-confirmed cases, estimates of the dispersion parameter k for SARS-CoV-2 have typically come with considerable uncertainty within studies and substantial variability between studies [18–32]. Furthermore, Zhang et al. [53] and Ko et al. [54] showed substantial changes of k over time. Our monthly estimates of k also vary considerably between countries and over time (range: 0.15-0.38), but fit well within the range of earlier estimates. Therefore, our framework appears better suited for estimating k than R_e, as it performs comparably to existing methods for estimating k, while more precise methods are available for estimating R_e using laboratory-confirmed case data."

Minor concerns

> *“Lastly, regarding minor concerns, the one small thing I saw is that I would edit the following, ‘For example, a lower probability of observing an identical sequence cluster of size one would indicate a larger value of k, whereas a higher probability of observing large clusters would indicate a lower value of k’. I would make it clear whether you are talking about the true or inferred value of k. It’s also a little confusing since these scenarios seem similar – i.e. if there is a relatively lower probability of observing isolated clusters, then it seems that there would be a relatively higher probability of observing large clusters?”*

We agree with the reviewer that this section was not clear. We have revised the section (lines 356 - 383) as follows:

“Third, to limit computational complexity of the model we assumed an independent observation process, i.e., infected cases are detected randomly and tested cases are sequenced at random. It is possible that this assumption is not perfectly realistic, which could contribute to the large credible intervals for the estimates of the testing probability. For example, testing and sequencing uptake might cluster in certain settings, such as during contact tracing and outbreak investigations. This would on average lead to a higher actual detection probability for cases contained in a large identical sequence cluster and to a lower actual detection probability for cases contained in a small identical sequence cluster. Therefore, compared to the true underlying size distribution of identical sequence clusters, smaller identical sequence clusters would rather be underrepresented and larger clusters rather overrepresented in the observed data. To find the combination of R_e, k, mutation and testing probability best fitting the data, our model would then be biased to higher values for R_e compared to the true underlying value. In our model of the size distribution of identical sequence clusters, a lower value for k increases the variance and consequently leads to both a higher probability of identical sequence clusters of size one and a higher probability of large identical sequence clusters. We expect that this property limits the impact of a deviation of the observed data from the true underlying distribution on the estimation of k. A less frequent occurrence of smaller clusters points towards a larger k, a more frequent occurrence of larger clusters to a smaller k. In short, we think that the effects on the estimations of R_e and k resulting from this are limited.”

Reviewer #2

> *“Thanks for the changes.”*

We thank the reviewer for the evaluation of our revised manuscript.

Reviewer #3

> *“I thank Hodcroft and coauthors for their thorough response to my initial concerns and suggestions. I think the figures are very much improved over the original ones*

and that the text is more clearly presented. I especially think that the change in the text relating to the per transmission per genome mutation rate M_T has clarified the work."

We thank the reviewer for the feedback and are delighted to hear that we could improve on our initial submission.

"I have four remaining comments/concerns, two of which are major and two of which are minor:"

Major concerns

"1. (MAJOR) Related to my original concern (3):
Lines 170-173 in the main text still state: Since the number of secondary cases follows a negative binomial distribution with mean R_e and dispersion parameter k, the number of secondary cases that belong to the same identical sequence cluster as their source case also follows a negative binomial distribution with mean R_g and dispersion parameter k.
This statement is also restated in Supplemental Material section 2.
As far as I can tell, this statement is not true, and this is what I was getting at in my original concern (3). If this statement were true, then the number of secondary cases that do not belong to the same identical sequence cluster as their source case would also follow a negative binomial distribution with mean μ R_e and dispersion parameter k. But if these statements were true, then the sum of random variables from these distributions should follow a negative binomial distribution with mean R_e and dispersion parameter k, but this is not the case (as the authors agree, given their initial response to my comment). So, it stands that the statement in lines 170-173 is not true. I don't want to stand as a gatekeeper to this manuscript being published, but it really seems to me that if I am correct with this (and I'm happy to be proven wrong), then the whole approach falls apart. If this is the case, one could say, "we make an approximation that R_g follows a negative binomial distribution with mean R_g and dispersion parameter k. This approximation (detail how/under what parameterizations it does best and worst)…" "

We are thankful to the reviewer for checking the underlying theory of our model so carefully.

We agree with the reviewer, that the sum of two independent negative binomially distributed random variables is not negative binomial in general. But in our case, as we discuss in more detail below, the number of secondary cases with and without mutation are not independent and the statement is true.

First, we define some variables we use below to explain where exactly we do not agree with the reviewer's feedback:

- The number of secondary cases $T \sim NB(Re, k)$ with R_e, k > 0
- the number of secondary cases within the same identical sequence cluster as the source case V,
- the number of secondary cases with a mutation relative to the parental sequence W

- and the mutation probability μ ($0 < \mu < 1$).

We disagree with the reviewer's reasoning at the place of the red arrow:

$$T \sim \mathrm{NB}(R_e, k) \implies V \sim \mathrm{NB}(R_g, k) \text{ and } W \sim \mathrm{NB}(\mu R_e, k) \implies V + W \nsim \mathrm{NB}(R_e, k)$$

i.e., that the sum of V and W (which is equal to T) is not negative binomially distributed with mean R_e and dispersion parameter k.

We agree that the following statement is true for M, $h > 0$ and $0 < \beta < 1$:

$$X \sim \mathrm{NB}((1-\beta)M, h)\,,\ Y \sim \mathrm{NB}(\beta M, h)\,,\ X \text{ and } Y \text{ are independent} \implies X + Y \nsim \mathrm{NB}(M, h)$$

i.e., that the sum of X and Y is not negative binomially distributed with mean M and dispersion parameter h if the two variables are independent.

In our paper, V and W are **dependent** random variables because $0 < \mu < 1$. The sum $V + W = T$ follows a negative binomial distribution with mean R_e and dispersion parameter k by definition of T.

To illustrate this, we made some simulations. We set $R_e = 1.2$, $k = 0.3$ and $\mu = 0.1$.

First, we simulated T. Afterwards, we simulated V based on T and computed W as $T - V$ (which holds true by definition of T, V and W).

Second, we simulated two **independent** negative binomial random variables X with mean $(1 - \mu)\, R_e$ and dispersion parameter k and Y with mean $\mu\, R_e$ and dispersion parameter k (panels D and E). Panel F displays the sum of X and Y.

For all simulated random variables, we drew 10,000 samples and displayed them as histograms. The scatter plots represent negative binomial probability mass functions with the following parameters:

- Panels A and F: Mean R_e and dispersion parameter k
- Panels B and D: Mean $(1 - \mu)\, R_e$ and dispersion parameter k
- Panels C and E: Mean $\mu\, R_e$ and dispersion parameter k

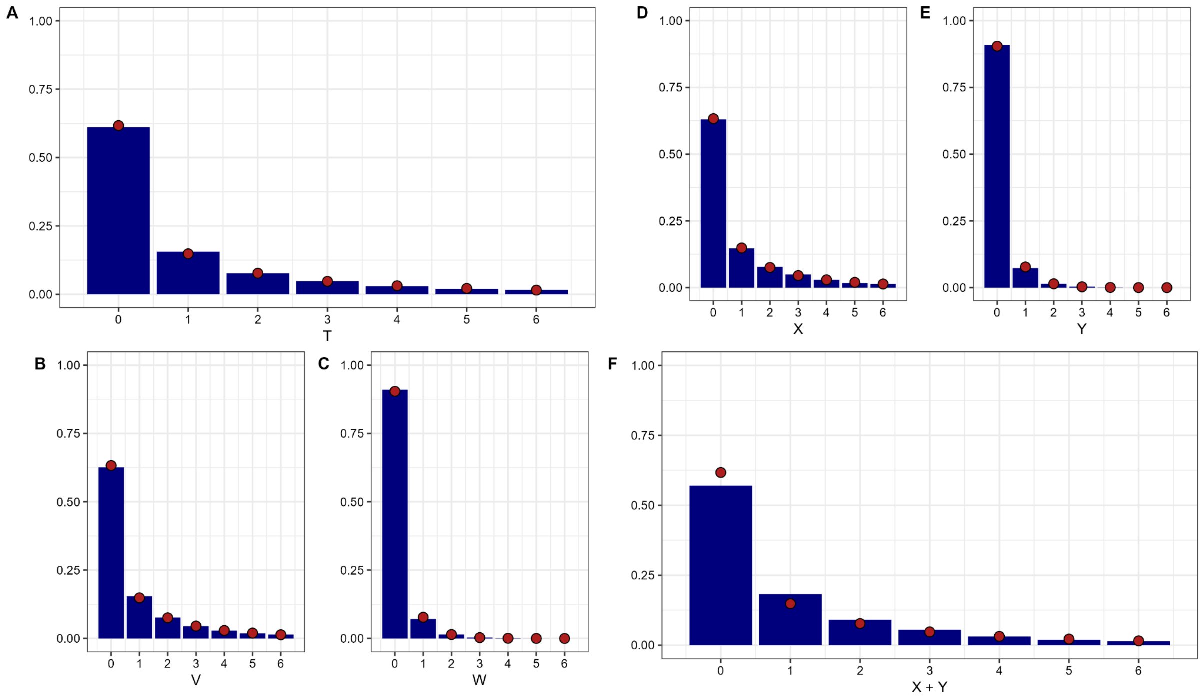

Figure 1: Results of simulations of random variables

The R-code used to obtain these plots can be found on GitHub:
[mwohlfender: R_overdispersion_cluster_size: R / varia / simulation_neg_binom.R]

We have added probability theory statements setting out the above points in detail in chapters 2.2 *Sum of independent negative binomial random variables* and 3.2 *Additional theoretical background* of the Supplementary Material.

> *"2. (MAJOR) Figure 2: currently, the results on the simulated data are interpreted very superficially, in lines 215-217 of the text ("Generally, we found that the error between the true and estimated values of R_e and k is minimized for higher testing and sequencing probabilities. Our method provided reliable estimates of R_e and k as long as R_e was below 1.4.") The benefit of testing an approach on simulated data is that biases and other patterns can be detected! There are several other notable patterns that need to be mentioned and interpreted. First, as testing probability increases, Re estimates tend to increase. This is particularly the case at low R_e. Why is this the case? Second, and this is really important given what the approach presented here is trying to do: estimates of the dispersion parameter k are biased high at low testing probabilities when k < 0.5. Why is this the case? I believe the reason for this is related to my remaining concern (1) above, namely that the assumption that R_g is distributed with mean (1 - μ) R_e and same dispersion parameter k, when the mean is indeed (1 - μ) R_e but the dispersion parameter should no longer be k. Third, testing probability is generally not identifiable. All of these patterns/biases should be explicitly mentioned and interpreted in the main text such that the reader can appreciate the limitations of the approach."*

We thank the reviewer for this comment and admit that the results of the simulation study deserve some additional discussion. We have expanded both the results and the discussion section:

Results (lines 235 - 246):

“Several patterns are recognizable in the results of the simulation study. First, the estimates of R_e tend to increase when the testing probability gets bigger. This effect is stronger in situations with a smaller underlying true effective number. Second, the higher the testing or the sequencing probability, the more precisely the dispersion parameter is estimated. Furthermore,. in some scenarios there is not enough information in the data for the model to move away from the prior distribution of the dispersion parameter, which has mean 0.5. This is particularly evident in situations with lower underlying true values of the effective reproduction number, the testing or the sequencing probability. Overall, the validation showed that our model can be used to estimate R_e and *k* in specific settings, especially in situations with high testing and sequencing probabilities, but fails to provide a reliable estimate of the testing probability.”

Discussion (lines 387 - 406):

“The simulation study showed that our model does not provide equally precise parameter estimates in the entire space of possible values for R_e, *k* and the testing probability. We consider the main reason for this phenomenon to be that in some scenarios, especially when the testing probability and the sequencing probability are low, only a very small number of different sizes of identical sequence clusters appears in the data, in extreme cases only two. The probabilities of larger cluster sizes can become very small very quickly with increasing cluster size, which implies a large amount of data would be required to observe some of them. The more large clusters there are in the data, the more likely the model considers a larger value of R_e. This explains the tendency of increasing estimates of R_e when the testing probability increases in the simulation study. What is also relevant here is our choice of the prior distribution of *k*, a gamma distribution with shape 5 and rate 10, i.e., with mean 0.5. If the model is not able to retrieve information from the data to update the prior distribution, it will stay with it. This is a potential reason why the model fails to provide a precise estimate of *k* in particular when the underlying true k is small. Since for each month there are identical sequence clusters of at least 28 different sizes in Switzerland, 94 in Denmark and 52 in Germany contained in the dataset, we expect that there is sufficient information available to the model to come up with solid estimates of *k* when applied to the real-world data.”

Minor concerns

> *“1. (MINOR) Related to my original concern (4).][vs [] in Table S7A. I thank the authors for clarification! I have never seen this notation. For support that does not include the endpoints, I have always seen “(.., …)” rather than “]…, …[“). Maybe this notation differs across geographic regions. This reviewer is from the US.”*

We thank the reviewer for pointing this out. It is very possible that the standards for the notation of brackets change in different geographical regions. To the authors' knowledge,

both ways of describing intervals without endpoints are used in Switzerland. We have decided to change our convention and use round brackets for the description of intervals without an endpoint in order to make our notation more understandable for readers worldwide.

We have made the corresponding changes in the first paragraph of Chapter 4 of the Supplementary Material and in Table S7A of the Supplementary Material.

> *"2. (MINOR) In Figure 2: Label columns with R_e (rather than R), to make clear that these simulations were run with these effective reproduction numbers (not genomic reproduction numbers of this magnitude)"*

We appreciate that the reviewer has looked at the figures so carefully. We have implemented the proposed change in Figure 2 of the manuscript and in Figures S2, S3 and S4 of the Supplementary Material.
